# Innovative synthesis of drug-like molecules using tetrazole as core building blocks

**DOI:** 10.3762/bjoc.20.85

**Published:** 2024-04-29

**Authors:** Jingyao Li, Ajay L Chandgude, Qiang Zheng, Alexander Dömling

**Affiliations:** 1 Department of Drug Design, University of Groningen, A. Deusinglaan 1, 9713 AV Groningen, The Netherlands; 2 Institute of Molecular and Translational Medicine, Faculty of Medicine and Dentistry and Czech Advanced Technology and Research Institute, Palackӯ University in Olomouc, Olomouc, Czech Republic

**Keywords:** building blocks, green chemistry, multicomponent reaction, Passerini tetrazole reaction, tetrazole, Ugi reaction

## Abstract

Tetrazole is widely utilized as a bioisostere for carboxylic acid in the field of medicinal chemistry and drug development, enhancing the drug-like characteristics of various molecules. Typically, tetrazoles are introduced from their nitrile precursors through late-stage functionalization. In this work, we propose a novel strategy involving the use of diversely protected, unprecedented tetrazole aldehydes as building blocks. This approach facilitates the incorporation of the tetrazole group into multicomponent reactions or other chemistries, aiding in the creation of a variety of complex, drug-like molecules. These innovative tetrazole building blocks are efficiently and directly synthesized using a Passerini three-component reaction (PT-3CR), employing cost-effective and readily available materials. We further showcase the versatility of these new tetrazole building blocks by integrating the tetrazole moiety into various multicomponent reactions (MCRs), which are already significantly employed in drug discovery. This technique represents a unique and complementary method to existing tetrazole synthesis processes. It aims to meet the growing demand for tetrazole-based compound libraries and novel scaffolds, which are challenging to synthesize through other methods.

## Introduction

The efficient synthetic accessibility of drug-like molecules is of key importance. Interestingly, over the years the complexity of molecules entering the market is steadily increasing, supposedly due to the increasing complexity of contemporary molecular targets. A major Swiss API CRO reported that the complexity of chemical synthesis towards novel drugs has almost doubled from eight chemical steps to an average of 14 in 2021 [1]. Therefore, the need for robust reactions compatible with many functional groups in complex molecules is an integral part in contemporary medicinal chemistry and drug development. During preclinical research large screening libraries are integral part of the structure-based drug design and high-throughput screening and facile, efficient synthetic methods to explore diverse chemical spaces are of great help [2–4]. However, due to the vastness of chemical space, generation of ready-to-screen drug-like molecules remains a key challenge in the medicinal chemistry field [5–6].

**Figure 1:**
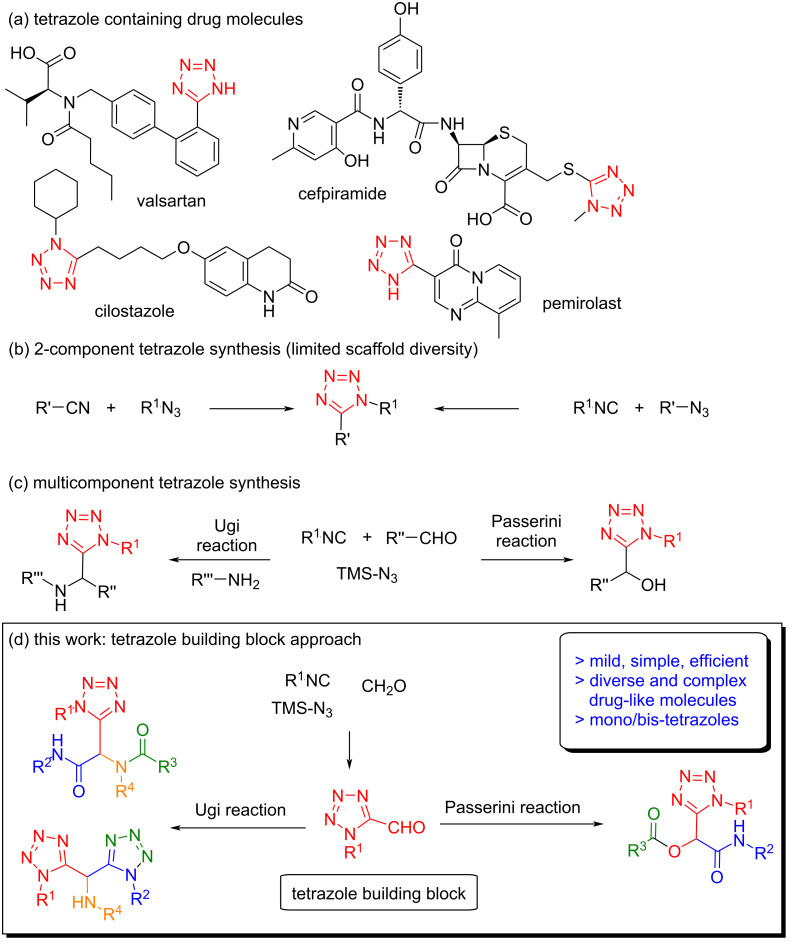
Tetrazole drugs, current assembly strategies, and novel building block strategy.

Tetrazole is considered as a privileged scaffold in pharmaceutical and medicinal chemistry, used as a carboxylic acid bioisostere and a *cis*-amide mimic contributing to improvements in lipophilicity, metabolic stability, conformational rigidity, and potency [7–9]. Recently, the use of the tetrazole moiety in drug development has been increased and exhibited prevalent occurrence in bioactive compounds; being present in more than 20 marketed drugs with a very broad range of biological activities such as anticancer, antitubercular, antibacterial, antiviral, antimalarial, antiallergic, and antihypertensive (Figure 1a) [7,10]. In addition, tetrazoles constitute a diverse range of industrial applications and are extensively used in materials, agriculture, explosives and photography [11–12].

Due to the high synthetic value, significant efforts have been devoted to developing methods for the preparation of the tetrazole scaffolds, in particular recently through multicomponent reactions (MCRs) and mostly Ugi and Passerini reactions [7–9]. Traditionally, due to its charged nature the tetrazole moiety is introduced in a late-stage-modification approach, mostly from nitriles or isocyanides (Figure 1b) [13]. On the other hand, accessing diverse tetrazole scaffolds from MCRs, typically involves first de novo construction of the tetrazole scaffold and subsequent post-modifications (Figure 1c) [14]. In stark contrast, a method for the synthesis of drug-like tetrazole compound libraries involving a building block approach have remained elusive, with success being reported thus far only with the tetrazole amine [14–15]. The use of a tetrazole building block in MCRs would have all the advantages of the de novo approach and furthermore provides an extra efficiency, complexity and diversity in the same number of steps (Figure S1, Supporting Information File 1). Also, it offers a flexibility of placement of the tetrazole moiety in the scaffold and enables additional facile post-modifications due to the protected nature of the tetrazol. Additionally, inclusion of a strategically placed tetrazole and bioisostere replacement of MCR substrate with tetrazole may give rise to molecules with improved drug-like characteristics with nominal weight or size difference. This tetrazole building block approach will allow direct access to ready to screen molecular scaffolds for medicinal chemistry programs to investigate different biological activities [16–18].

Motivated by the shortcomings of current methodologies and the absence of a tetrazole building block approach, we have pursued and report herein the development of a rapid synthetic approach to generate libraries in which the tetrazole ring is conveniently introduced in a scaffold rather than built up de novo for each molecule. We report the synthesis of diverse tetrazole building blocks, which were readily prepared and their unprecedented use in MCRs to access diverse molecular scaffolds. To do that, we first developed an efficient and scalable MCR-based route of the building blocks, in order to provide useful quantities by utilizing diverse isocyanides, azide and paraformaldehyde. Then, this novel tetrazole building block was used in the Passerini and Ugi reactions amenable to convenient, efficient, simple, and fast synthesis of diverse scaffolds library to afford variously functionalized tetrazole-based drug-like molecules.

In order to achieve the synthesis of novel tetrazole building blocks and their use in organic synthesis we envisioned the use of multicomponent reactions in both steps. The use of MCRs provides the benefits of simplicity, speed, complexity, and diversity with the minimum number of steps and with an environmentally friendly nature. First, we focused on the use of the Passerini-tetrazole reaction for the synthesis of tetrazole building blocks which provides the handle of alcohol functionality and further oxidation serves as an oxo component in subsequent MCRs (Figure 1d). The synthesis of oxo-tetrazoles was targeted because of the prevalence of the aldehyde substrate in MCRs and their use in medicinal chemistry literature.

## Results and Discussion

First, we planned to provide a number of orthogonally protected tetrazole carbaldehyde building blocks. This should be accomplished by synthesizing the hydroxymethyl precursors by a Passerini-tetrazole synthesis, followed by oxidation to the aldehyde (Figure 1d). Based on our recently reported highly improved α-hydroxylmethyltetrazole synthesis under mild and save conditions we started to explore the utility of the Passerini-tetrazole reaction with cost-efficient and readily available paraformaldehyde (powder form) [19]. However, paraformaldehyde can sometimes be a challenging substrate for MCRs and this was also the case for the synthesis of oxo-tetrazoles via a Passerini tetrazole reaction [19]. In a model reaction, we investigated benzyl isocyanide (1 equiv), paraformaldehyde (2 equiv) and trimethylsilyl azide (1 equiv) as easily available substrates. Trimethylsilyl azide is considered as a safe replacement of metal azides. We started the solvent optimization with MeOH and H_2_O as solvent system at room temperature, however, it did not yield any product even after 3 days (Table 1, entry 1). The use of DMF to improve the solubility of the paraformaldehyde solid was also unsuccessful to increase the product yield. The increased use of microwave conditions in organic synthesis [20] and our previous promising studies on microwave-assisted MCRs [21], motivated us to use microwave conditions for further optimization. Accordingly, we investigated several sets of reaction conditions with or without solvents at low or high temperature under microwave irradiation and the results are summarized in Table 1. Notably, the reaction with water as a solvent provided a promising yield of 52%, whereas other solvents and conditions resulted only in trace product formation. Combining water with different co-solvents such as MeOH, DCM, CH_3_CN and THF provided products generally with low to moderate yields of 29–69% (Table 1, entries 9–14). The low yield of the desired product is either due to the formation of 1-benzyl-1*H*-tetrazole as side product or low conversion and the oxo-tetrazole component remaining unreacted. To our delight, the use of toluene/water (9:1) as a biphasic solvent system provided quantitative product formation with 90% isolated yield (Table 1, entry 15). Upon trying to reduce the reaction time and performing the reactions at higher temperature, the product yields were significantly reduced. Noteworthy, the synthesis of tetrazole building blocks under the optimized conditions could be easily scaled up to gram amounts.

**Table 1:** Optimization of reaction conditions.

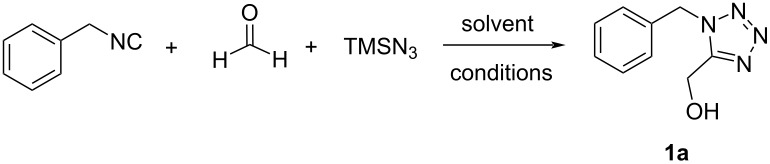					

entry	solvent	conditions	temp	time (min)	yield (%)^a^

1	MeOH/H_2_O (9:1)	rt	rt	3 days	0
2	DMF	rt	rt	3 days	0
3	DCM	mw	60 °C	60	<5
4	MeOH	mw	80 °C	40	<5
5	MeOH	mw	100 °C	20	<5
6	–	mw	100 °C	10	<5
7	H_2_O	mw	100 °C	60	52
8	toluene	mw	150 °C	60	<5
9	MeOH/H_2_O (9:1)	mw	100 °C	60	29
10	MeOH/H_2_O (1:9)	mw	100 °C	60	50
11	DCM/H_2_O (9:1)	mw	80 °C	70	57
12	DCM/H_2_O(9:1)	mw	80 °C	80	69
13	CH_3_CN/H_2_O(9:1)	mw	100 °C	120	51
14	THF/H_2_O(9:1)	mw	100 °C	80	56
**15**	**toluene/H** **_2_** **O (9:1)**	**mw**	**80 °C**	**60**	**90**
16	toluene/H_2_O (9:1)	mw	100 °C	60	80
17	toluene/H_2_O (9:1)	mw	150 °C	60	77

^a^Isolated yields. mw = microwave.

**Scheme 1:**
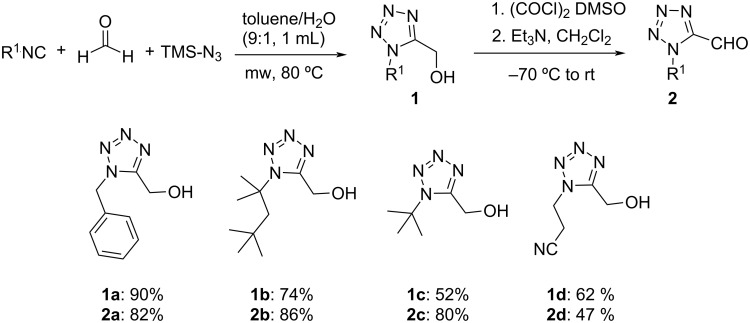
Synthesis of tetrazole building blocks. Isolated yields.

After establishing the optimized conditions, we next examined the substrate scope of the Passerini-tetrazole reaction by varying the isocyanide component (Scheme 1). Aliphatic isocyanides, such as *tert*-octyl and *tert*-butyl isocyanide (**1b** and **1c**) were well-tolerated in the Passerini-tetrazole reaction, leading to the corresponding tetrazole products in moderate to good yields of 74% and 52%, respectively. β-Cyanoethyl isocyanide (**1d**) was also well tolerated providing a good yield (62%). The tolerability of different isocyanides, and the possibility to remove the cleavable isocyanides under different reaction conditions (acidic for the *tert*-octyl and *tert*-butyl isocyanide, basic for β-cyanoethyl isocyanide, or reductive for the benzyl isocyanide) in the developed methodology provides multiple opportunities for various further chemical manipulations, easy access of 1*H*-tetrazole moieties, and great orthogonal functional group compatibility.

First, the tetrazole building blocks were prepared on a multi‐gram scale to access the free alcohol Passerini-tetrazole product. Then, the Swern oxidation [22–24], which is widely exploited and has a wide tolerance of functional groups, was used to convert the tetrazole containing alcohols to aldehydes, in particular for derivatives bearing convertible isocyanides. Most building blocks were well tolerated in Swern oxidation providing moderate to excellent yields of the target aldehydes, except cyclohexyl isocyanide, which decreased the reaction yield and only gave trace amount of product. Importantly, the synthetized carboxaldehyde-tetrazole building blocks are bench stable and can be easily and efficiently scaled-up to gram scale. Noteworthy, 1-substituted tetrazole-5-carbaldehydes are almost elusive from chemical literature [25–29]. Moreover, all synthesis routes described so far are lengthy and involve dangerous chemicals. The 4-step procedure of Sun et al. starts from anilines and involves the hazardous chemicals NaN_3_, DIBAL-H, and CCl_4_ [25]. Moderhack described the synthesis of 1-substituted tetrazole-5-carbaldehyde through a Kröhnke reaction involving lengthy to access 5-chloromethyltetrazole and the well-known carcinogen *p*-nitrosodimethylaniline [27]. Also Ugi described the synthesis of 5-hydroxymethyltetrazoles through a Passerini reaction, however, in low yields, with very long reaction times, and using hazardous aluminum azide [30]. Clearly our novel access to this elusive substance class of 1-substituted tetrazole-5-carbaldehyde outperforms all hitherto described synthetic pathways in terms of economy, sustainability, generalizability, step-count, reaction simplicity, and yields.

With these tetrazole building blocks in hand, we next aimed to investigate the scope and limitations by assembling smaller compound libraries with a high degree of complexity and skeletal diversity. The well described, excellent functional group compatibility and versatility of MCRs along with their promising impact on medicinal chemistry and drug discovery for the library generation prompted us to further use the tetrazole building blocks in MCRs [31–32]. Building on our previous interest in the Passerini reaction involving atypical substrates, we aimed to investigate the unprecedented use of the tetrazole building block as an oxo component in the Passerini reaction to build a library that would possess drug-like molecules and could be easily screened for biological activity and establishment of structure–activity relationships [33]. We envision that the use of tetrazole oxo component in the Passerini reaction will provide more diversity and complexity in the same number of steps and conditions and at the same time provide a simple means to introduce the bioisosteric tetrazole group (Figure S1, Supporting Information File 1).

**Scheme 2:**
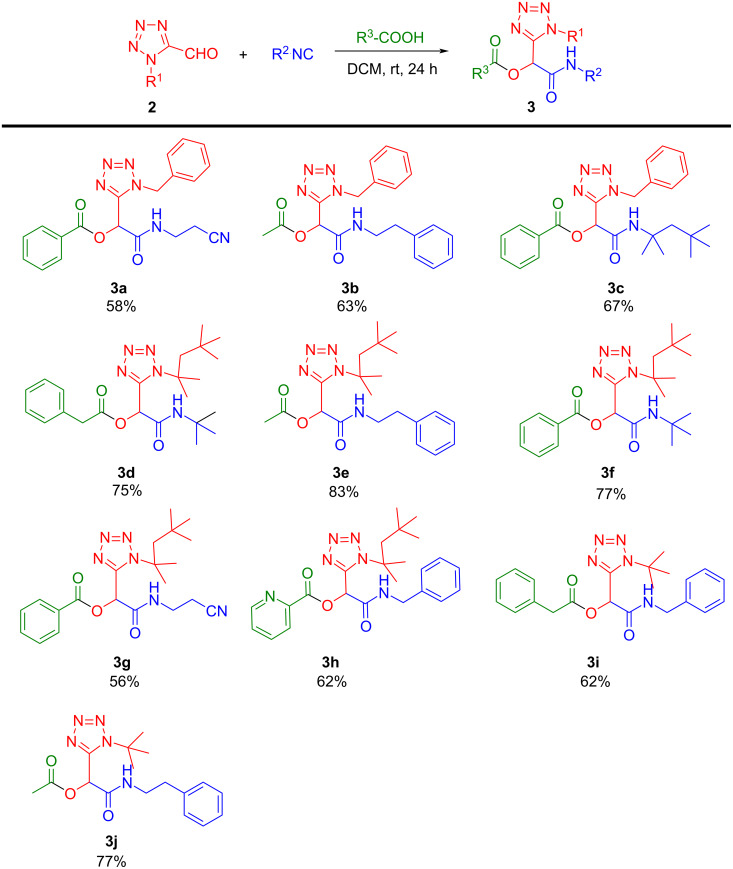
Substrate scope of Passerini products **3**. Isolated yields.

Thus, we examined the ability of the Passerini reaction to incorporate an oxo-tetrazole group with a diverse panel of isocyanide and acid derivatives. Various aliphatic and aromatic acid derivatives are well tolerated leading to the corresponding tetrazole products **3a**–**j** (Scheme 2) in moderate to good yields when the reactions were conducted in DCM at room temperature for 24 hours. Both aliphatic and aromatic substituents on the tetrazole ring of the oxo component were equally well tolerated. Aliphatic aldehydes with aliphatic and aromatic groups reacted smoothly and provided moderate to excellent yields of 58–83%. Among this, the *tert*-octyl-substituted aldehyde exhibited excellent product transformation with a yield of 83% of **3e**. Various isocyanides such as benzyl, phenylethyl, *tert*-octyl and *tert*-butyl isocyanides participated in the reaction with moderate to good yields, mainly lower yields were obtained with β-cyanoethyl isocyanide (e.g., **3g**). In addition, good substrate tolerance was also achieved for the acid component with both aliphatic, aromatic, and heterocyclic acids.

The excellent overall performance of these tetrazole-aldehyde building blocks in Passerini reactions reflected the commendable feasibility of our strategy. These results pave the way to the development of a tetrazole building block approach for other MCRs and can furnish a synthetically important tetrazole-based scaffold.

To even further demonstrate the utility of the developed building block approach, we decided to incorporate an oxo-tetrazole into an Ugi four-component reaction, the most studied and versatile MCR in organic synthesis and drug discovery [31–32]. Despite tremendous exploration, finding novel substrates or bioisosteres as a starting material in the Ugi reaction remains a highly challenging and desired research area [34–36].

**Scheme 3:**
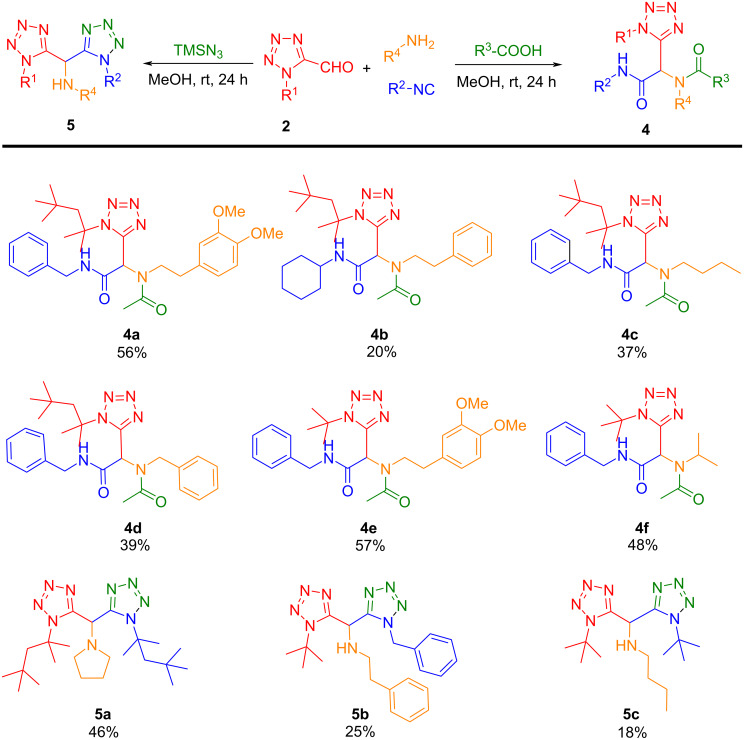
Substrate scope of Ugi products **4** and **5**. Isolated yields.

The use of these atypical oxo-tetrazole building blocks in the Ugi reaction can be a direct and effective way to further explore the tetrazole-based vast chemical space, importantly their use in Ugi reactions can create molecules with diversity and complexity with minor effect on the molecular weight (Figure S1, Supporting Information File 1). The oxo-tetrazole scaffold proved to be successful in the incorporation of the Ugi reaction generating a diverse range of molecules having both mono and ditetrazole scaffolds (Scheme 3). This method is applicable to different amines, isocyanide and acid components including Ugi-4-component and Ugi-tetrazole reactions proving the desired products with low to moderate yields. The Ugi reactions were performed in MeOH at room temperature for 24 hours and without any further optimization. As this oxo-tetrazole building block is a challenging substrate to incorporate in Ugi reactions, it remained mostly intact during the reaction and ultimately provides lower yields as compared to Passerini reactions (Scheme 2).

Although yields for the Ugi reactions are modest with this unusual and unprecedented substrate, this simple and powerful method offers unique tetrazole-based products in a single step that could not be easily obtained by alternative procedures. These low yields could be further improved with the optimization of individual substrates and reaction conditions such as solvent, temperature or assisting techniques such as sonication or microwave. Collectively, this building block strategy opens a new avenue to screen acid bioisostere or amide bioisostere compound libraries to revisit or investigate novel drug targets. The diverse scaffold generation from this method also provides multiple opportunities for various further chemical manipulations which may allow the discovery of novel bioactive small molecule tools.

**Scheme 4:**
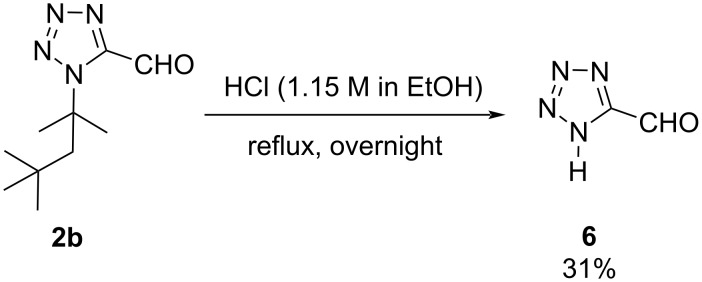
Synthesis of tetrazole building block **6**. Isolated yield.

Recently, the fragment-based drug discovery (FBDD) approach has been emerged as a promising starting point for the new drug discovery, which typically involves the screening of small fragments followed by building the lead molecules by combining active fragments or elaborating the molecules on this fragment. We became interested in investigating the new, easy and direct access to the commercially unavailable 1*H*-oxo-tetrazole fragment. We envisioned the use of tetrazole building blocks having cleavable isocyanide moieties for the synthesis of 5-substitued 1*H*-tetrazoles (Scheme 4). Commercially available *tert*-octyl isocyanide, which has an excellent performance in each step of this strategy, was utilized as a model substrate and further treated with 1.15 M HCl in EtOH under refluxing overnight conditions which provides the desired 1*H*-tetrazole with an acceptable yield of 31%. Noteworthy, only two syntheses of **6** are described in the literature [28–29]. Both methods use dangerous, explosive and toxic hydrazoic acid in a sequential multistep synthesis. In contrast our easy, short and direct method towards **6** provides further opportunities to use this fragment in FBDD or as building block for organic synthesis.

## Conclusion

Our study presented herein comprise a significant advancement in the field of medicinal chemistry and drug development. The introduction of 1*H*-tetrazole as a bioisostere for carboxylic acid has long been recognized for its potential in enhancing drug-like properties [37]. Predominantly, tetrazoles are currently introduced by a late-stage-functionalization approach from their nitrile precursors. This work, however, takes an additional step forward by employing novel 1*H*-protected tetrazole aldehydes as versatile building blocks, a strategy not previously explored. A key innovation of this research lies in the synthesis of these tetrazole building blocks. The use of the Passerini three-component reaction (PT-3CR) utilizing cost-effective and readily available materials not only simplifies the process but also opens up avenues for gram-scale production. This method stands out for its efficiency, simplicity, and environmental friendliness, in contrast to previous syntheses. The incorporation of these tetrazole building blocks into various multicomponent reactions (MCRs) is an additional aspect of our work. This approach significantly expands the chemical space available for drug discovery, offering a means to create complex, drug-like molecules with high skeletal diversity. The study demonstrates that these tetrazole building blocks can be effectively integrated into both Passerini and Ugi reactions, indicating their broad applicability in synthesizing a wide range of molecular scaffolds. Additionally, it is conceivable that the aldehyde group can be introduced in all commonly used chemistries of oxo groups. The potential future applications of this work are vast. The diverse scaffolds prepared can be further diversified, allowing the exploration of new compounds with potential pharmaceutical applications. This opens up exciting possibilities in the development of novel treatments for various diseases of unmet medical needs. Furthermore, the versatility of these tetrazole building blocks could extend beyond medicinal chemistry, finding utility in organic and materials applications. In conclusion, this research represents a significant step forward in the synthesis of drug-like molecules using tetrazole building blocks. Its implications for future drug discovery and development are profound, with the potential to streamline the process of creating new and effective pharmaceuticals. We hope the methodologies and findings described herein will inspire further research and innovation. As the exploration of the synthetic utility of this tetrazole scaffold continues, particularly in other MCRs and post-modification processes, we anticipate more applications in the near future. Investigation of these drug-like molecules for the targets PD-1/PDL-1, caspase-1, and IL-17A are currently in progress in our laboratory.

## Supporting Information

File 1 Experimental procedures, compound characterizations, and NMR spectra.

## Data Availability

All data that supports the findings of this study is available in the published article and/or the supporting information to this article.
