# Supplementary material for: Innovative synthesis of drug-like molecules using tetrazole as core building blocks
Source: Beilstein J Org Chem. 2024 Apr 29;20:950–8. doi: 10.3762/bjoc.20.85 (PMC11070966; doi:10.3762/bjoc.20.85)

BEILSTEIN JOURNAL OF ORGANIC CHEMISTRY

Supporting Information

for

Innovative synthesis of drug-like molecules using tetrazole as core building blocks

Jingyao Li, Ajay L. Chandgude, Qiang Zheng and Alexander Dömling

Beilstein J. Org. Chem. **2024,** *20,* 950–958. doi:10.3762/bjoc.20.85

Experimental procedures, compound characterizations, and NMR spectra

License and Terms: This is a supporting information file under the terms of the Creative Commons Attribution License (https://creativecommons.org/licenses/by/4.0). Please note that the reuse, redistribution and reproduction in particular requires that the author(s) and source are credited and that individual graphics may be subject to special legal provisions.
The license is subject to the *Beilstein Journal of Organic Chemistry* terms and conditions: (https://www.beilstein-journals.org/bjoc/terms)

Contents

General information S2

Experimental procedures S4

^{1}H NMR, ^{13}C NMR and MS spectral data S5

^{1}H NMR and ^{13}C NMR spectra...S13

General information

All isocyanides were made in house by performing the Ugi procedure. Other reagents were available from commercial suppliers (Sigma Aldrich, ABCR, Acros, Fluorochem and AK Scientific) and used without any purification unless otherwise noted. Thin layer chromatography was performed on Fluka precoated silica gel plates (0.20 mm thick, particle size 25 µm). Flash chromatography was performed on a Teledyne ISCO Combiflash Rf, using RediSep Rf Normal-phase Silica Flash Columns (Silica Gel 60 Å, 230– 400 mesh) and on a Reveleris® X2 Flash Chromatography, using Grace® Reveleris Silica flash cartridges (12 grams) and a gradient of petroleum ether/ethyl acetate (0–100%) was applied. Nuclear magnetic resonance spectra were recorded on a Bruker Avance 500 spectrometer. Chemical shifts for ^{1}H NMR were reported relative to TMS (δ 0 ppm) or internal solvent peak ($CDCl_3$ δ 7.26 ppm, DMSO-d_6 δ 2.50 ppm or CD_3OD δ 3.31 ppm) and coupling constants were in hertz (Hz). The following abbreviations were used for spin multiplicity: s = singlet, d = doublet, t = triplet, dt = double triplet, ddd = doublet of double doublet, and m = multiplet. Chemical shifts for ^{13}C NMR reported in ppm relative to the solvent peak ($CDCl_3$ δ 77.23 ppm, MeOD δ 49.00 ppm, DMSO δ 39.52 ppm). Mass spectra were measured on a Waters Investigator Supercritical Fluid Chromatograph with a 3100 MS Detector (ESI) using a solvent system of methanol and CO_2 on a Viridis silica gel column (4.6 × 250 mm, 5 µm particle size) and reported as (*m*/*z*). Electrospray ionization mass spectra (ESIMS) were recorded on a Waters Investigator Semi-prep 15 SFC-MS instrument. Yields given refer to chromatographically purified and spectroscopically pure compounds unless otherwise stated.

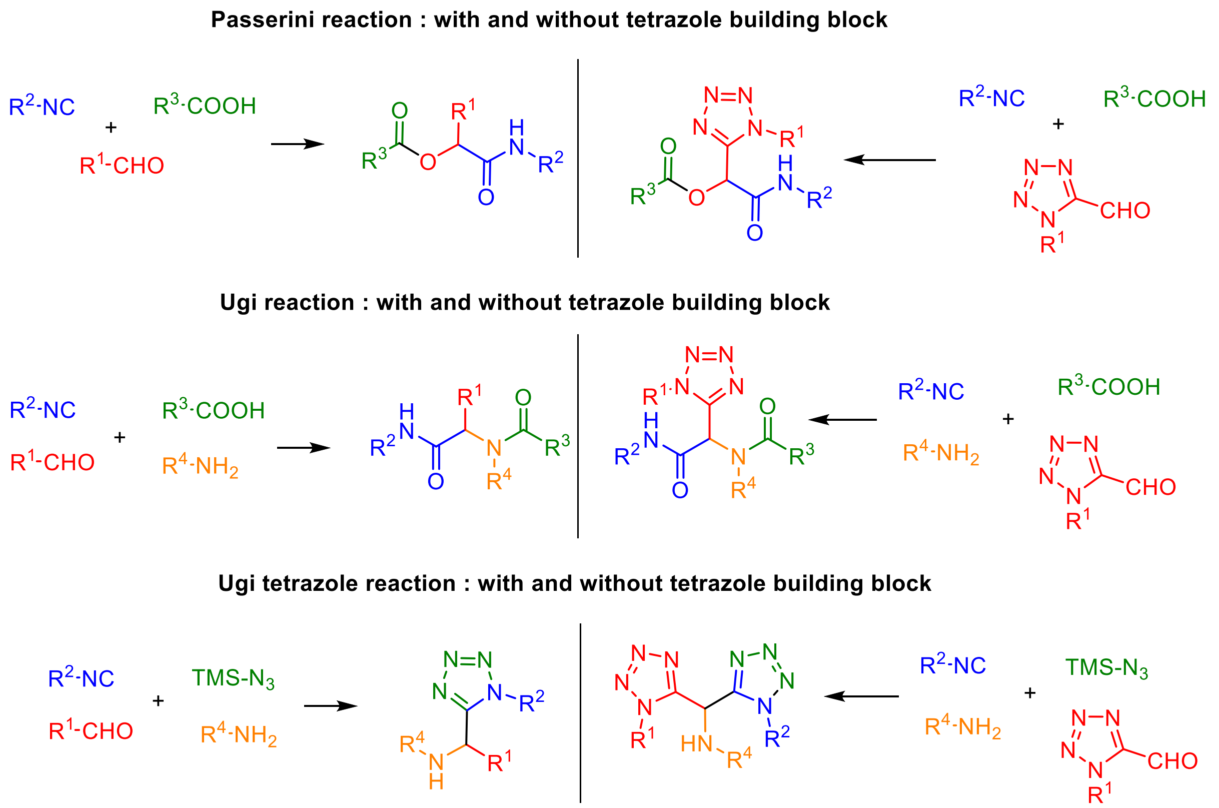

Figure S1: Scaffold diversity and complexity with tetrazole building block use in the Passerini and Ugi reaction. Use of tetrazole building block in MCRs provides extra complexity and diversity in the same number of reaction steps and with minor change in molecular weight.

Experimental procedures

Procedure A: General procedure for the synthesis of alcohols:

A 5 ml microwave vial equipped with a magnetic stir bar was charged with aldehyde (1.0 mmol) and paraformaldehyde (2.0 mmol) in toluene/H_2O (9:1 1 mL) and trimethylsilyl azide (1.0 mmol) was added slowly at room temperature. The vial was sealed with cap containing a septum and subjected to microwave heating at 80 °C [attention: during irradiation, pressure develops] till completion of reaction (reaction monitored by TLC). The solvent was removed under reduced pressure and residue was purified by silica gel flash chromatography using EtOAc–hexane as eluent on to afford the titled product.

Procedure B: General procedure for the synthesis of aldehydes:

To a solution of oxalyl chloride (5 equiv) and 3 Å MS in CH_2Cl_2 at −78 °C under N_2 was added dropwise a solution of DMSO (10 equiv) in CH_2Cl_2. After 15 min a solution of the alcohol (1 equiv) in CH_2Cl_2 was slowly added dropwise. After 30 min, Et_3N (15 equiv) was added dropwise. The reaction was stirred 30 min at −78 °C then slowly allowed to warm to rt. Water was added and stirred for few min to separate organic layer and extracted with DCM. The organic fraction was dried under reduced pressure and residue was purified by silica gel flash chromatography using EtOAc–hexane as eluent on to afford the titled product.

Procedure C: General procedure for the synthesis of Passerini products:

Isocyanide (1.0 equiv) and acid (1.0 equiv) or trimethylsilyl azide (1.0 equiv) were added to a solution of aldehyde (1.0 equiv) in DCM (1 mL/mmol), and the reaction was stirred at room temperature overnight. The solvent was removed under reduced pressure followed by purification by flash chromatography on silica gel using EtOAc–hexane as eluent to afford the titled product.

Procedure D: General procedure for the synthesis of Ugi products:

A solution of aldehyde (1.0 equiv) and amine (1.0 equiv) in methanol (1 mL/mmol) was stirred at room temperature for 30 minutes. Subsequently, isocyanide (1.0 equiv) and acid (1.0 equiv) or trimethylsilyl azide (1.0 equiv) were added and the reaction was stirred at room temperature overnight. The solvent was removed under reduced pressure followed by purification by flash chromatography on silica gel using EtOAc–hexane as eluent to afford the titled product.

Procedure E: General procedure for the synthesis of 1H-tetrazole:

HCl in EtOH (9 equiv) was slowly added to 1-(2,4,4-trimethylpentan-2-yl)-1*H*-tetrazole-5-carbaldehyde. The reaction was refluxed for overnight. Water was added and stirred for a few min to separate organic layer and extracted with DCM. The organic fraction was dried under reduced pressure and residue was purified by silica gel flash chromatography using MeOH-DCM as eluent on to afford the titled product.

^{1}H NMR, ^{13}C NMR and MS spectral data

(1-Benzyl-1*H*-tetrazol-5-yl)methanol (1a)

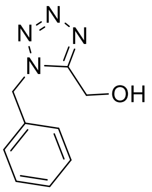

Synthesized according to procedure **A** from 1 mmol reaction as white solid, yield: 171 mg (90%); ^{1}H NMR (500 MHz, $CDCl_3$) δ 7.41 – 7.34 (m, 3H), 7.29 (dd, *J* = 7.1, 2.0, 2H), 5.66 (s, 2H), 4.84 (s, 2H), 3.37 (br s, 1H). ^{13}C NMR (126 MHz, $CDCl_3$) δ 153.6, 133.1, 129.2, 129.1, 128.0, 53.9, 51.4.

(1-(2,4,4-Trimethylpentan-2-yl)-1*H*-tetrazol-5-yl)methanol (1b)

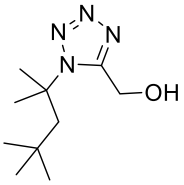

Synthesized according to procedure **A** from 1 mmol reaction as white solid, yield: 158mg (74%); ^{1}H NMR (500 MHz, $CDCl_3$) δ 5.06 (d, *J* = 6.3, 2H), 4.15 (t, *J* = 6.6, 1H), 2.02 (s, 2H), 1.83 (s, 6H), 0.78 (s, 9H). ^{13}C NMR (126 MHz, $CDCl_3$) δ 154.0, 65.1, 55.4, 53.5, 31.7, 30.6, 29.9. ^{13}C NMR (126 MHz, $CDCl_3$) δ 154.00 65.09 55.37 53.51 31.65 30.61 29.94.

(1-(*tert*-Butyl)-1*H*-tetrazol-5-yl)methanol (1c)

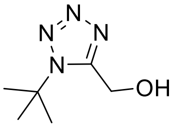

Synthesized according to procedure **A** from 1 mmol reaction as white solid, yield: 81mg (52%); ^{1}H NMR (500 MHz, $CDCl_3$) δ 5.05 (d, *J* = 6.6, 2H), 4.08 – 3.93 (m, 1H), 1.77 (s, 9H). ^{13}C NMR (126 MHz, $CDCl_3$) δ 153.5, 61.8, 55.0, 29.6.

3-(5-(Hydroxymethyl)-1*H*-tetrazol-1-yl)propanenitrile (1d)

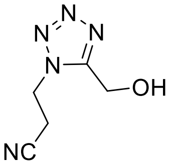

Synthesized according to procedure **A** from 1 mmol reaction as white solid, yield: 190 mg (62%); ^{1}H NMR (500 MHz, MeOD) δ 4.01 (t, *J* = 2.8, 2H), 3.58 – 3.46 (m, 2H), 2.70 (td, *J* = 6.6, 2.9, 2H). ^{13}C NMR (126 MHz, MeOD) δ 175.7, 119.3, 62.5, 36.0, 18.5. MS (ESI) m/z calculated $[M+H]^+$: 154.07; found $[M+H]^+$: 154.06.

1-Benzyl-1*H*-tetrazole-5-carbaldehyde (2a)

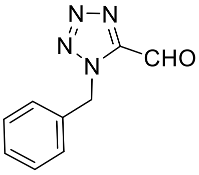

Synthesized according to procedure **B** from 1 mmol reaction as colorless liquid, yield: 154 mg (82%); ^{1}H NMR (500 MHz, $CDCl_3$) δ 10.25 (s, 1H), 7.42 – 7.32 (m, 6H), 5.87 (s, 2H). ^{13}C NMR (126 MHz, $CDCl_3$) δ 179.5, 148.7, 132.9, 129.3, 129.1, 129.0, 128.6, 52.7 ppm. MS (ESI) m/z calculated $[M+H]^+$: 189.07; found $[M+H]^+$: 189.14.

1-(2,4,4-Trimethylpentan-2-yl)-1*H*-tetrazole-5-carbaldehyde (2b)

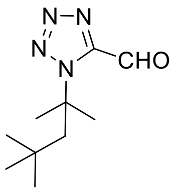

Synthesized according to procedure **B** from 1 mmol reaction as colorless liquid, yield: 181 mg (86%); ^{1}H NMR (500 MHz, $CDCl_3$) δ 10.30 (s, 1H), 0.81 (s, 2H), 0.78 (s, 4H), 0.72 (s, 9H). ^{13}C NMR (126 MHz, $CDCl_3$) δ 179.2, 140.6, 54.3, 51.2, 30.6, 29.9, 29.5 ppm.

1-(*tert*-Butyl)-1*H*-tetrazole-5-carbaldehyde (2c)

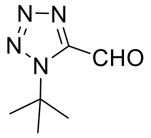

Synthesized according to procedure **B** from 1mmol reaction as pale yellow liquid, yield: 123 mg (80%); ^{1}H NMR (500 MHz, $CDCl_3$) δ 10.30 (s, 1H), 1.78 (s, 9H). ^{13}C NMR (126 MHz, $CDCl_3$) δ 179.1, 150.6, 64.1, 28.7 ppm.

3-(5-Formyl-1*H*-tetrazol-1-yl)propanenitrile (2d)

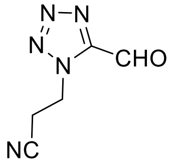

Synthesized according to procedure **B** from 1 mmol reaction as colorless liquid, yield: 71 mg (47%); ^{1}H NMR (500 MHz, $CDCl_3$) δ 8.86 (s, 1H), 4.80 (t, *J* = 6.5, 2H), 3.13 (t, *J* = 6.5, 2H).

1-(1-Benzyl-1*H*-tetrazol-5-yl)-2-((2-cyanoethyl)amino)-2-oxoethyl benzoate (3a)

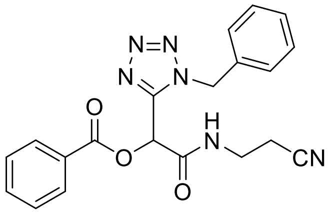

Synthesized according to procedure **C** from 0.5 mmol reaction as white viscous liquid, yield: 113 mg (58%); ^{1}H NMR (500 MHz, $CDCl_3$) δ 8.03 (t, *J* = 6.1, 1H), 7.75 (d, *J* = 7.3, 2H), 7.49 (t, *J* = 7.5, 1H), 7.35 – 7.27 (m, 5H), 7.23 (dd, *J* = 7.2, 1.7, 2H), 6.67 (s, 1H), 5.90 (d, *J* = 15.5, 1H), 5.81 (d, *J* = 15.6, 1H), 3.66 – 3.42 (m, 2H), 2.58 (t, *J* = 6.6, 2H). ^{13}C NMR (126 MHz, $CDCl_3$) δ 179.6, 164.8, 164.6, 150.6, 134.2, 133.2, 130.1, 129.1, 128.8, 128.6, 127.7, 127.4, 117.9, 65.9, 52.0, 35.8, 17.9 ppm; MS (ESI) m/z calculated $[M+Na]^+$: 413.13; found $[M+Na]^+$: 413.21.

1-(1-Benzyl-1*H*-tetrazol-5-yl)-2-oxo-2-(phenethylamino)ethyl acetate (3b)

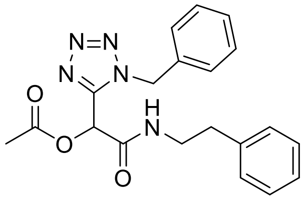

Synthesized according to procedure **C** from 0.5 mmol reaction as white solid, yield: 119 mg (63%); ^{1}H NMR (500 MHz, $CDCl_3$) δ 7.40 – 7.32 (m, 3H), 7.28 – 7.24 (m, 4H), 7.24 – 7.17 (m, 1H), 7.16 – 7.07 (m, 2H), 6.93 (t, *J* = 6.0 Hz, 1H), 6.27 (s, 1H), 5.83 – 5.67 (m, 2H), 3.64 – 3.39 (m, 2H), 2.79 (t, *J* = 7.1 Hz, 2H), 1.96 (s, 3H). ^{13}C NMR (126 MHz, $CDCl_3$) δ 168.48, 163.80, 150.70, 138.14, 133.20, 129.04, 128.86, 128.77, 128.70, 128.67, 127.93, 126.69, 65.57, 51.89, 40.86, 35.28, 20.18.MS (ESI) m/z calculated $[M+H]^+$: 380.17; found $[M+H]^+$: 380.27.

1-(1-Benzyl-1*H*-tetrazol-5-yl)-2-oxo-2-((2,4,4-trimethylpentan-2-yl)amino)ethyl benzoate (3c)

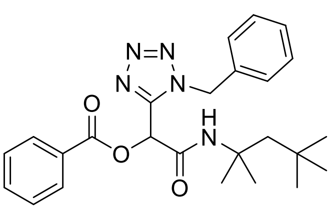

Synthesized according to procedure **C** from 0.5 mmol reaction as white solid, yield: 150 mg (67%);^{1}H NMR (500 MHz, $CDCl_3$) δ 7.89 – 7.80 (m, 2H), 7.64 – 7.51 (m, 1H), 7.38 (t, *J* = 7.8 Hz, 2H), 7.34 – 7.30 (m, 3H), 7.30 – 7.26 (m, 2H), 6.63 (s, 1H), 6.56 (s, 1H), 6.03 – 5.84 (m, 2H), 1.71 (q, *J* = 15.0 Hz, 2H), 1.42 (d, *J* = 7.4 Hz, 6H), 0.91 (s, 9H). ^{13}C NMR (126 MHz, $CDCl_3$) δ 164.43, 162.60, 150.82, 134.12, 133.34, 129.96, 129.08, 128.74, 128.63, 127.78, 127.76, 66.49, 56.42, 51.87, 31.59, 31.32, 28.88, 28.69. MS (ESI) m/z calculated $[M+H]^+$: 450.25; found $[M+H]^+$: 450.36.

2-(*tert*-Butylamino)-2-oxo-1-(1-(2,4,4-trimethylpentan-2-yl)-1*H*-tetrazol-5-yl)ethyl 2-phenylacetate (3d)

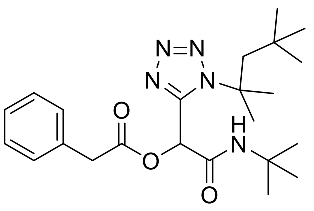

Synthesized according to procedure **C** from 1 mmol reaction as white solid, yield: 322 mg (75%); ^{1}H NMR (500 MHz, $CDCl_3$) δ 7.40 – 7.32 (m, 2H), 7.32 – 7.29 (m, 3H), 6.54 (s, 1H), 6.06 (s, 1H), 3.75 (d, *J* = 2.0 Hz, 2H), 2.20 (d, *J* = 15.3 Hz, 1H), 2.03 (d, *J* = 15.3 Hz, 1H), 1.91 (s, 3H), 1.83 (s, 3H), 1.20 (s, 9H), 0.78 (s, 9H). ^{13}C NMR (126 MHz, $CDCl_3$) δ 169.14, 163.20, 150.89, 132.53, 129.24, 129.03, 127.77, 66.68, 66.60, 54.26, 51.89, 41.00, 31.63, 30.53, 30.05, 28.28. MS (ESI) m/z calculated $[M+H]^+$: 430.28; found $[M+H]^+$: 430.40.

2-Oxo-2-(phenethylamino)-1-(1-(2,4,4-trimethylpentan-2-yl)-1*H*-tetrazol-5-yl)ethyl acetate (3e)

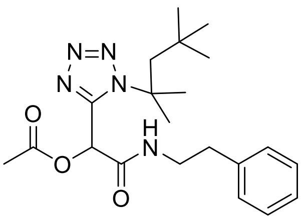

Synthesized according to procedure **C** from 1 mmol reaction as white solid, yield: 333 mg (83%); ^{1}H NMR (500 MHz, $CDCl_3$) δ 7.31 – 7.29 (m, 1H), 7.28 – 7.27 (m, 1H), 7.24 – 7.19 (m, 1H), 7.19 – 7.15 (m, 2H), 7.10 (t, *J* = 6.1 Hz, 1H), 6.66 (s, 1H), 3.68 – 3.57 (m, 1H), 3.45 – 3.35 (m, 1H), 2.82 (td, *J* = 7.1, 1.8 Hz, 2H), 2.21 (d, *J* = 15.3 Hz, 1H), 2.10 – 2.03 (m, 4H), 1.92 (s, 3H), 1.86 (s, 3H), 0.80 (s, 9H). ^{13}C NMR (126 MHz, $CDCl_3$) δ 168.71, 164.34, 150.94, 138.28, 128.82, 128.64, 126.63, 66.78, 66.22, 54.24, 40.71, 35.33, 31.66, 30.54, 30.11, 20.45. MS (ESI) m/z calculated $[M+Na]^+$: 424.33; found $[M+Na]^+$: 424.35.

2-(*tert*-Butylamino)-2-oxo-1-(1-(2,4,4-trimethylpentan-2-yl)-1*H*-tetrazol-5-yl)ethyl benzoate (3f)

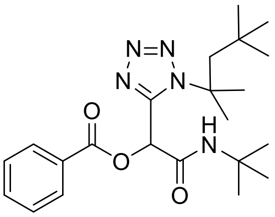

Synthesized according to procedure **C** from 0.5 mmol reaction as white solid, yield: 160 mg (77%); ^{1}H NMR (500 MHz, $CDCl_3$) δ 8.09 – 8.03 (m, 2H), 7.63 – 7.57 (m, 1H), 7.46 (t, *J* = 7.9 Hz, 2H), 6.80 (s, 1H), 6.43 (s, 1H), 2.17 (d, *J* = 15.2 Hz, 1H), 2.05 (d, *J* = 15.2 Hz, 1H), 1.93 (s, 3H), 1.88 (s, 3H), 1.35 (s, 9H), 0.74 (s, 9H). ^{13}C NMR (126 MHz, $CDCl_3$) δ 164.44, 163.51, 150.95, 134.34, 129.98, 128.84, 127.87, 66.95, 66.53, 54.22, 52.10, 31.62, 30.48, 30.35, 28.44. MS (ESI) m/z calculated $[M+H]^+$: 416.27; found $[M+H]^+$: 416.37.

2-((2-Isocyanoethyl)amino)-2-oxo-1-(1-(2,4,4-trimethylpentan-2-yl)-1*H*-tetrazol-5-yl)ethyl benzoate (3g)

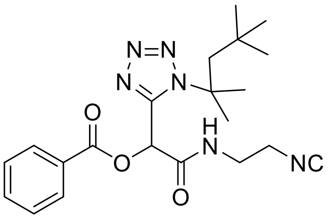

Synthesized according to procedure **C** from 1 mmol reaction as white solid, yield: 231 mg (56%); ^{1}H NMR (500 MHz, $CDCl_3$) δ 8.12 – 8.05 (m, 2H), 7.64 (t, *J* = 7.5, 1H), 7.47 (t, *J* = 7.8, 2H), 7.33 (t, *J* = 6.0, 1H), 6.95 (s, 1H), 3.71 – 3.61 (m, 1H), 3.59 – 3.47 (m, 1H), 2.74 – 2.54 (m, 2H), 2.14 (q, *J* = 15.3, 2H), 1.96 (s, 3H), 1.91 (s, 3H), 0.80 (s, 9H). ^{13}C NMR (126 MHz, $CDCl_3$) δ 165.5, 164.5, 150.4, 134.6, 130.2, 128.9, 127.5, 117.4, 66.8, 66.5, 54.4, 35.8, 31.7, 30.5, 30.4, 30.3, 18.0 ppm; MS (ESI) m/z calculated $[M-H]^-$: 411.22; found $[M-H]^-$: 411.29.

2-(Benzylamino)-2-oxo-1-(1-(2,4,4-trimethylpentan-2-yl)-1*H*-tetrazol-5-yl)ethyl picolinate (3h)

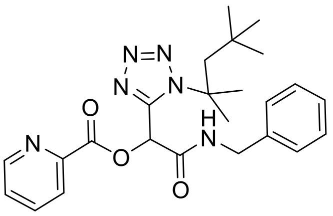

Synthesized according to procedure **C** from 0.5 mmol reaction as white solid, yield: 140 mg (62%); ^{1}H NMR (500 MHz, $CDCl_3$) δ 8.66 – 8.61 (m, 1H), 8.24 – 8.16 (m, 1H), 8.08 (d, *J* = 7.8 Hz, 1H), 7.79 (td, *J* = 7.8, 1.8 Hz, 1H), 7.48 (ddd, *J* = 7.6, 4.7, 1.2 Hz, 1H), 7.29 – 7.27 (m, 1H), 7.26 – 7.23 (m, 2H), 7.23 – 7.18 (m, 1H), 6.99 (s, 1H), 4.49 (d, *J* = 6.0 Hz, 2H), 2.26 (d, *J* = 15.2 Hz, 1H), 2.14 (d, *J* = 15.2 Hz, 1H), 1.98 (s, 3H), 1.93 (s, 3H), 0.82 (s, 9H). ^{13}C NMR (126 MHz, $CDCl_3$) δ 164.57, 163.38, 150.78, 150.05, 145.91, 137.37, 137.29, 128.61, 128.01, 127.57, 127.46, 125.86, 67.30, 66.97, 54.39, 43.47, 31.69, 30.58, 30.42, 30.11. MS (ESI) m/z calculated $[M+Na]^+$: 473.23; found $[M+ Na]^+$: 473.33.

2-(Benzylamino)-1-(1-(*tert*-butyl)-1*H*-tetrazol-5-yl)-2-oxoethyl 2-phenylacetate (3i)

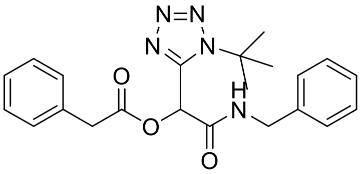

Synthesized according to procedure **C** from 1 mmol reaction as white solid, yield: 252 mg (62%); ^{1}H NMR (500 MHz, $CDCl_3$) δ 7.34 – 7.27 (m, 3H), 7.25 – 7.20 (m, 6H), 7.17 (t, *J* = 6.0 Hz, 1H), 6.67 (s, 1H), 4.39 (dd, *J* = 14.8, 6.0 Hz, 1H), 4.33 (dd, *J* = 15.0, 5.9 Hz, 1H), 3.75 (d, *J* = 15.6 Hz, 1H), 3.69 (d, *J* = 15.6 Hz, 1H), 1.77 (s, 9H). ^{13}C NMR (126 MHz, $CDCl_3$) δ 169.53, 164.46, 150.60, 137.07, 132.50, 129.37, 129.26, 128.87, 128.73, 127.69, 127.66, 66.33, 63.32, 43.44, 40.65, 30.15. MS (ESI) m/z calculated $[M+Na]^+$: 430.19; found $[M+Na]^+$: 430.31.

1-(1-(*tert*-Butyl)-1*H*-tetrazol-5-yl)-2-oxo-2-(phenethylamino)ethyl acetate (3j)

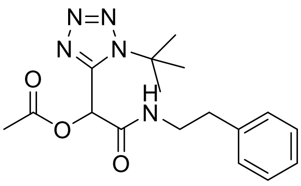

Synthesized according to procedure **C** from 1 mmol reaction as white solid, yield: 266 mg (77%); ^{1}H NMR (500 MHz, $CDCl_3$) δ 7.30 – 7.26 (m, 2H), 7.24 – 7.19 (m, 1H), 7.19 – 7.15 (m, 2H), 7.12 (t, *J* = 6.1 Hz, 1H), 6.61 (s, 1H), 3.62 – 3.56 (m, 1H), 3.51 – 3.42 (m, 1H), 2.83 (td, *J* = 7.1, 3.1 Hz, 2H), 2.10 (s, 3H), 1.83 (s, 9H). ^{13}C NMR (126 MHz, $CDCl_3$) δ 168.77, 164.32, 150.78, 138.28, 128.79, 128.62, 126.61, 66.23, 63.28, 40.76, 35.31, 30.17, 20.43.MS (ESI) m/z calculated $[M+Na]^+$: 368.17; found $[M+Na]^+$: 368.30.

***N*-Benzyl-2-(*N*-(3,4-dimethoxyphenethyl)acetamido)-2-(1-(2,4,4-trimethylpentan-2-yl)-1*H*-tetrazol-5-yl)acetamide (4a)**

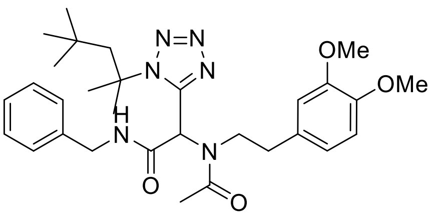

Synthesized according to procedure **D** from 0.5 mmol reaction as white solid, yield: 154 mg (56%); ^{1}H NMR (500 MHz, $CDCl_3$) δ 7.29 (d, *J* = 7.4, 2H), 7.26 – 7.21 (m, 3H), 7.15 (s, 1H), 6.77 (d, *J* = 8.1, 1H), 6.68 (s, 1H), 6.64 (d, *J* = 8.1, 1H), 6.46 (s, 1H), 4.48 (d, *J* = 5.7, 2H), 3.90 (s, 3H), 3.85 (s, 3H), 3.83 – 3.76 (m, 1H), 3.76 – 3.66 (m, 1H), 2.67 (td, *J* = 12.3, 5.2, 1H), 2.26 (s, 3H), 2.15 (td, *J* = 12.3, 5.3, 1H), 2.03 (d, *J* = 2.4, 2H), 1.88 (s, 3H), 1.80 (s, 3H), 0.79 (s, 9H). ^{13}C NMR (126 MHz, $CDCl_3$) δ 171.9, 165.5, 161.1, 150.5, 149.2, 148.0, 137.2, 130.1, 128.8, 128.7, 127.8, 127.8, 127.7, 127.6, 120.6, 112.0, 111.5, 66.3, 56.0, 55.9, 53.6, 52.1, 48.9, 44.2, 35.8, 31.6, 30.7, 30.2, 30.0, 21.5 ppm; MS (ESI) m/z calculated $[M+Na]^+$: 573.32; found $[M+Na]^+$: 573.40.

***N*-Cyclohexyl-2-(*N*-phenethylacetamido)-2-(1-(2,4,4-trimethylpentan-2-yl)-1*H*-tetrazol-5-yl)acetamide (4b)**

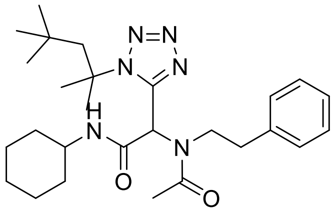

Synthesized according to procedure **D** from 1 mmol reaction as white solid, yield: 96 mg (20%); ^{1}H NMR (500 MHz, CDCl$_3$) δ 7.35 – 7.27 (m, 2H), 7.27 – 7.21 (m, 1H), 7.21 – 7.13 (m, 2H), 7.05 (s, 1H), 6.14 (d, *J* = 8.0 Hz, 1H), 3.90 – 3.77 (m, 3H), 2.87 – 2.78 (m, 1H), 2.50 – 2.40 (m, 1H), 2.28 (s, 3H), 2.10 (d, *J* = 15.4 Hz, 1H), 2.06 (d, *J* = 15.1 Hz, 1H), 1.98 – 1.91 (m, 1H), 1.89 (s, 3H), 1.84 (s, 3H), 1.75 – 1.55 (m, 4H), 1.43 – 1.29 (m, 2H), 1.26 – 1.06 (m, 3H), 0.83 (s, 9H). ^{13}C NMR (126 MHz, CDCl$_3$) δ 171.89, 164.26, 150.78, 137.56, 128.77, 128.68, 126.84, 66.22, 53.52, 52.40, 49.23, 48.70, 36.26, 32.69, 32.63, 31.67, 30.72, 30.32, 30.01, 25.34, 24.60, 21.53. MS (ESI) m/z calculated [M+Na]$^+$: 505.33; found [M+Na]$^+$: 505.42.

***N*-Benzyl-2-(*N*-butylacetamido)-2-(1-(2,4,4-trimethylpentan-2-yl)-1*H*-tetrazol-5-yl)acetamide (4c)**

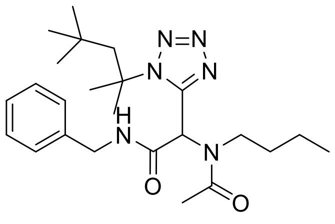

Synthesized according to procedure **D** from 1 mmol reaction as white solid, yield: 164 mg (37%); ^{1}H NMR (500 MHz, CDCl$_3$) δ 7.32 – 7.27 (m, 2H), 7.27 – 7.22 (m, 3H), 7.02 (s, 1H), 4.44 (dd, *J* = 5.9, 2.1 Hz, 2H), 3.67 – 3.52 (m, 2H), 2.12 (s, 3H), 2.01 (s, 2H), 1.82 (s, 3H), 1.77 (s, 3H), 1.42 – 1.32 (m, 1H), 1.32 – 1.23 (m, 1H), 1.22 – 1.14 (m, 2H), 1.05 – 0.92 (m, 1H), 0.82 (t, *J* = 7.3 Hz, 3H), 0.78 (s, 9H). ^{13}C NMR (126 MHz, CDCl$_3$) δ 172.04, 165.61, 150.73, 137.39, 128.66, 127.91, 127.59, 66.04, 53.50, 52.37, 47.05, 44.08, 31.92, 31.59, 30.63, 30.03, 29.88, 21.32, 20.02, 13.50. MS (ESI) m/z calculated [M+Na]$^+$: 465.30; found [M+Na]$^+$: 465.42.

***N*-Benzyl-2-(*N*-benzylacetamido)-2-(1-(2,4,4-trimethylpentan-2-yl)-1*H*-tetrazol-5-yl)acetamide (4d)**

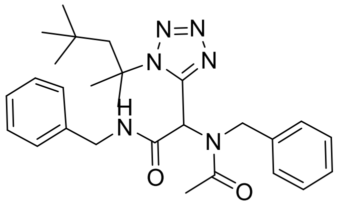

Synthesized according to procedure **D** from 1 mmol reaction as white solid, yield: 186 mg (39%); ^{1}H NMR (500 MHz, CDCl$_3$) δ 7.29 – 7.25 (m, 2H), 7.24 – 7.18 (m, 4H), 7.12 (s, 1H), 7.11 – 7.03 (m, 4H), 7.03 – 6.95 (m, 1H), 5.07 (d, *J* = 17.9 Hz, 1H), 4.98 (d, *J* = 17.9 Hz, 1H), 4.24 (dd, *J* = 14.6, 6.0 Hz, 1H), 4.03 (dd, *J* = 14.6, 5.3 Hz, 1H), 2.07 (s, 3H), 2.06 – 2.01 (m, 2H), 1.79 (s, 3H), 1.76 (s, 3H), 0.78 (s, 10H). ^{13}C NMR (126 MHz, CDCl$_3$) δ 173.13, 164.98, 150.95, 137.02, 136.88, 128.73, 128.66, 127.97, 127.66, 127.43, 126.16, 66.25, 53.75, 53.57, 50.66, 44.13, 31.63, 30.65, 29.99, 29.83, 22.13. MS (ESI) m/z calculated [M+Na]$^+$: 499.28; found [M+Na]$^+$: 499.38.

N-Benzyl-2-(1-(tert-butyl)-1H-tetrazol-5-yl)-2-(N-(3,4-dimethoxyphenethyl)acetamido)acetamide (4e)

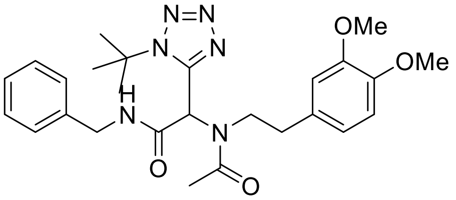

Synthesized according to procedure **D** from 0.5 mmol reaction as white solid, yield: 141 mg (57%); ^{1}H NMR (500 MHz, $CDCl_3$) δ 7.37 – 7.19 (m, 6H), 6.81 – 6.72 (m, 2H), 6.61 (s, 1H), 6.59 (s, 1H), 5.29 (s, 1H), 4.47 (dd, J = 7.7, 6.1, 2H), 3.87 (s, 3H), 3.83 (s, 3H), 3.81 – 3.75 (m, 1H), 3.75 – 3.65 (m, 1H), 2.64 (td, J = 12.4, 5.2, 1H), 2.22 (s, 3H), 2.10 – 2.04 (m, 1H), 1.75 (s, 9H). ^{13}C NMR (126 MHz, $CDCl_3$) δ 172.0, 165.5, 150.4, 149.1, 147.9, 137.3, 129.9, 128.7, 127.8, 127.7, 120.5, 111.9, 111.4, 62.6, 55.9, 55.9, 52.2, 48.9, 44.2, 35.8, 29.8, 29.7, 21.5 ppm; MS (ESI) m/z calculated $[M+Na]^+$: 517.25; found $[M+Na]^+$: 517.37.

N-Benzyl-2-(1-(tert-butyl)-1H-tetrazol-5-yl)-2-(N-isopropylacetamido)acetamide (4f)

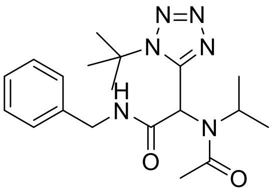

Synthesized according to procedure **D** from 0.5 mmol reaction as white solid, yield: 89 mg (48%); ^{1}H NMR (500 MHz, $CDCl_3$) δ 7.35 – 7.31 (m, 2H), 7.30 – 7.27 (m, 3H), 5.30 (s, 2H), 4.51 – 4.44 (m, 2H), 2.24 (s, 3H), 1.80 (s, 2H), 1.74 (s, 9H), 1.30 (d, J = 7.0, 3H), 1.26 (br s, 3H). ^{13}C NMR (126 MHz, $CDCl_3$) δ 165.9, 165.8, 150.6, 128.8, 128.1, 127.8, 66.1, 53.4, 44.3, 43.9, 30.0, 29.9, 29.7 ppm; MS (ESI) m/z calculated $[M+Na]^+$: 395.22; found $[M+Na]^+$: 395.31.

5,5'-(Pyrrolidin-1-ylmethylene)bis(1-(2,4,4-trimethylpentan-2-yl)-1H-tetrazole) (5a)

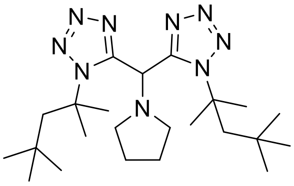

Synthesized according to procedure **D** from 0.5 mmol reaction as white solid, yield: 103 mg (46%); ^{1}H NMR (500 MHz, $CDCl_3$) δ 3.98 (s, 2H), 2.59 – 2.53 (m, 4H), 2.07 (s, 2H), 2.05 (s, 2H), 1.83 (s, 6H), 1.79 – 1.74 (m, 10H), 0.79 (s, 9H), 0.76 (s, 9H). 13C NMR (126 MHz, CDCl3) δ 152.29, 140.58, 65.39, 62.84, 54.21, 53.84, 52.94, 49.78, 31.53, 31.49, 30.67, 30.58, 29.89, 29.62, 23.57.MS (ESI) m/z calculated $[M+Na]^+$: 468.35; found $[M+Na]^+$: 468.46.

N-((1-Benzyl-1H-tetrazol-5-yl)(1-(*tert*-butyl)-1H-tetrazol-5-yl)methyl)-2-phenylethan-1-amine (5b)

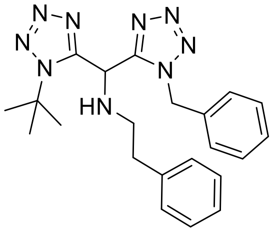

Synthesized according to procedure **D** from 1 mmol reaction as white solid, yield: 104 mg (25%); ^{1}H NMR (500 MHz, $CDCl_3$) δ 7.38 – 7.33 (m, 3H), 7.33 – 7.28 (m, 2H), 7.25 – 7.19 (m, 1H), 7.18 – 7.14 (m, 2H), 7.06 – 7.00 (m, 2H), 5.74 (d, J = 15.1 Hz, 1H), 5.67 (d, J = 15.1 Hz, 1H), 5.16 (d, J = 11.0 Hz, 1H), 2.87 – 2.78 (m, 1H), 2.74 – 2.62 (m, 2H), 2.57 – 2.47 (m, 1H), 2.39 – 2.27 (m, 1H), 1.38 (s, 9H). ^{13}C NMR (126 MHz, $CDCl_3$) δ 152.55,

151.56, 139.33, 133.15, 129.24, 129.08, 128.86, 128.53, 127.94, 126.54, 62.09, 51.91, 50.72, 49.65, 36.16, 29.47. MS (ESI) m/z calculated $[M+Na]^+$: 440.23; found $[M+Na]^+$: 440.34.

***N*-(Bis(1-(*tert*-butyl)-1*H*-tetrazol-5-yl)methyl)butan-1-amine** (**5c**)

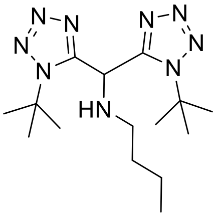

Synthesized according to procedure **D** from 1 mmol reaction as white solid, yield: 60 mg (18%); ^{1}H NMR (500 MHz, $CDCl_3$) δ 5.58 (d, *J* = 12.4 Hz, 1H), 2.67 – 2.59 (m, 2H), 2.52 (dt, *J* = 13.3, 6.9 Hz, 1H), 1.74 (s, 18H), 1.58 – 1.49 (m, 2H), 1.44 – 1.32 (m, 2H), 0.91 (t, *J* = 7.3 Hz, 3H). ^{13}C NMR (126 MHz, $CDCl_3$) δ 152.66, 62.52, 52.44, 48.39, 32.04, 29.56, 28.69, 20.24, 13.83. MS (ESI) m/z calculated $[M+Na]^+$: 358.24; found $[M+Na]^+$: 358.37.

1*H*-Tetrazole-5-carbaldehyde (**6**)

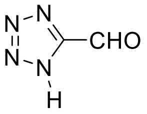

Synthesized according to procedure **E** from 1 mmol reaction as colorless liquid, yield: 30 mg (31%); ^{1}H NMR (500 MHz, $CDCl_3$) δ 9.19 (s, 1H).

^{1}H NMR and ^{13}C NMR spectra

(1-Benzyl-1*H*-tetrazol-5-yl)methanol (1a)

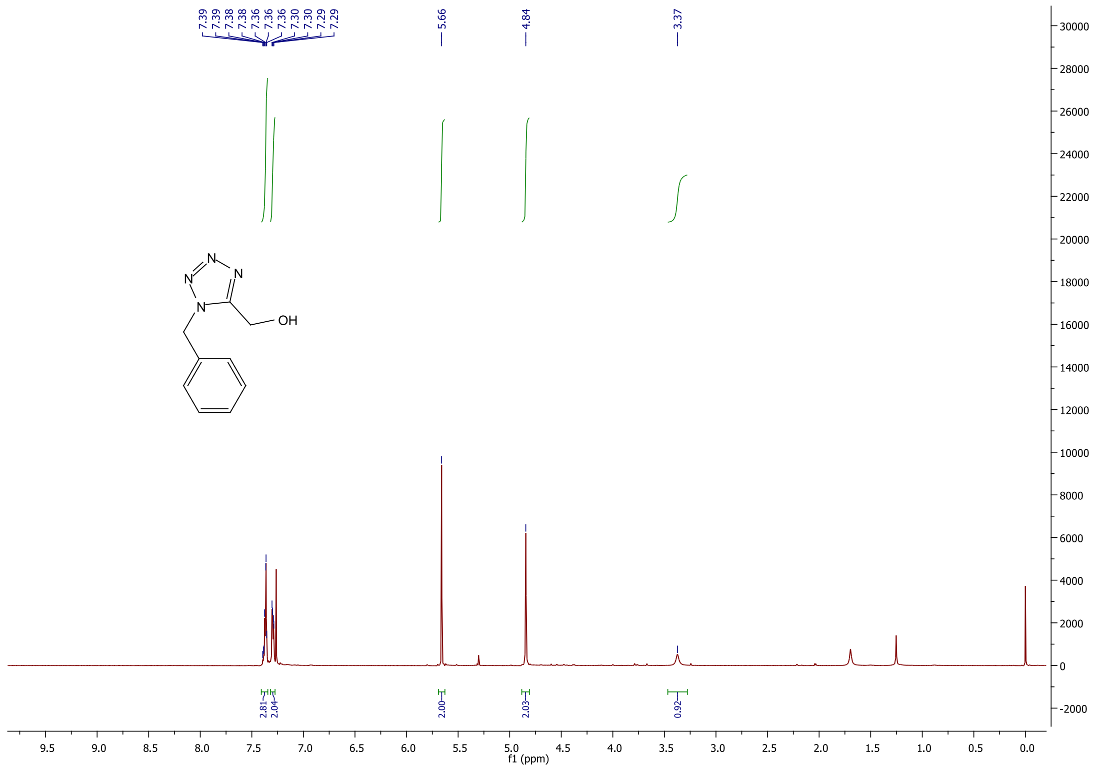

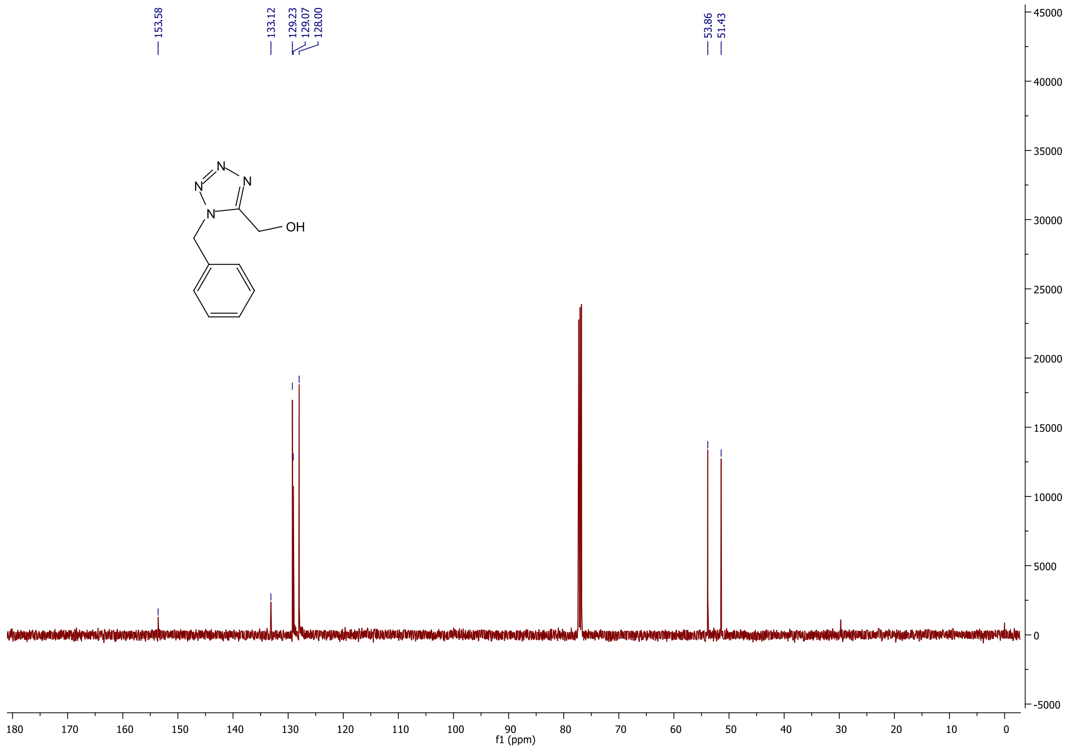

(1-(2,4,4-Trimethylpentan-2-yl)-1*H*-tetrazol-5-yl)methanol (1b)

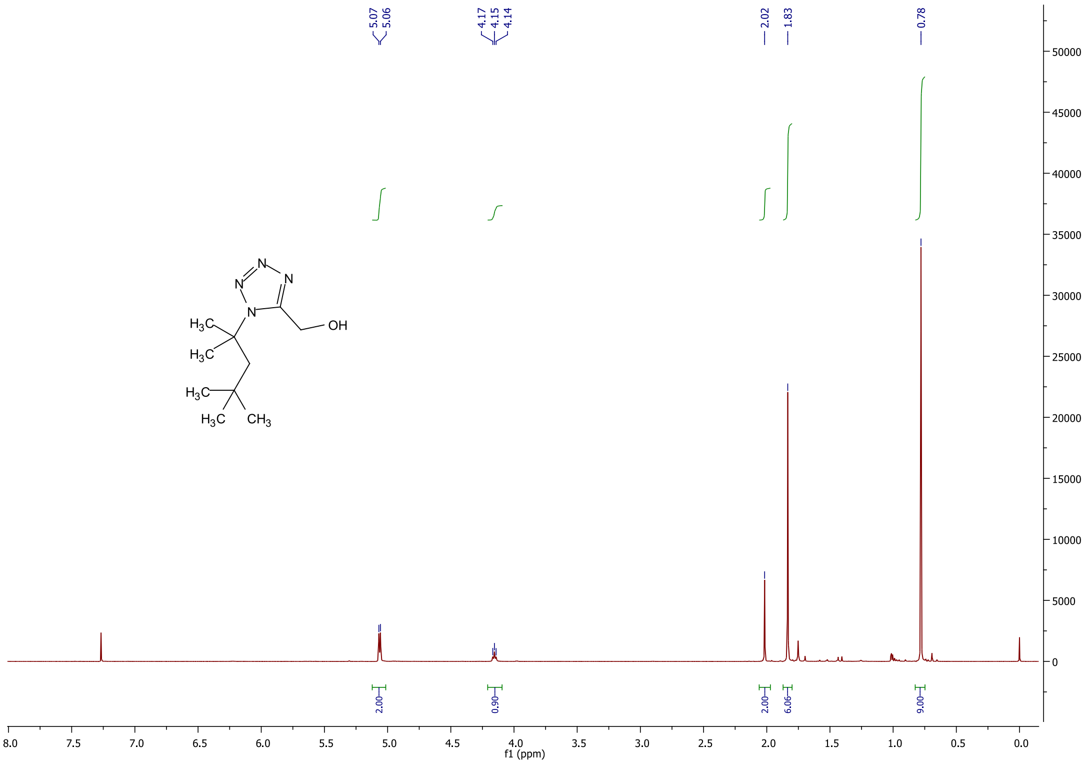

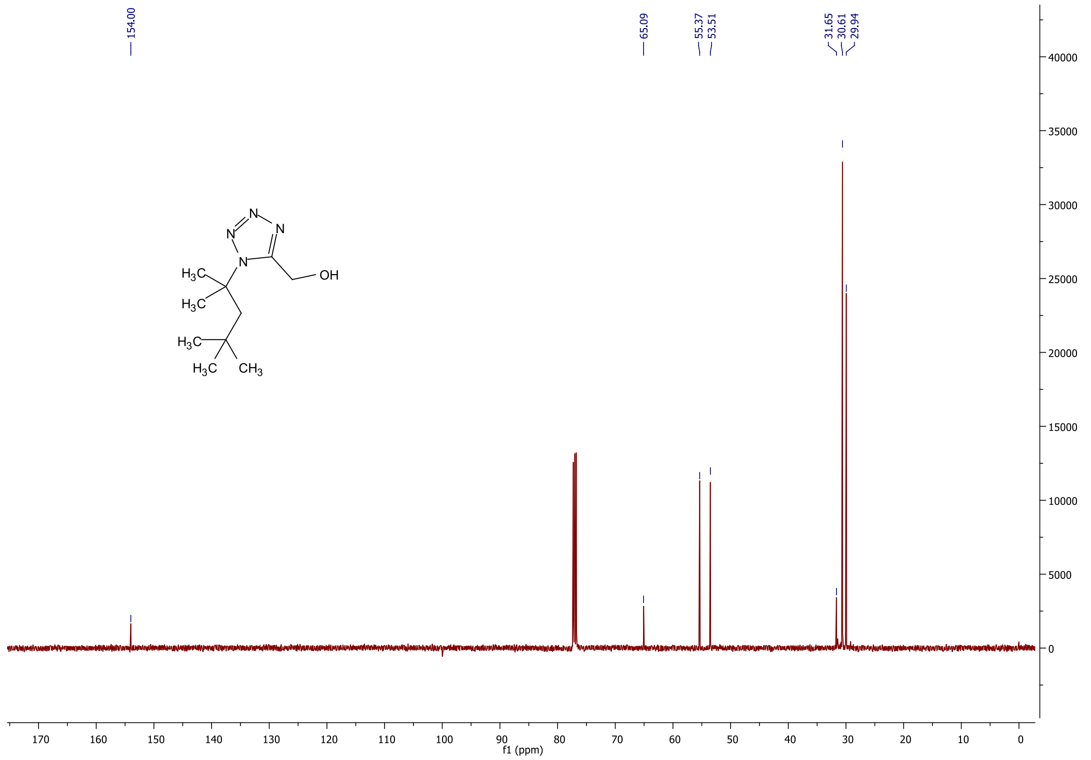

(1-(*tert*-Butyl)-1*H*-tetrazol-5-yl)methanol (1c)

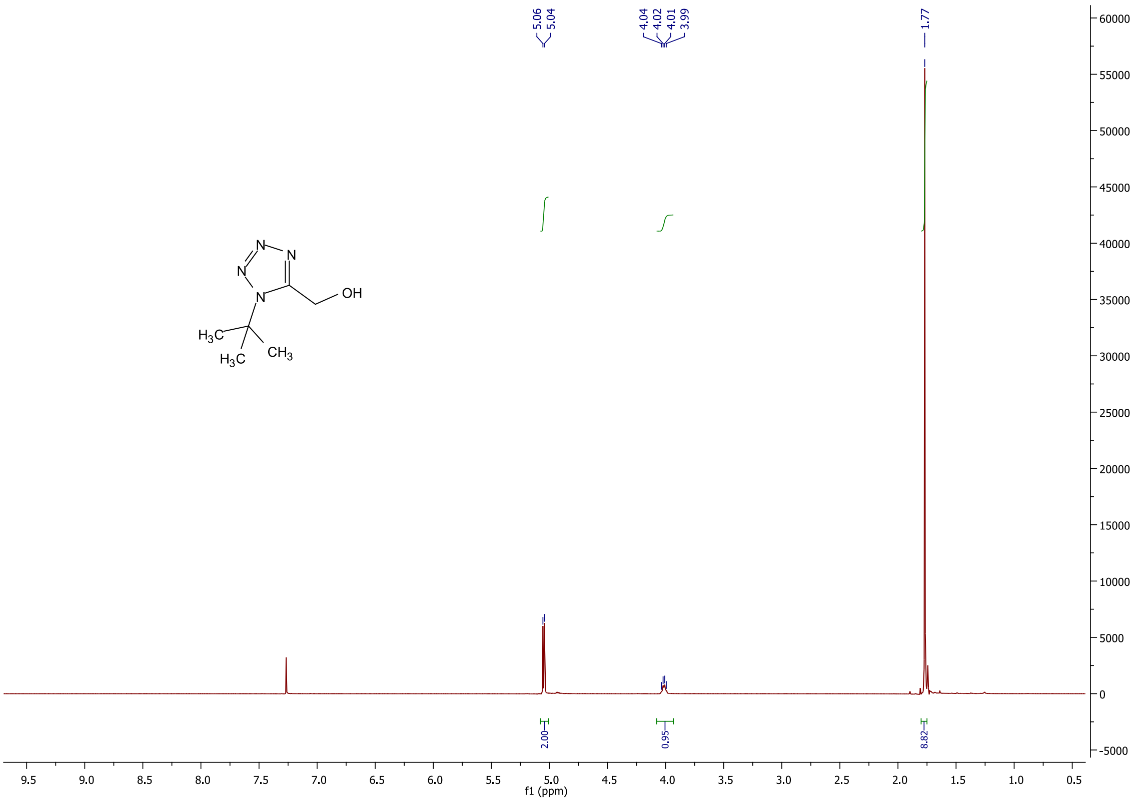

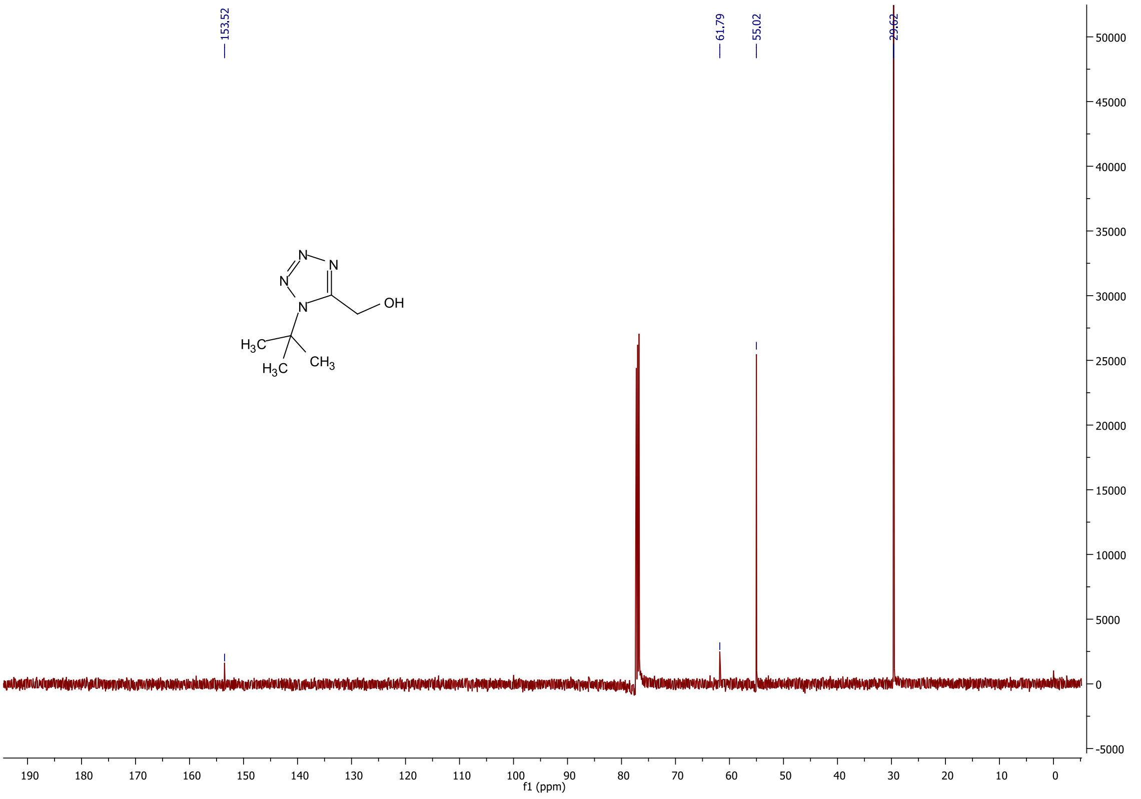

3-(5-(Hydroxymethyl)-1*H*-tetrazol-1-yl)propanenitrile (1d)

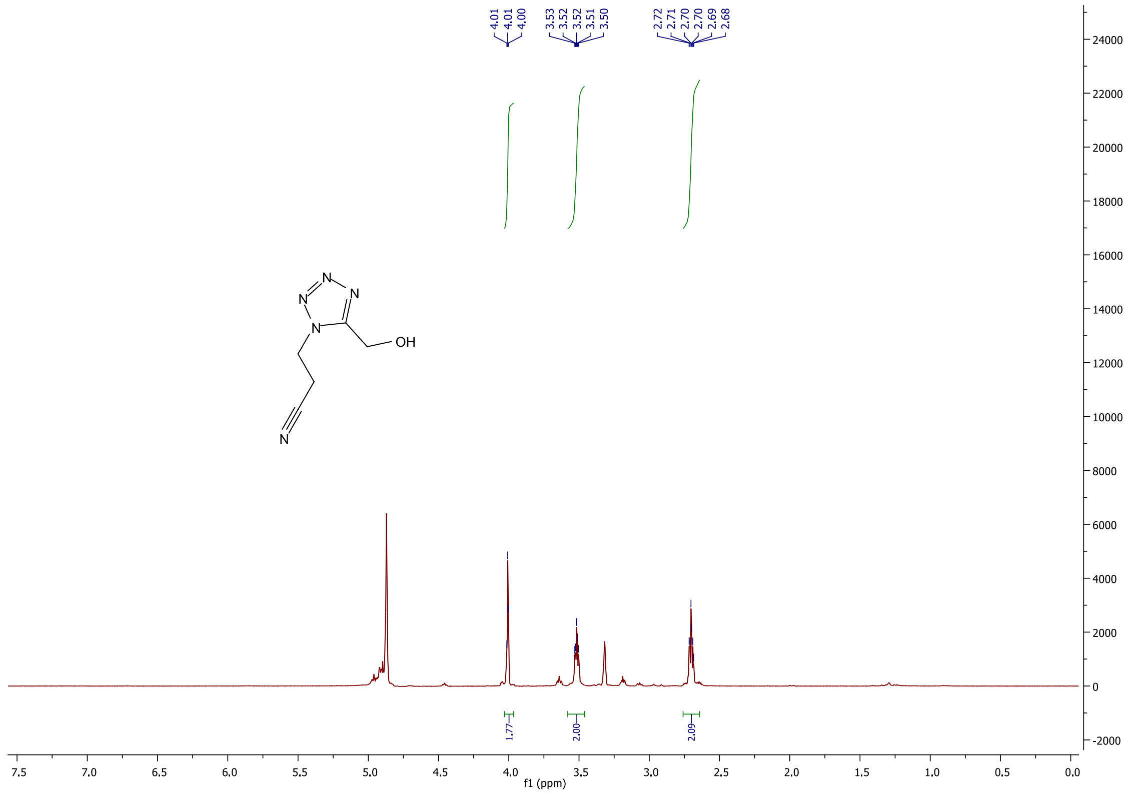

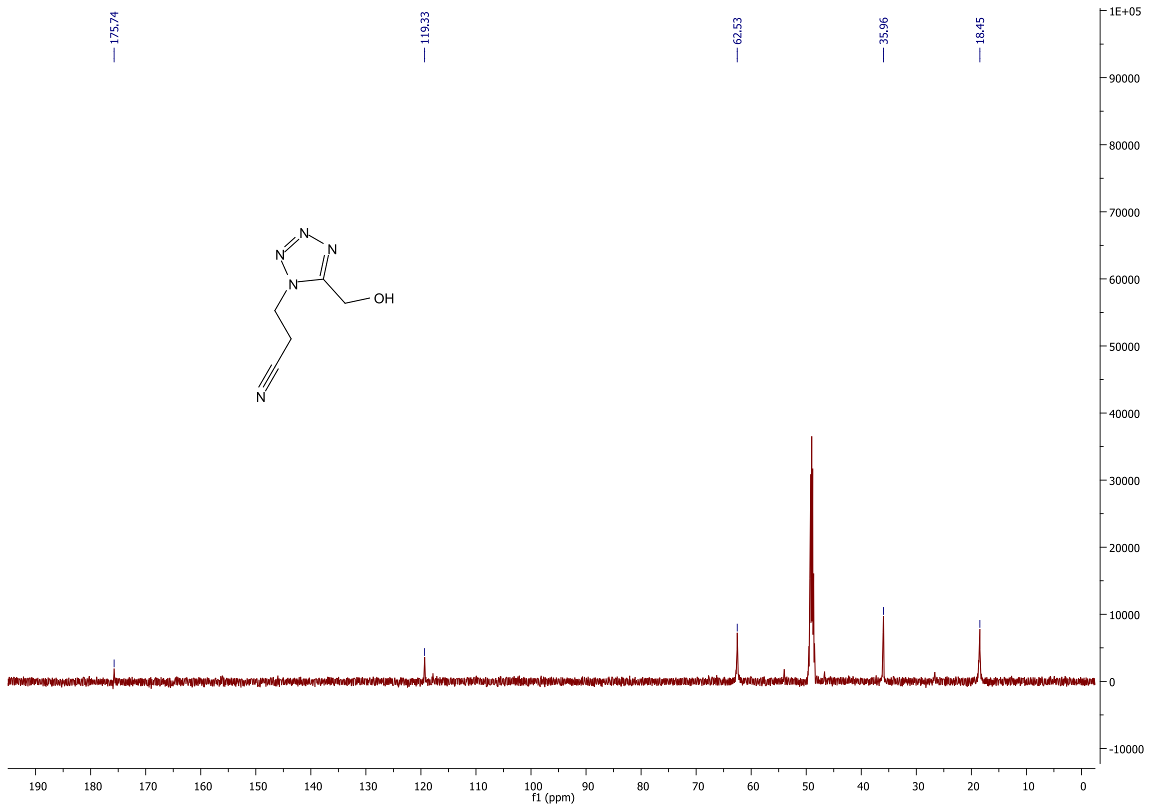

1-Benzyl-1*H*-tetrazole-5-carbaldehyde (2a)

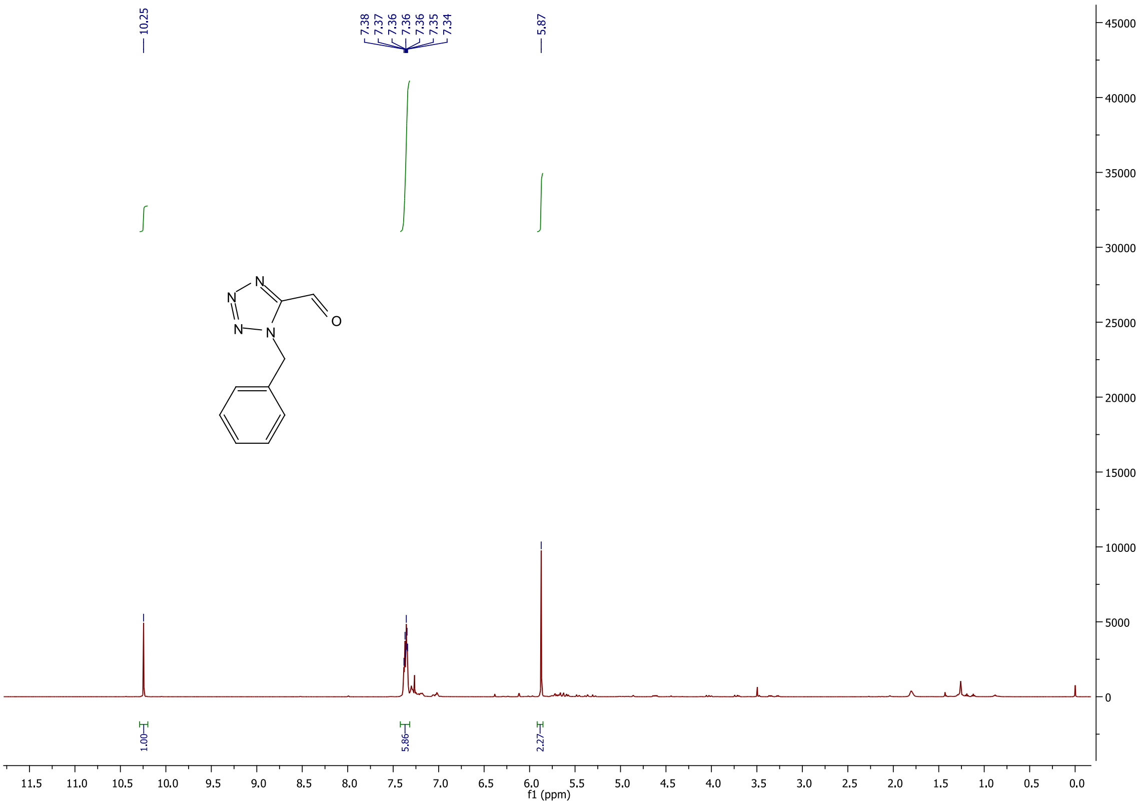

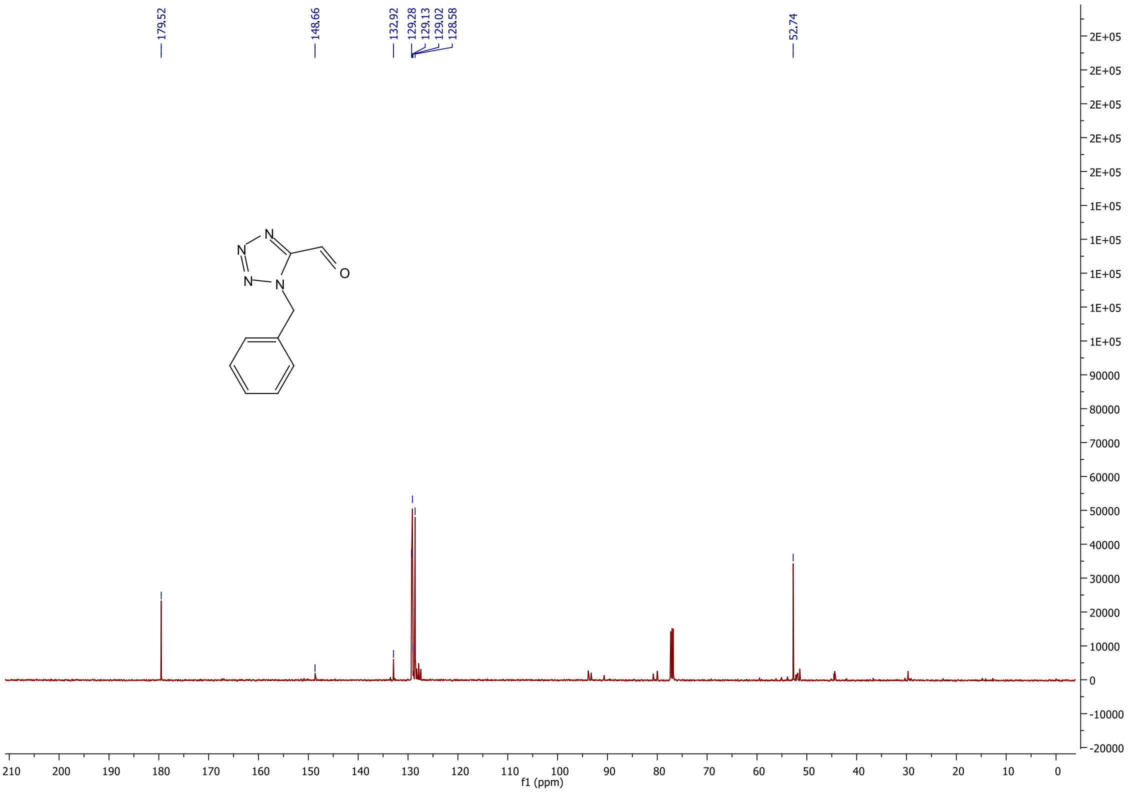

1-(2,4,4-Trimethylpentan-2-yl)-1*H*-tetrazole-5-carbaldehyde (2b)

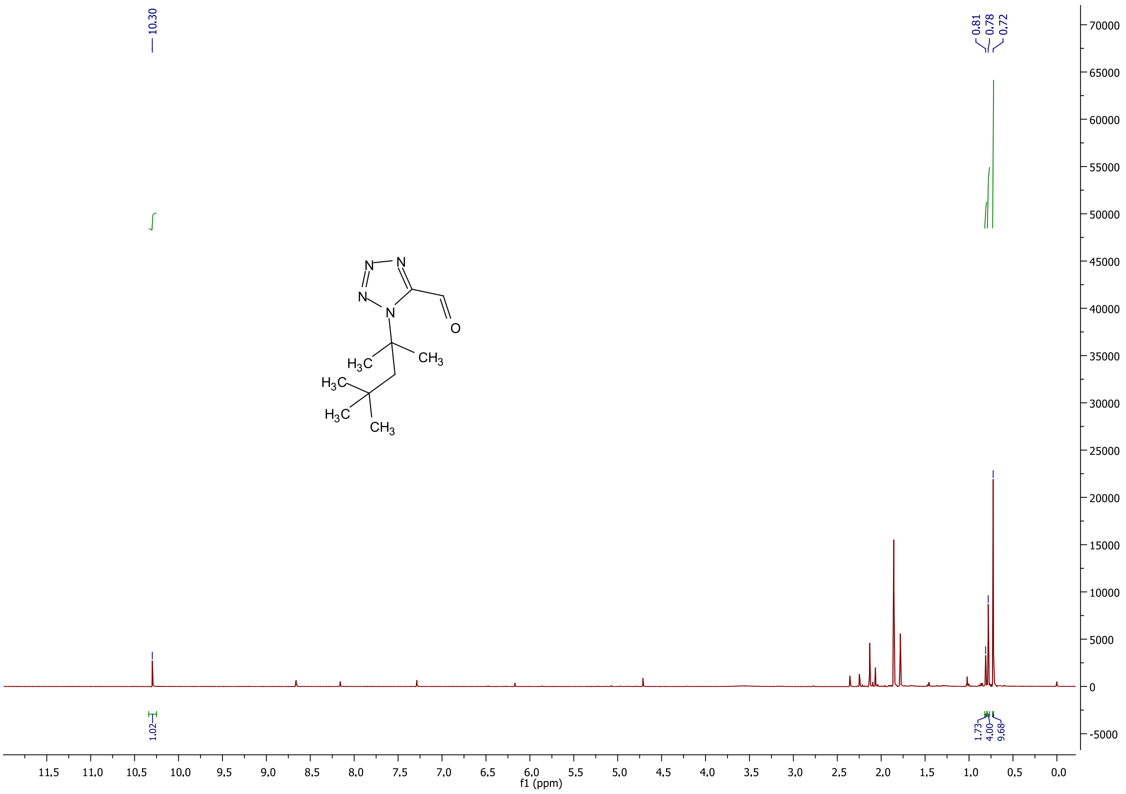

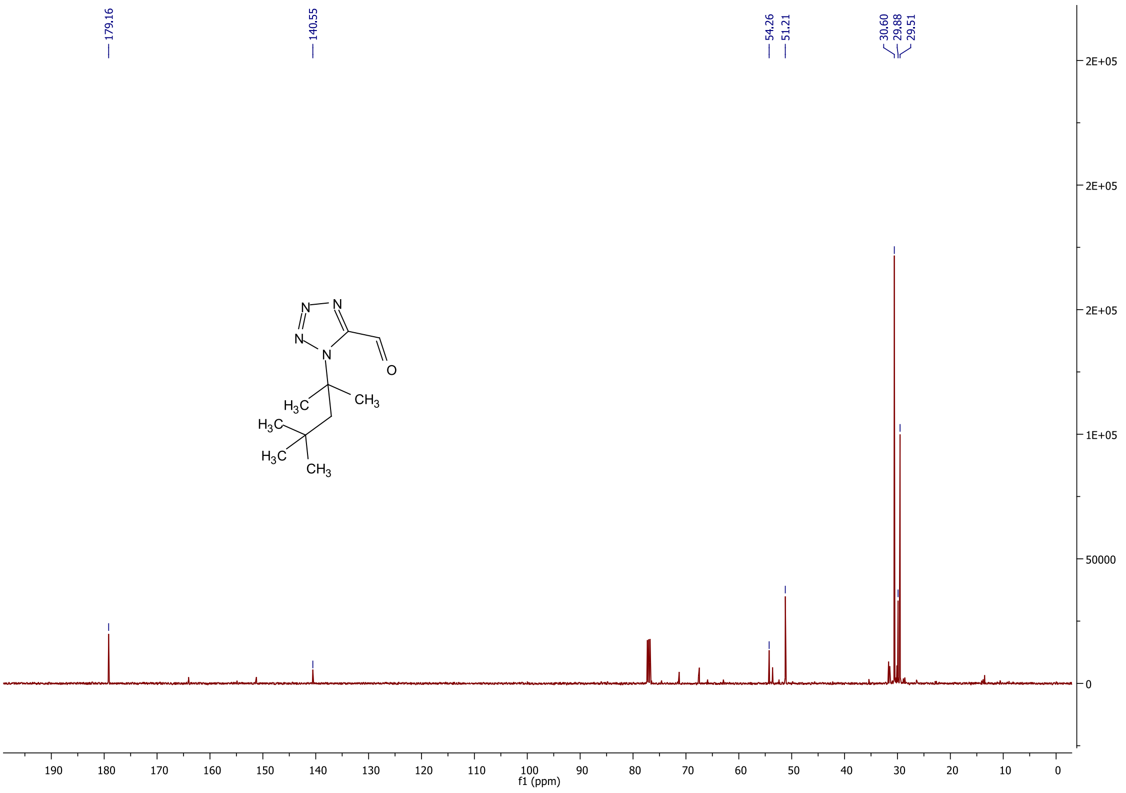

1-(*tert*-Butyl)-1*H*-tetrazole-5-carbaldehyde (2c)

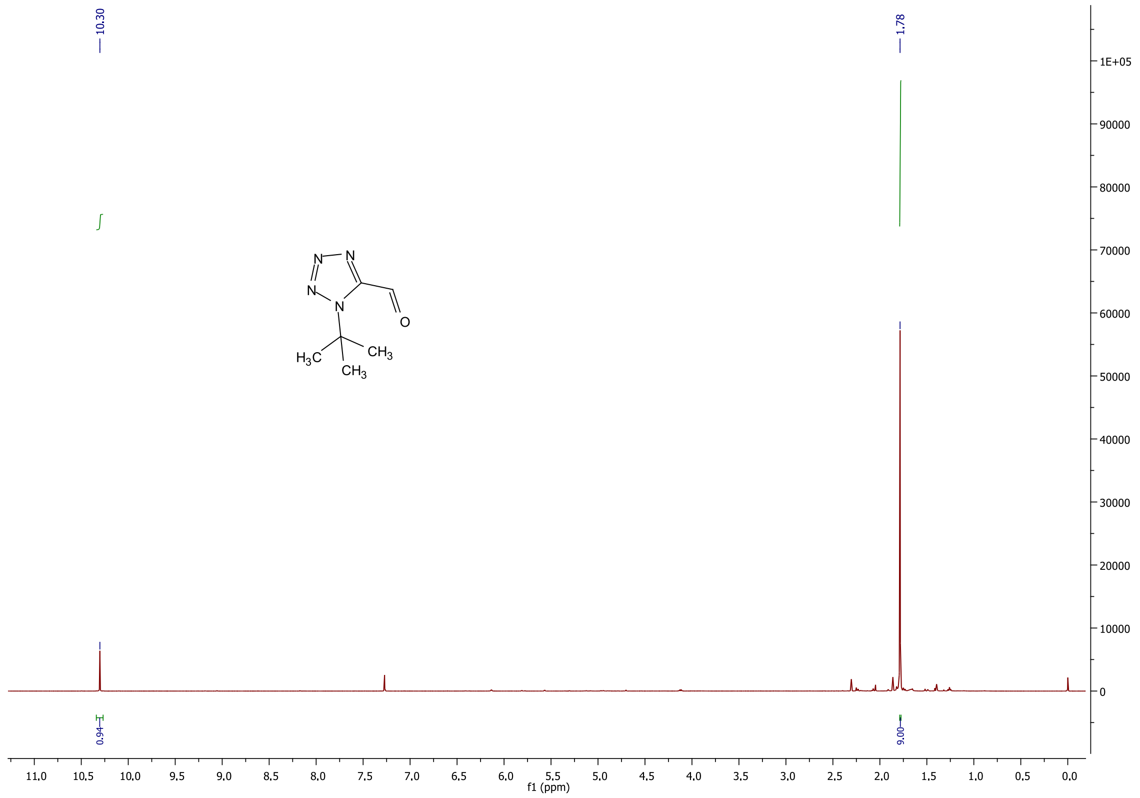

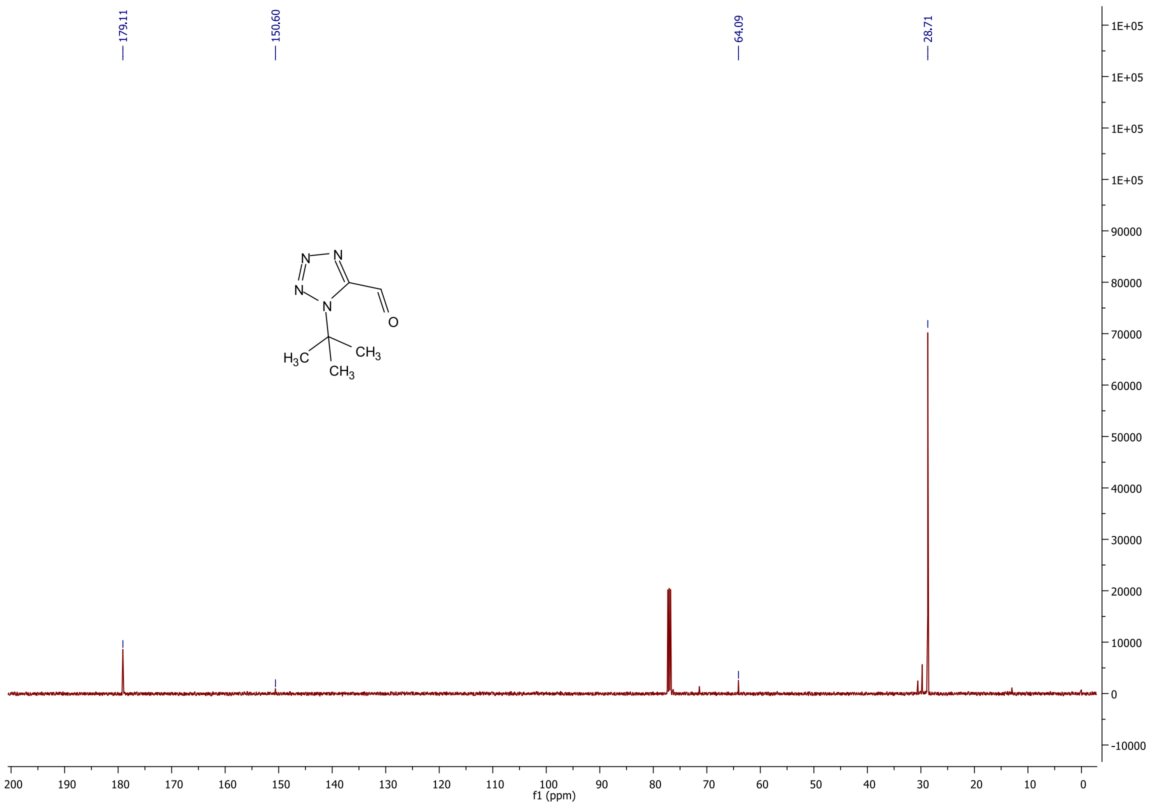

3-(5-Formyl-1*H*-tetrazol-1-yl)propanenitrile (2d)

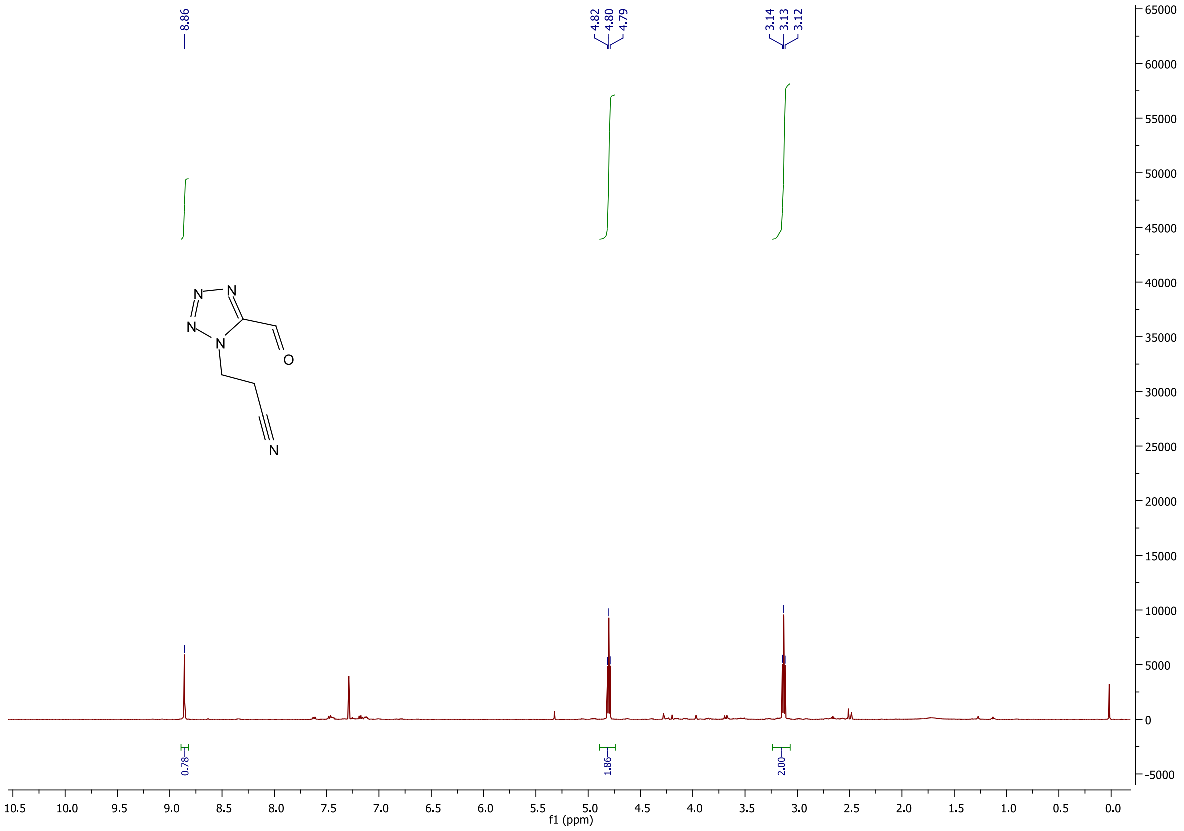

1-(1-Benzyl-1*H*-tetrazol-5-yl)-2-((2-cyanoethyl)amino)-2-oxoethyl benzoate (3a)

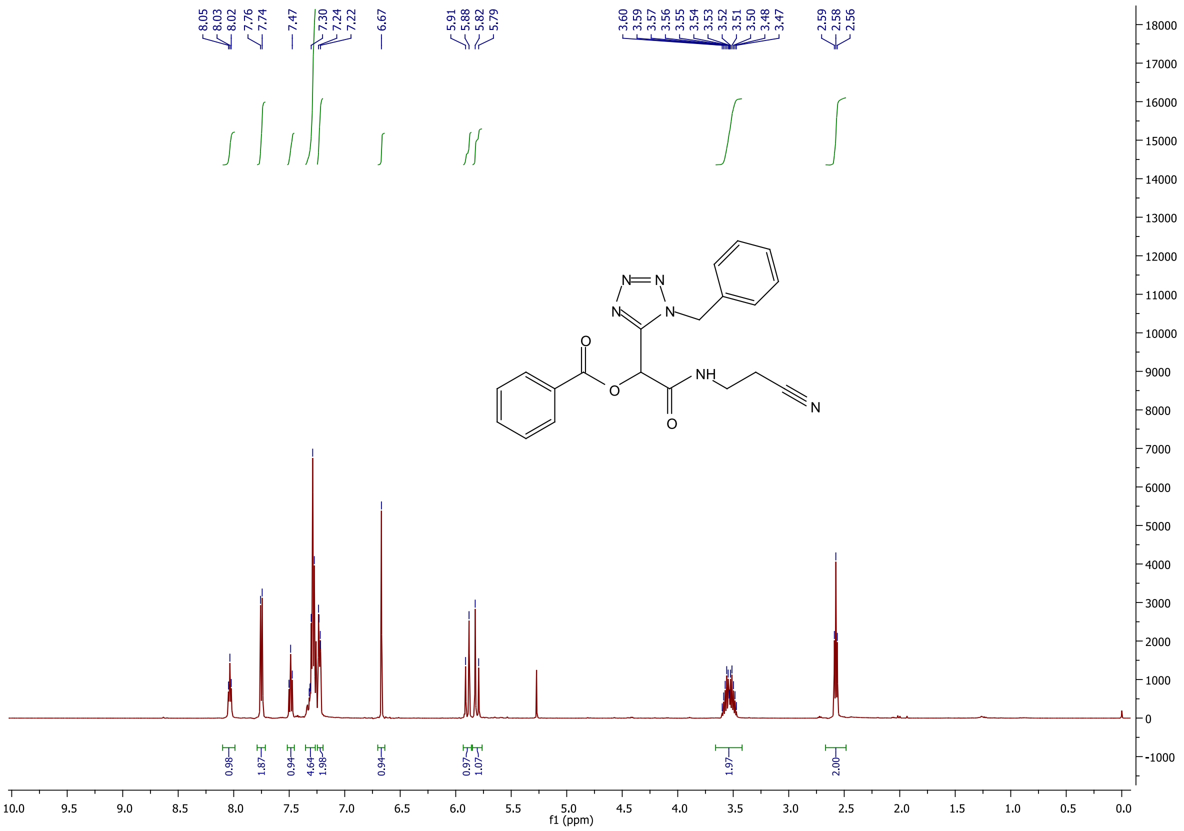

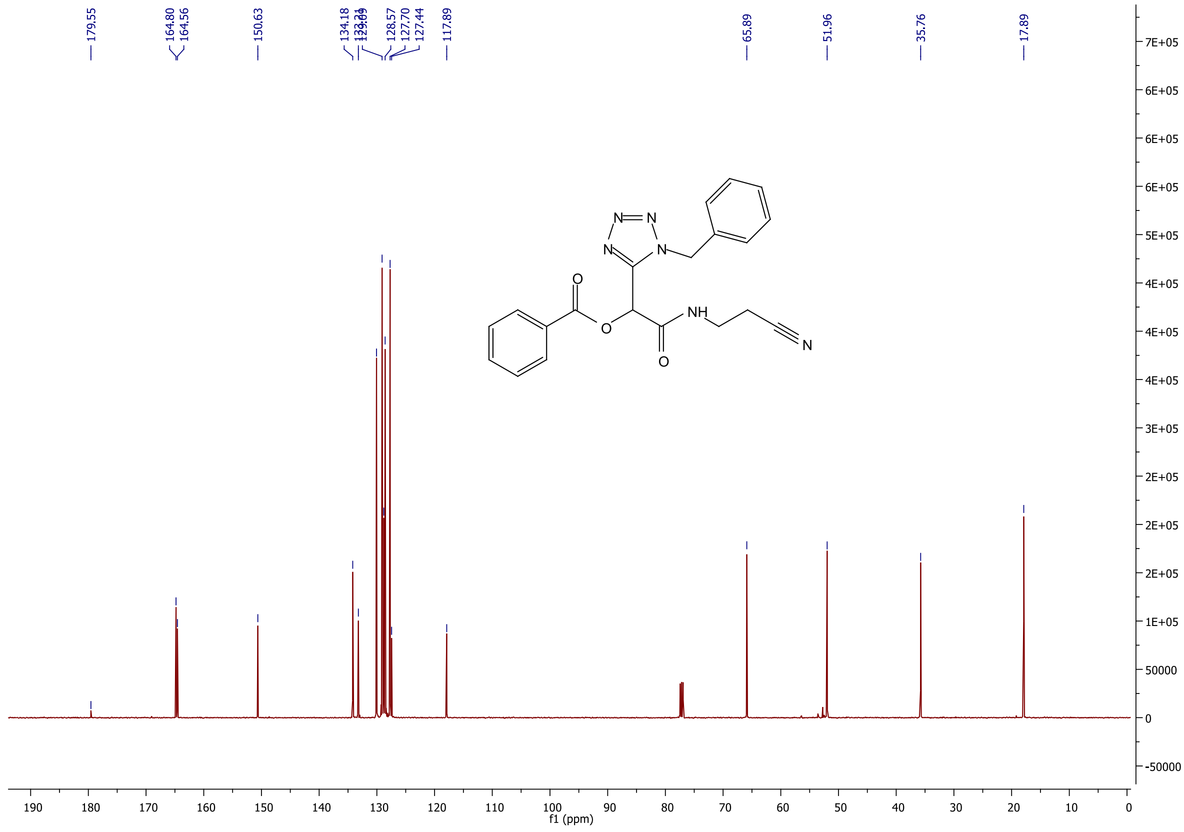

1-(1-Benzyl-1*H*-tetrazol-5-yl)-2-oxo-2-(phenethylamino)ethyl acetate (3b)

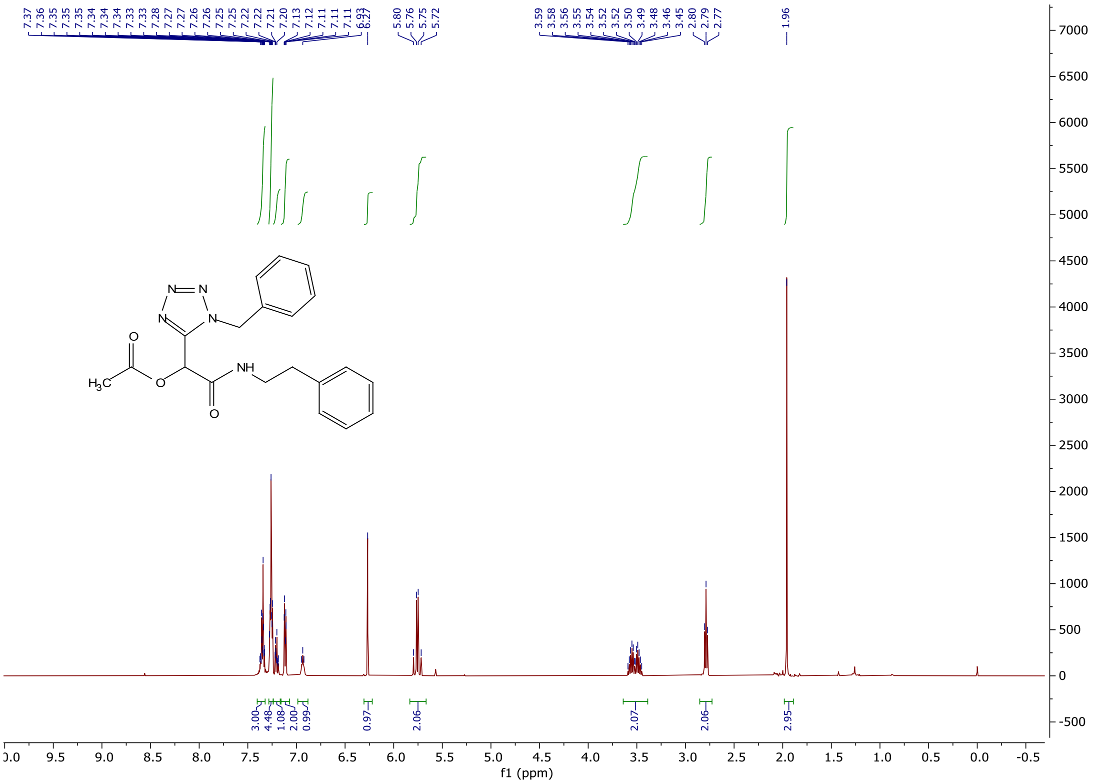

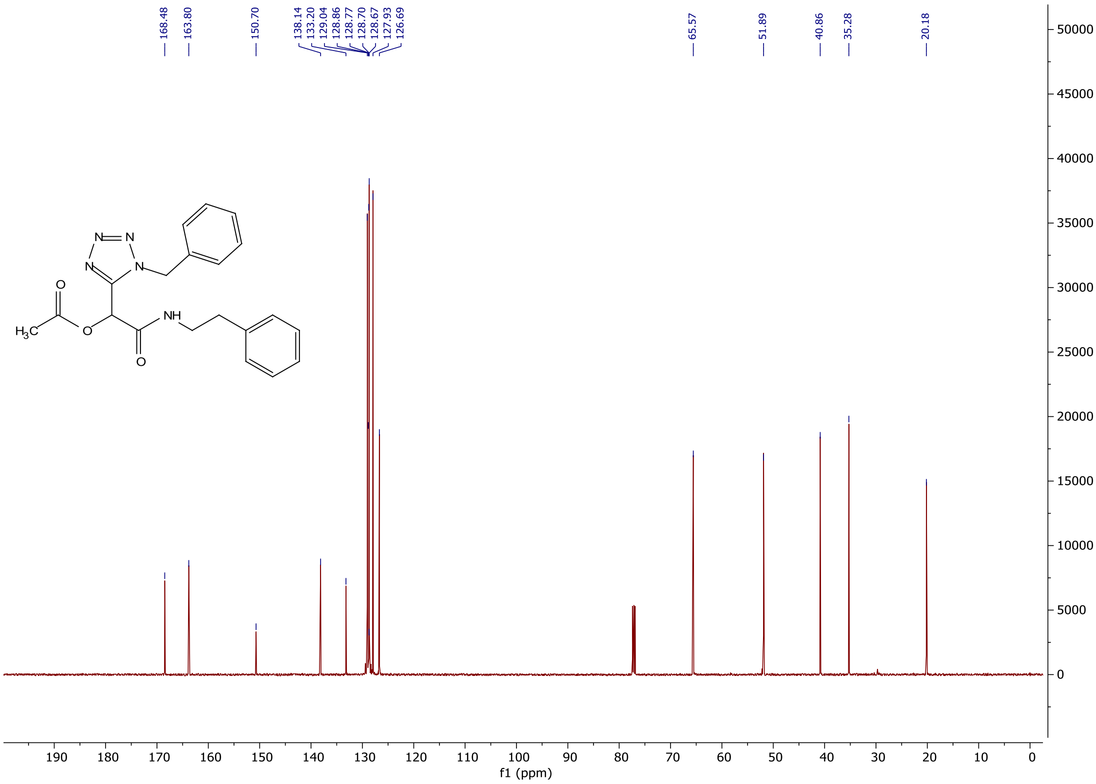

1-(1-Benzyl-1*H*-tetrazol-5-yl)-2-oxo-2-((2,4,4-trimethylpentan-2-yl)amino)ethyl benzoate (3c)

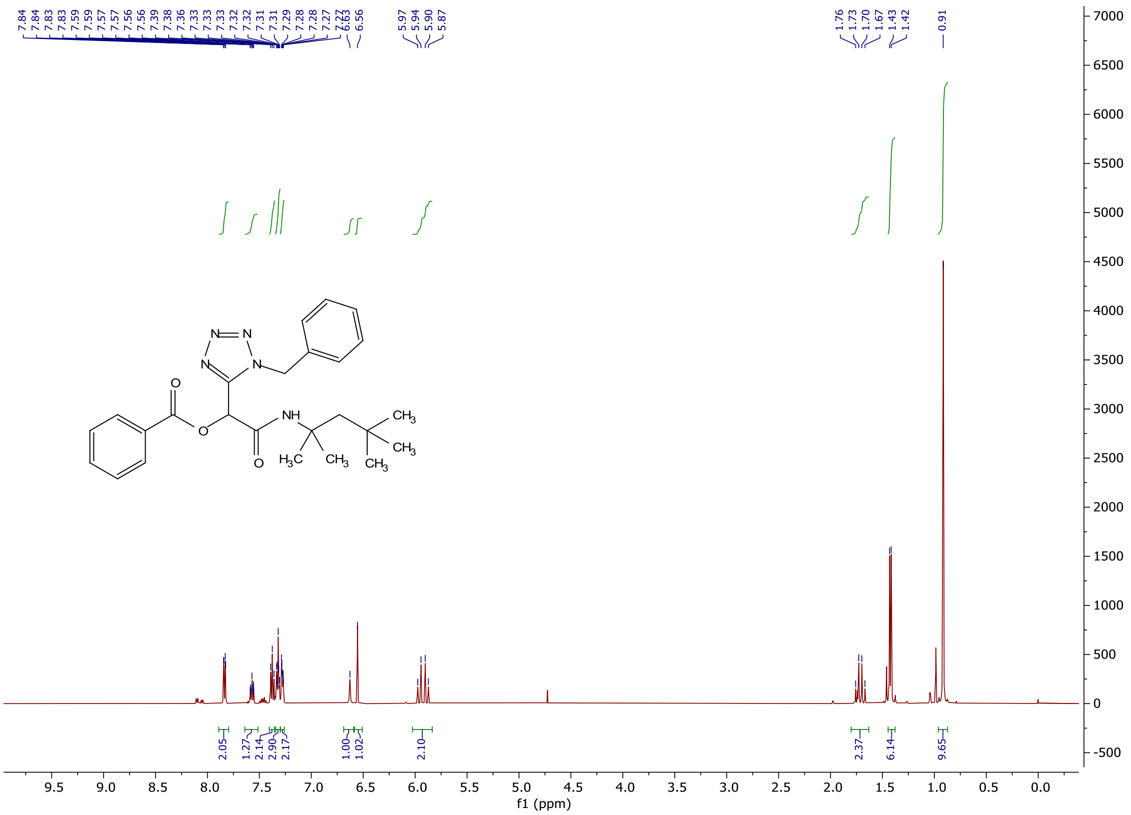

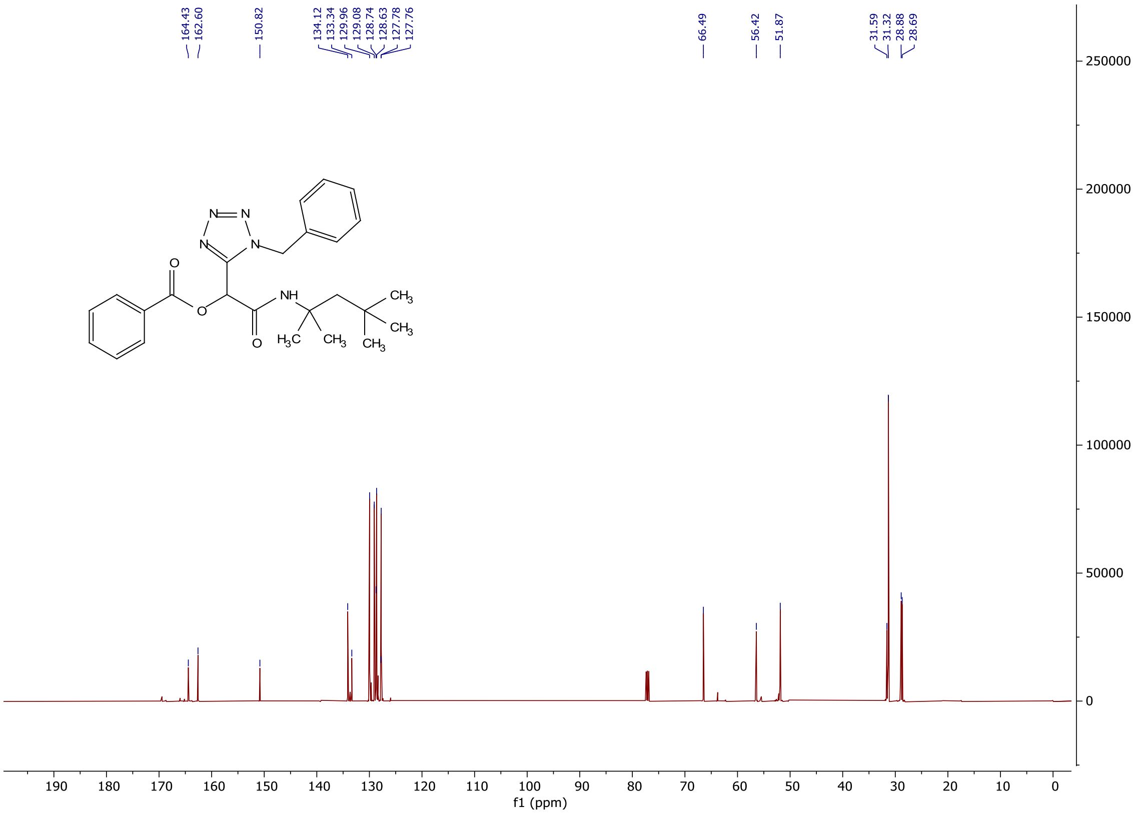

2-(*tert*-Butylamino)-2-oxo-1-(1-(2,4,4-trimethylpentan-2-yl)-1*H*-tetrazol-5-yl)ethyl 2-phenylacetate (3d)

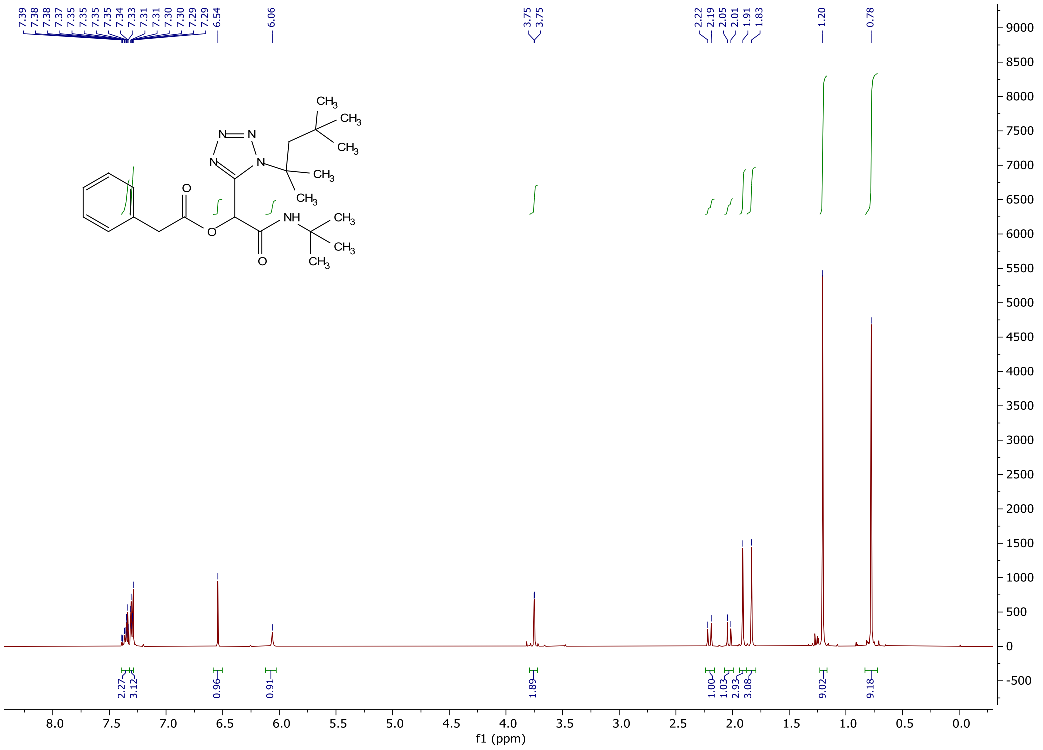

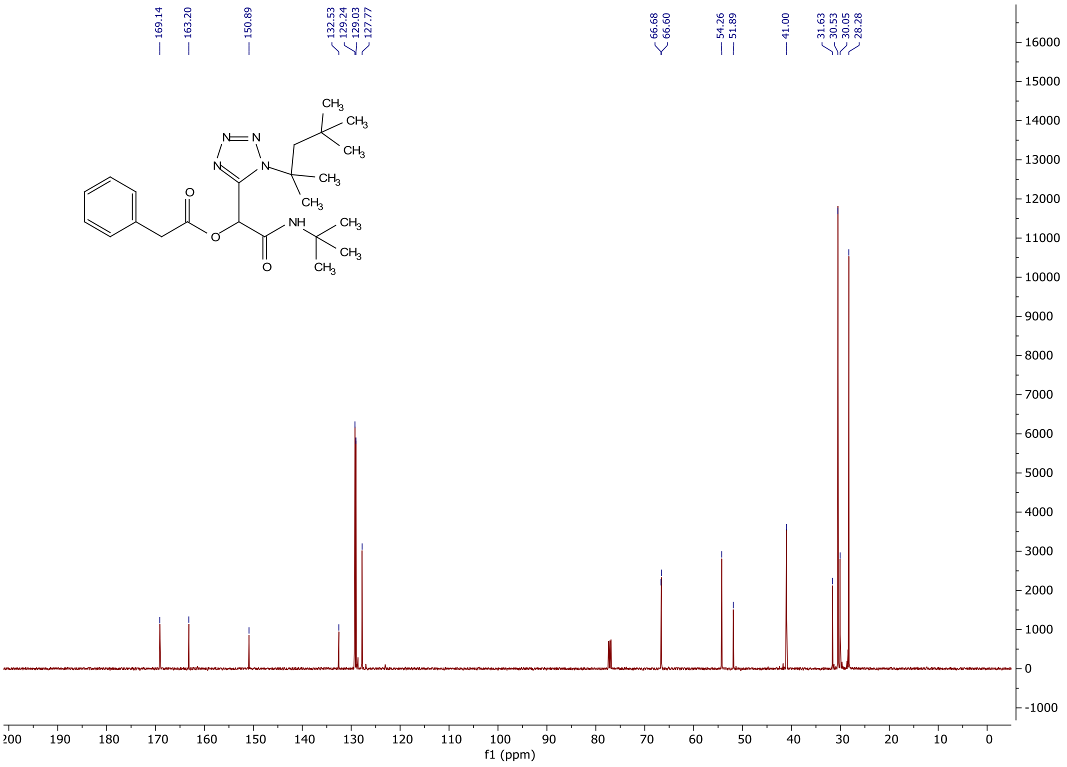

2-Oxo-2-(phenethylamino)-1-(1-(2,4,4-trimethylpentan-2-yl)-1*H*-tetrazol-5-yl)ethyl acetate (3e)

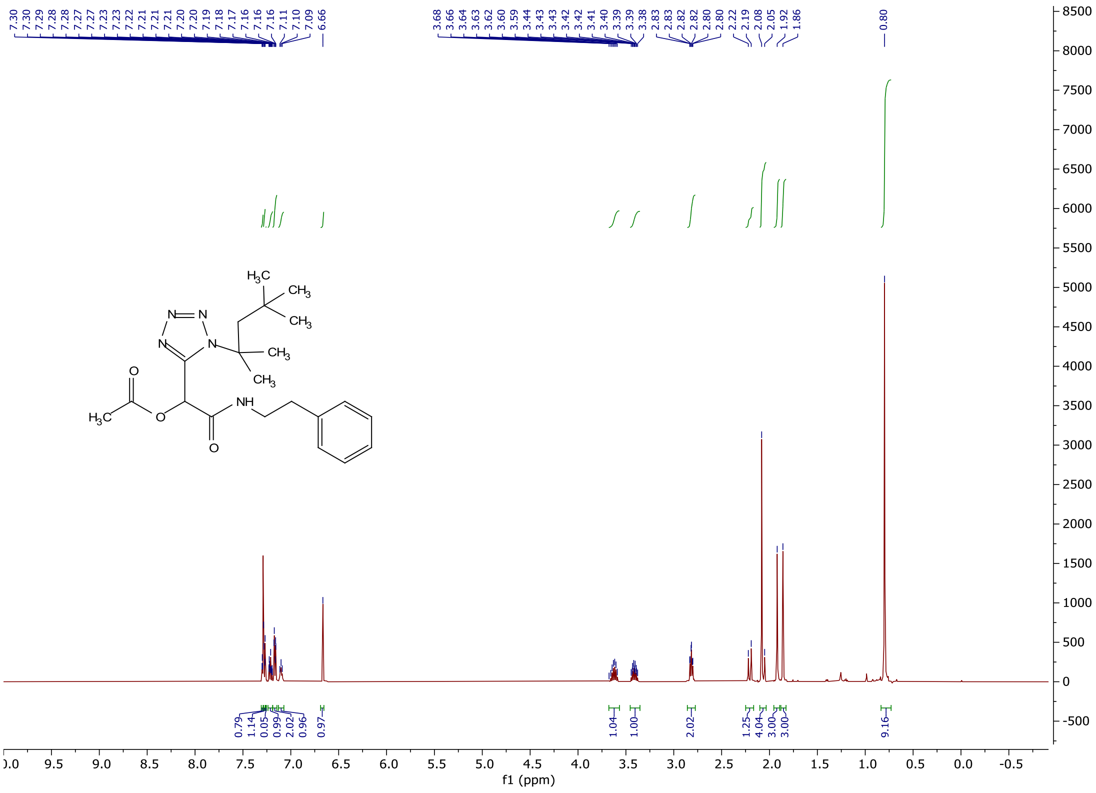

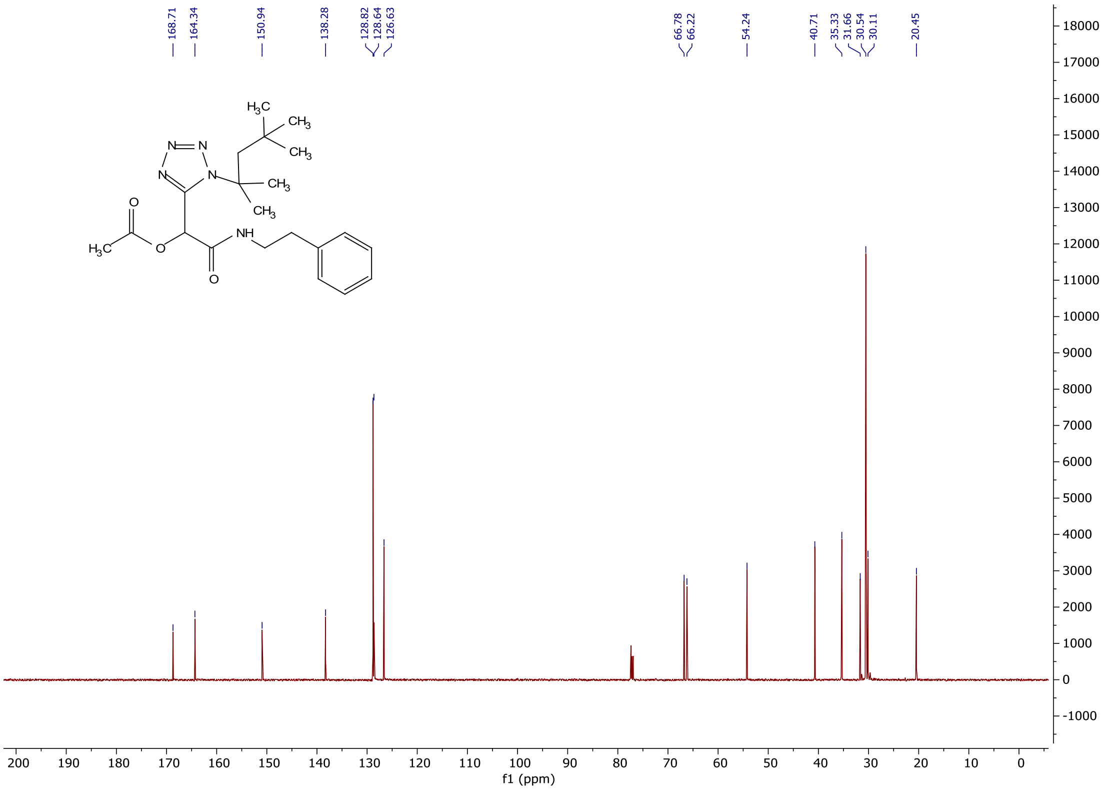

2-(*tert*-Butylamino)-2-oxo-1-(1-(2,4,4-trimethylpentan-2-yl)-1*H*-tetrazol-5-yl)ethyl benzoate (3f)

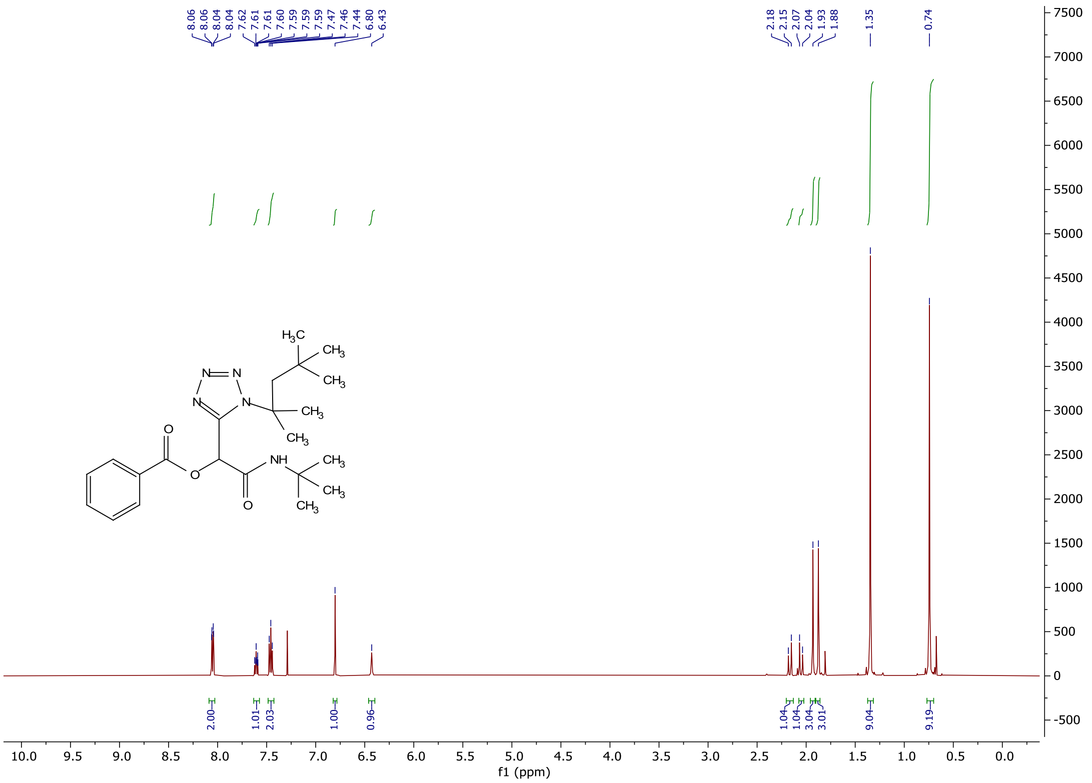

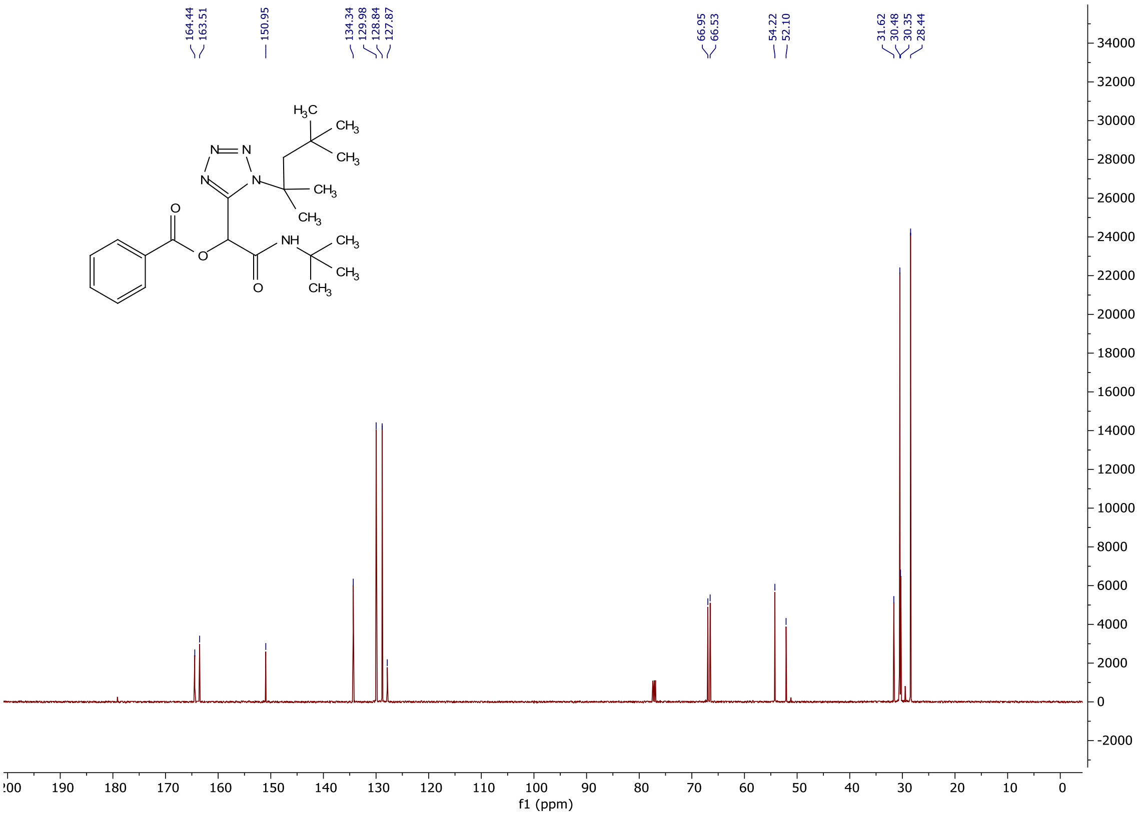

2-((2-Isocyanoethyl)amino)-2-oxo-1-(1-(2,4,4-trimethylpentan-2-yl)-1*H*-tetrazol-5-yl)ethyl benzoate (3g)

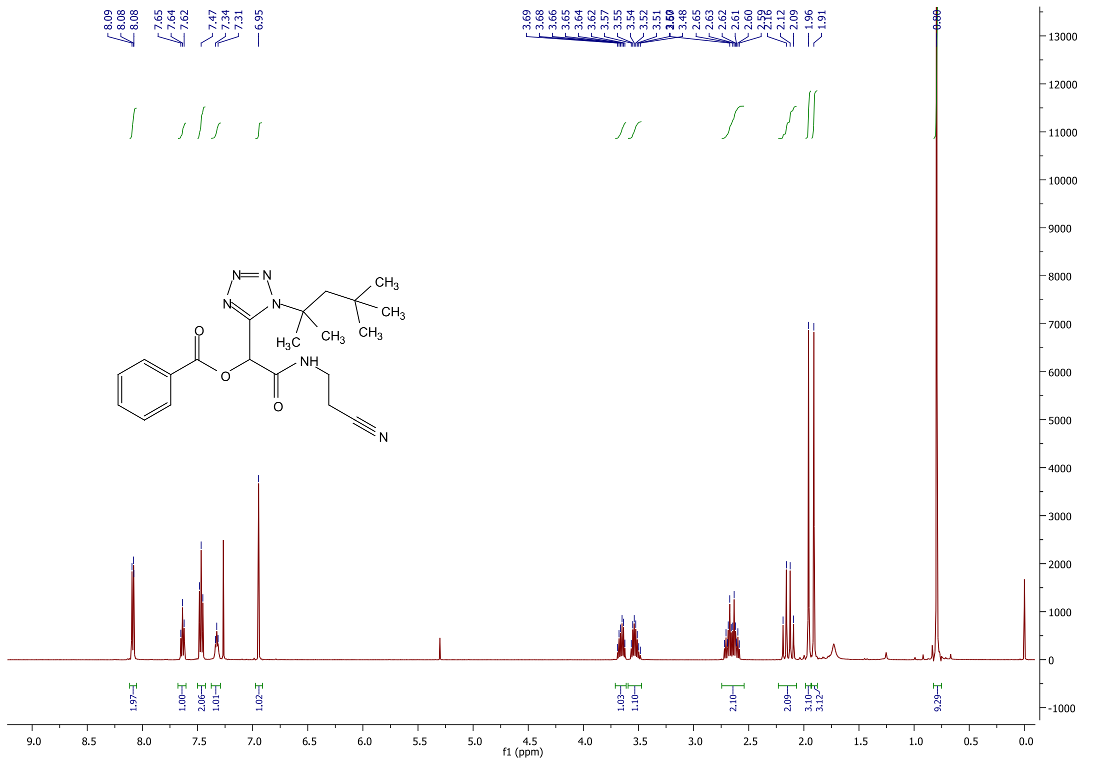

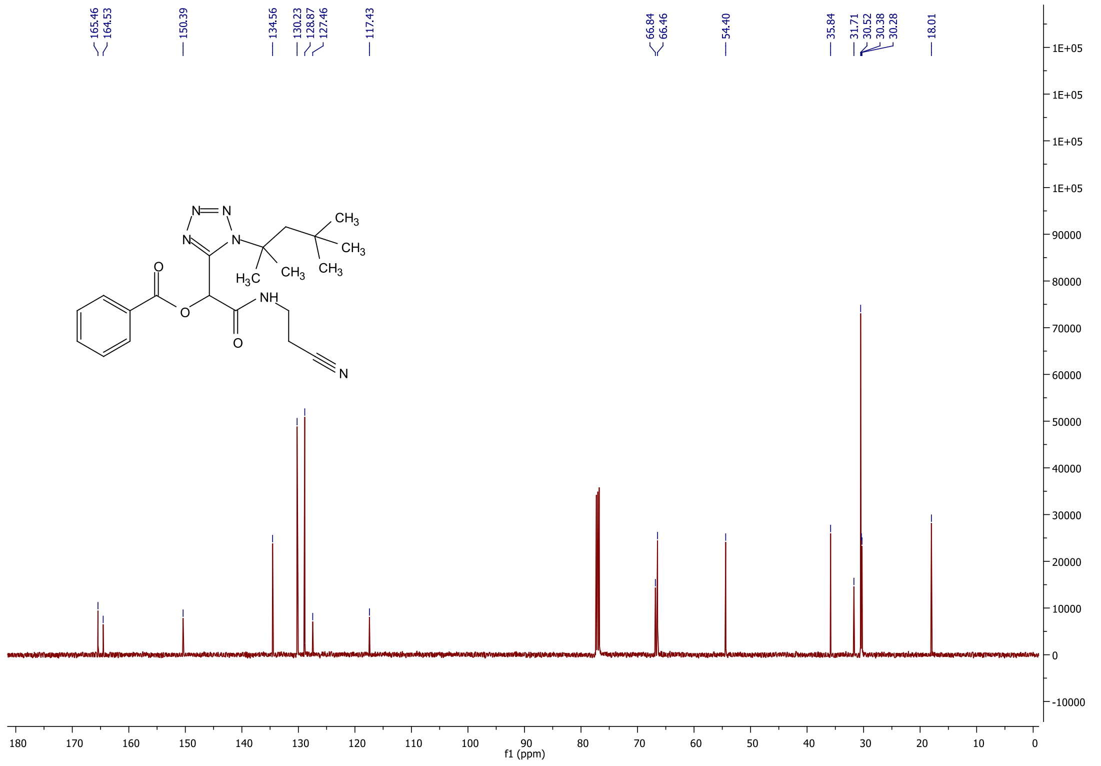

2-(Benzylamino)-2-oxo-1-(1-(2,4,4-trimethylpentan-2-yl)-1*H*-tetrazol-5-yl)ethyl picolinate (3h)

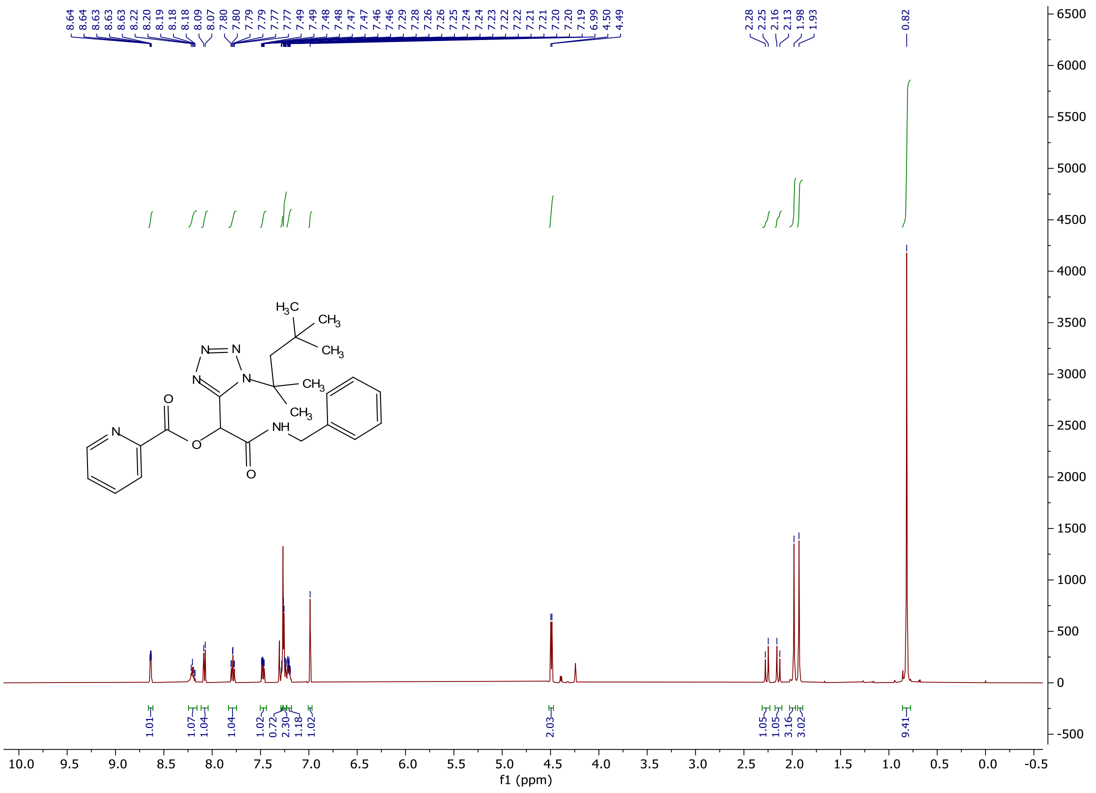

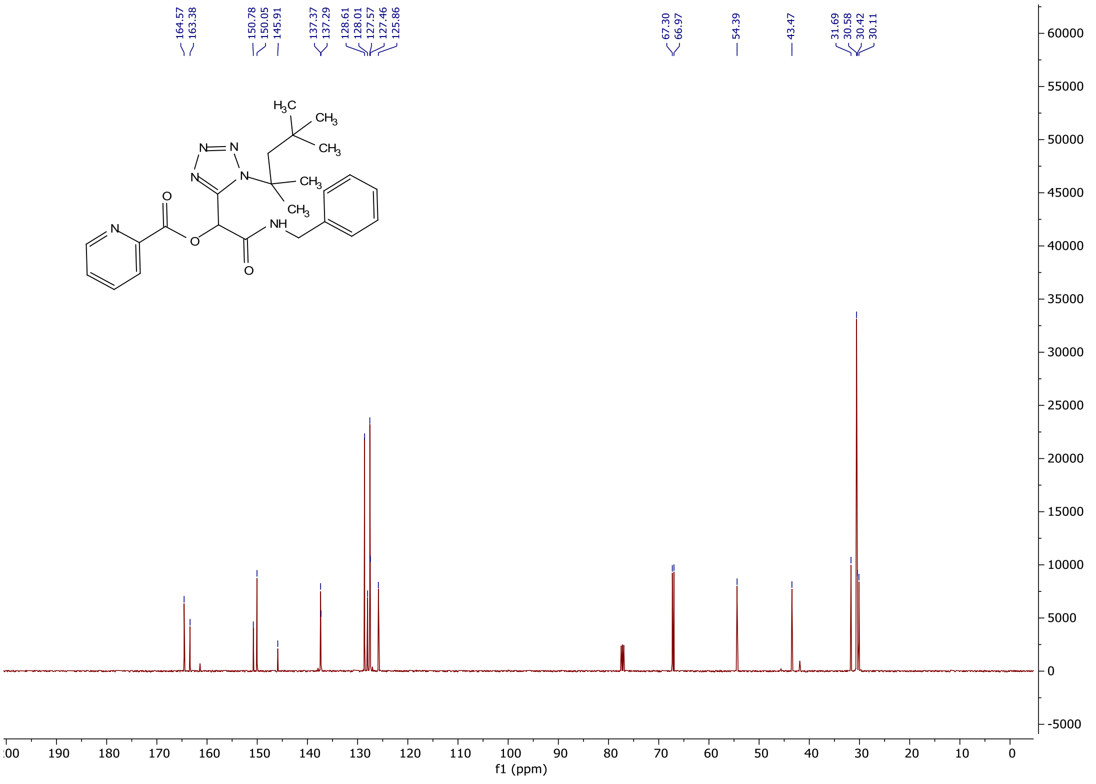

2-(Benzylamino)-1-(1-(*tert*-butyl)-1*H*-tetrazol-5-yl)-2-oxoethyl 2-phenylacetate (3i)

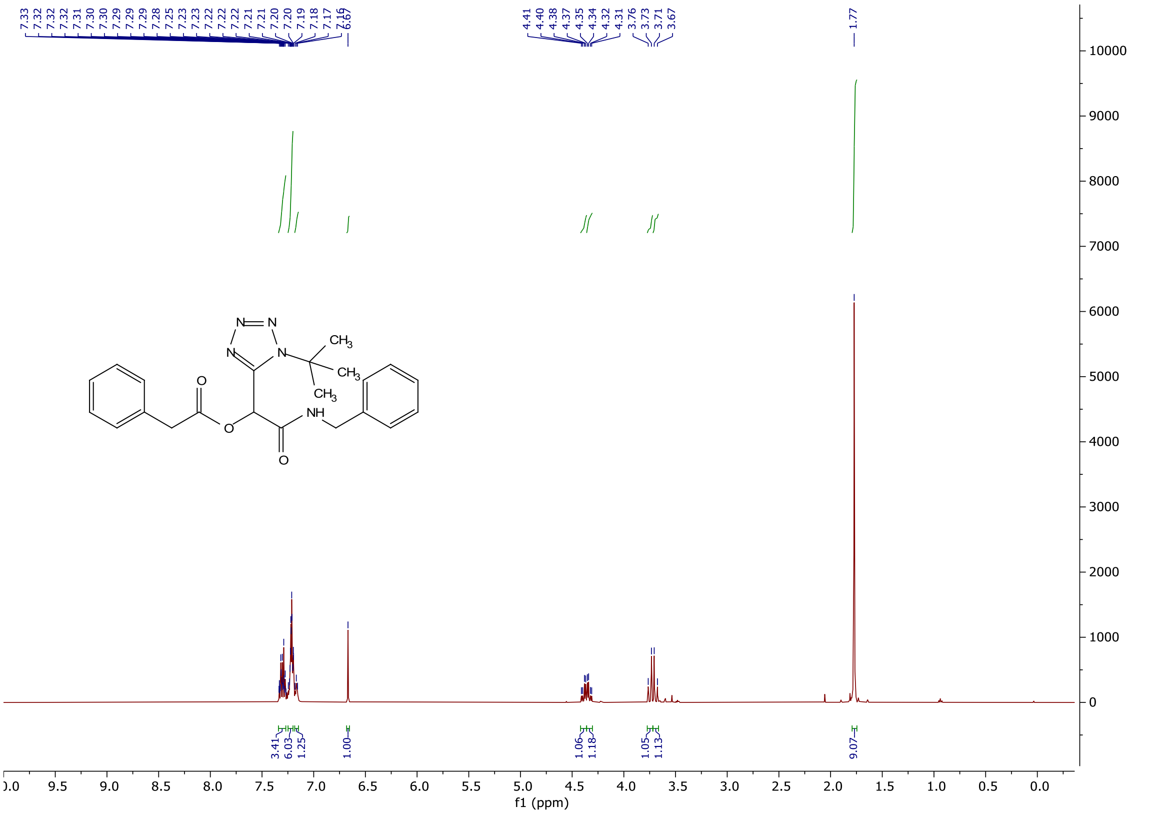

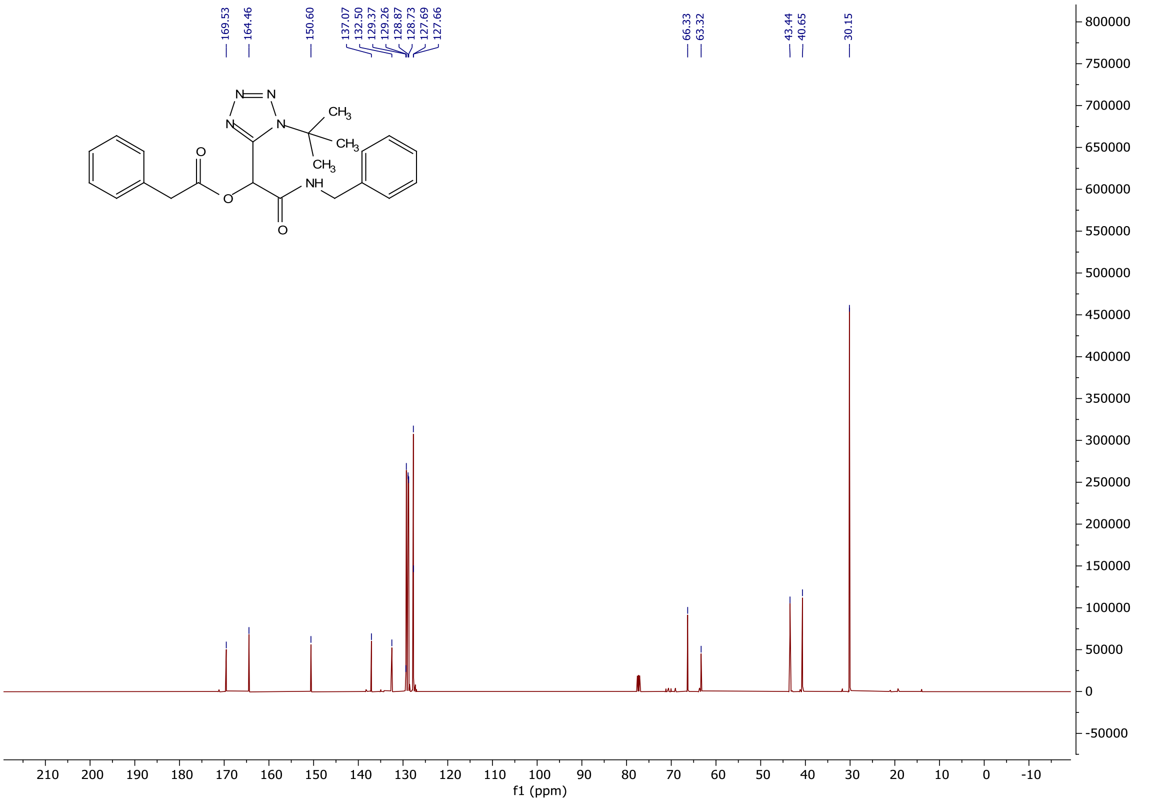

1-(1-(*tert*-Butyl)-1*H*-tetrazol-5-yl)-2-oxo-2-(phenethylamino)ethyl acetate (3j)

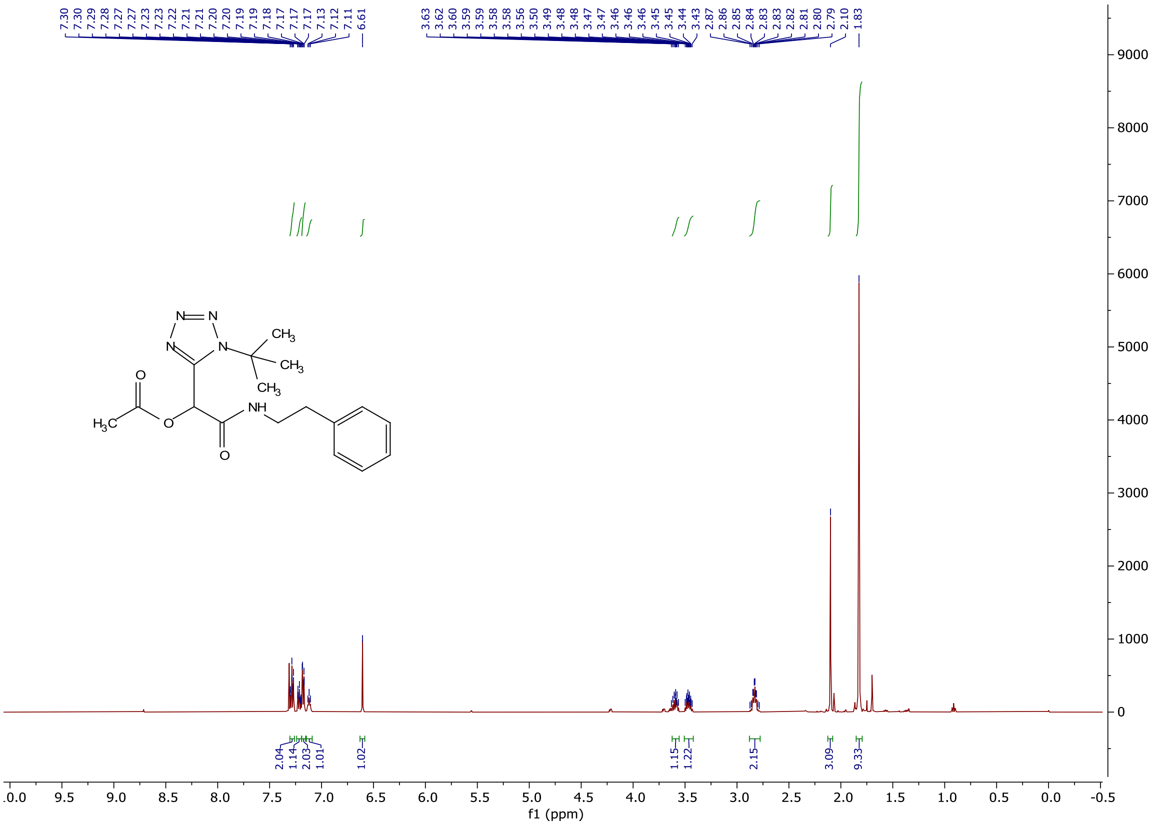

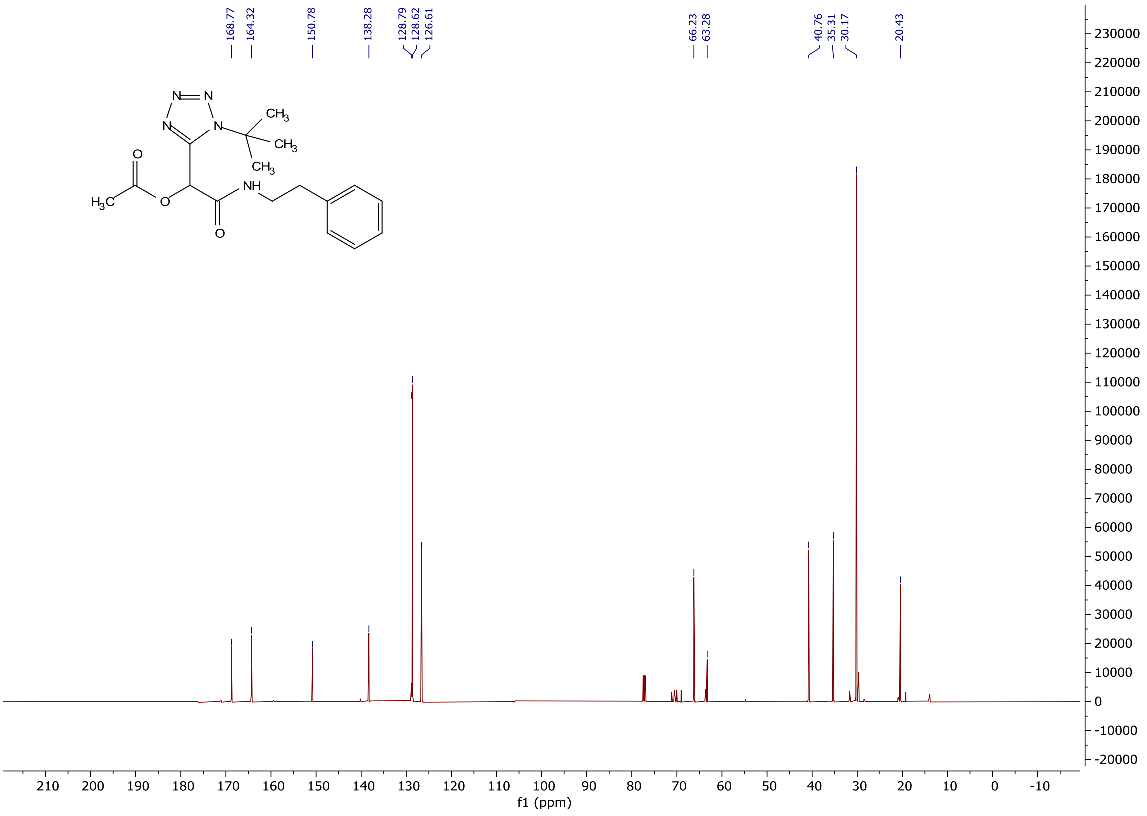

***N*-Benzyl-2-(*N*-(3,4-dimethoxyphenethyl)acetamido)-2-(1-(2,4,4-trimethylpentan-2-yl)-1*H*-tetrazol-5-yl)acetamide (4a)**

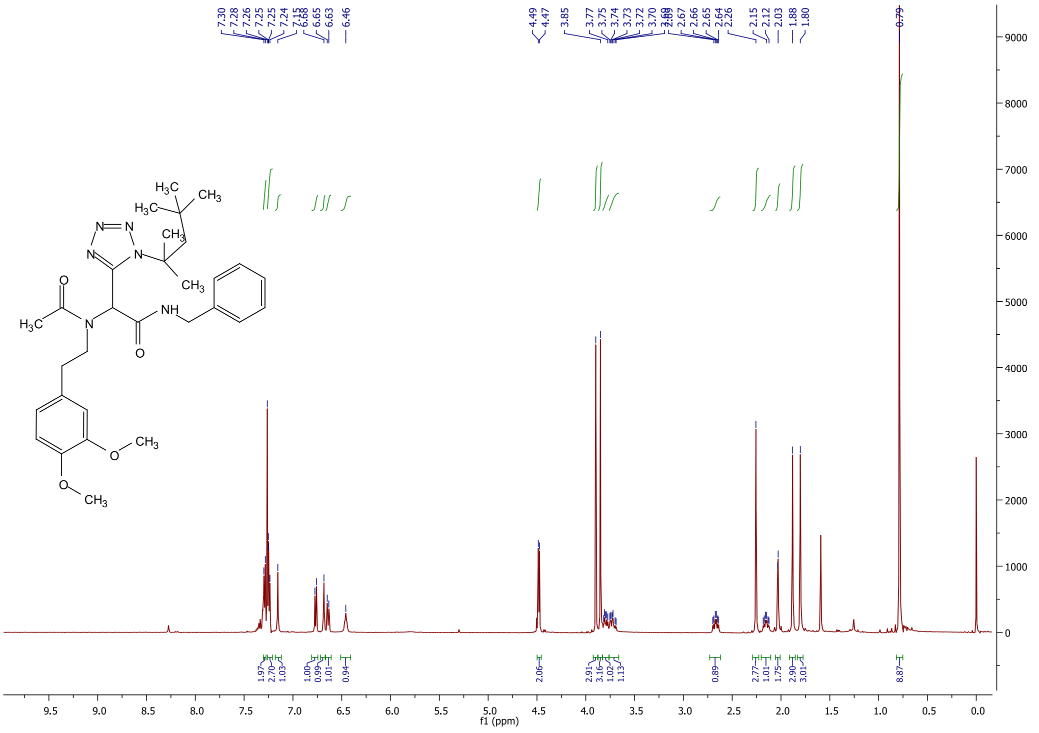

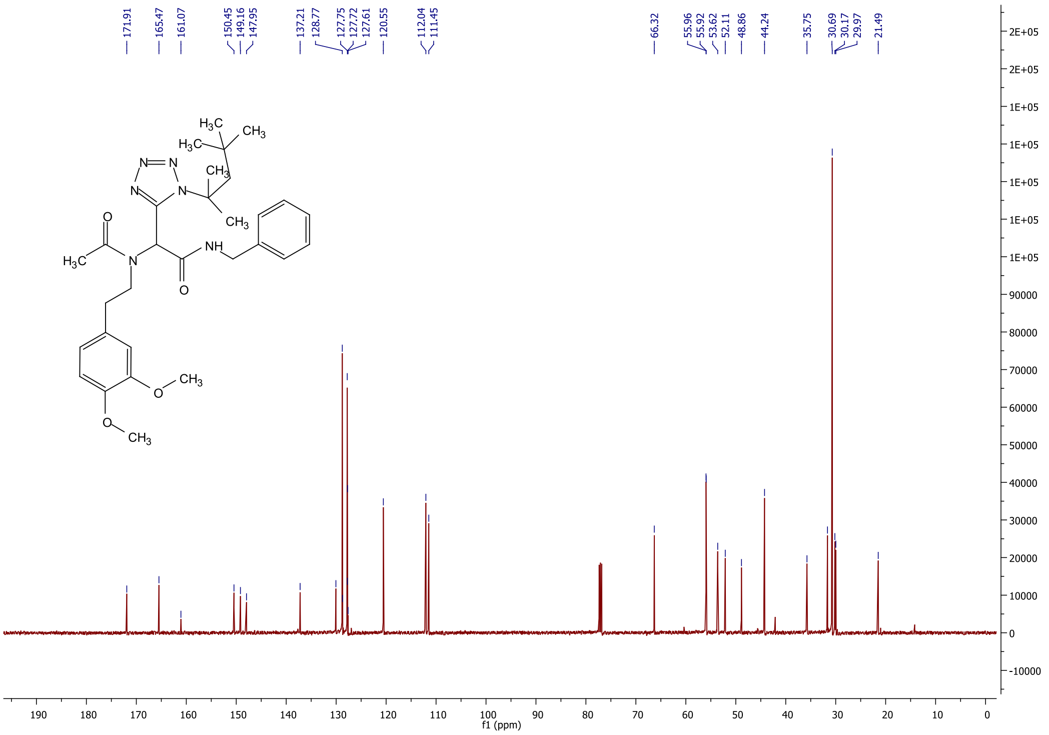

***N*-Cyclohexyl-2-(*N*-phenethylacetamido)-2-(1-(2,4,4-trimethylpentan-2-yl)-1*H*-tetrazol-5-yl)acetamide (4b)**

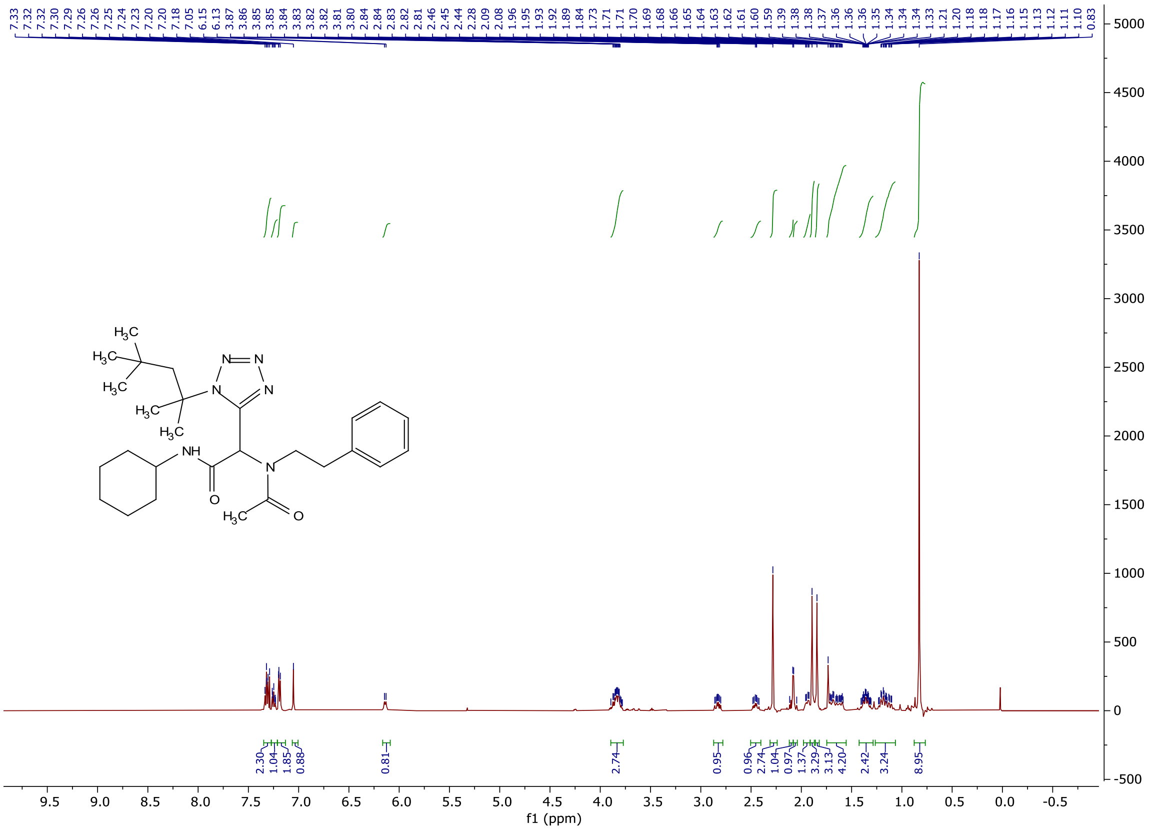

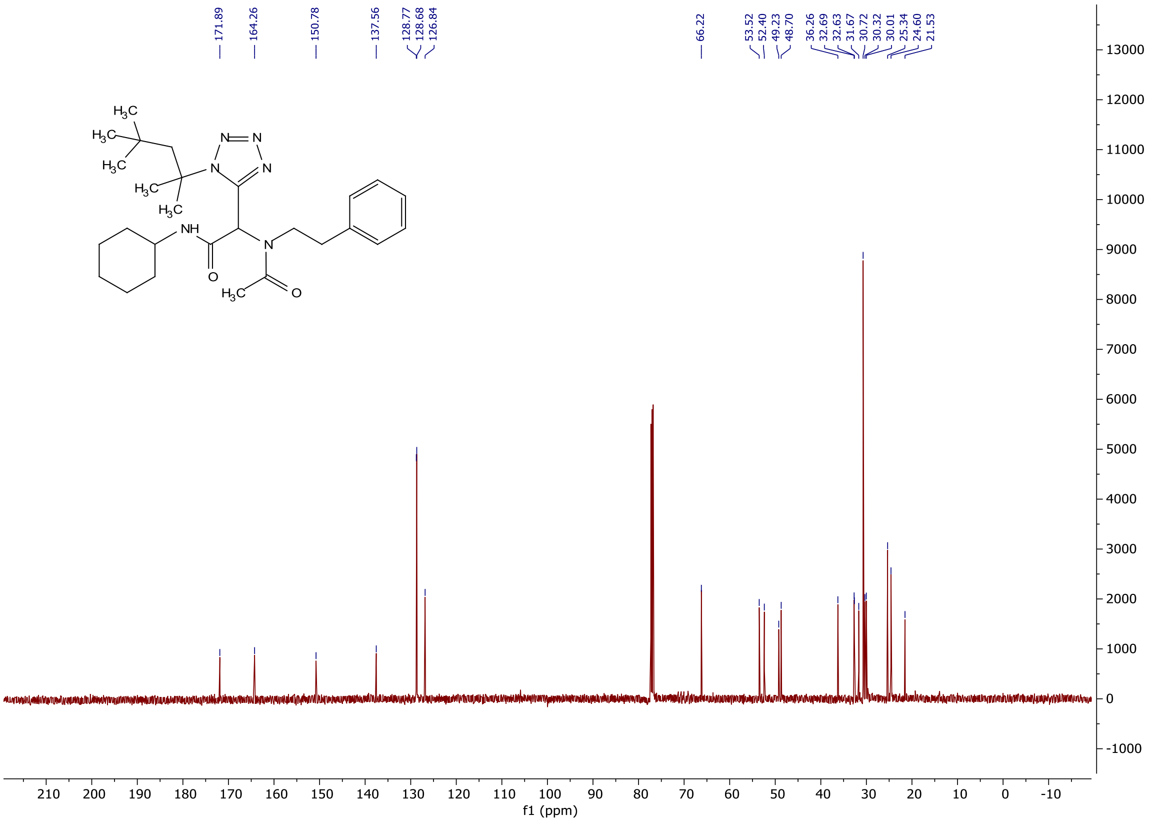

N-Benzyl-2-(*N*-butylacetamido)-2-(1-(2,4,4-trimethylpentan-2-yl)-1*H*-tetrazol-5-yl)acetamide (4c)

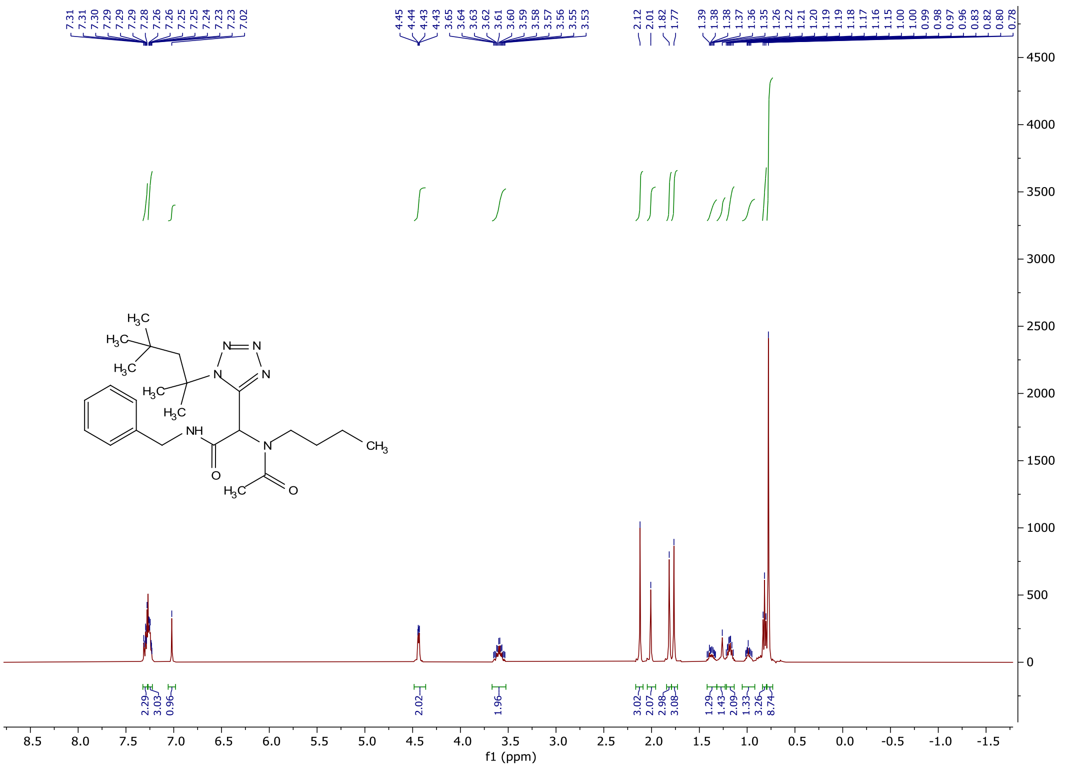

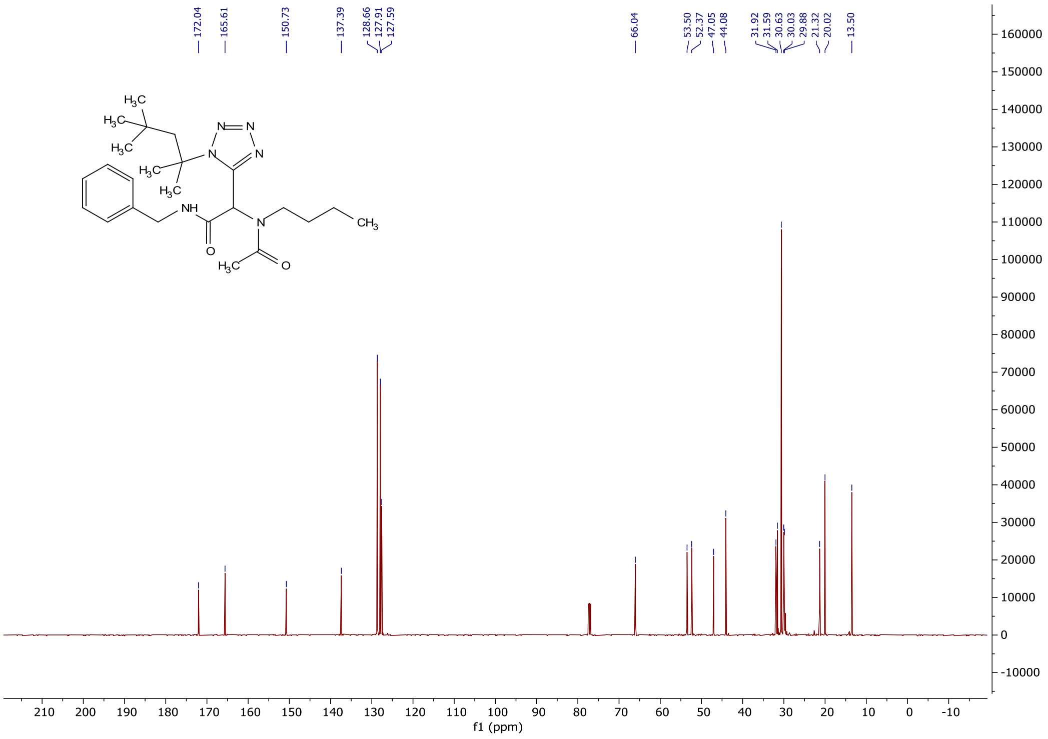

***N*-Benzyl-2-(*N*-benzylacetamido)-2-(1-(2,4,4-trimethylpentan-2-yl)-1*H*-tetrazol-5-yl)acetamide (4d)**

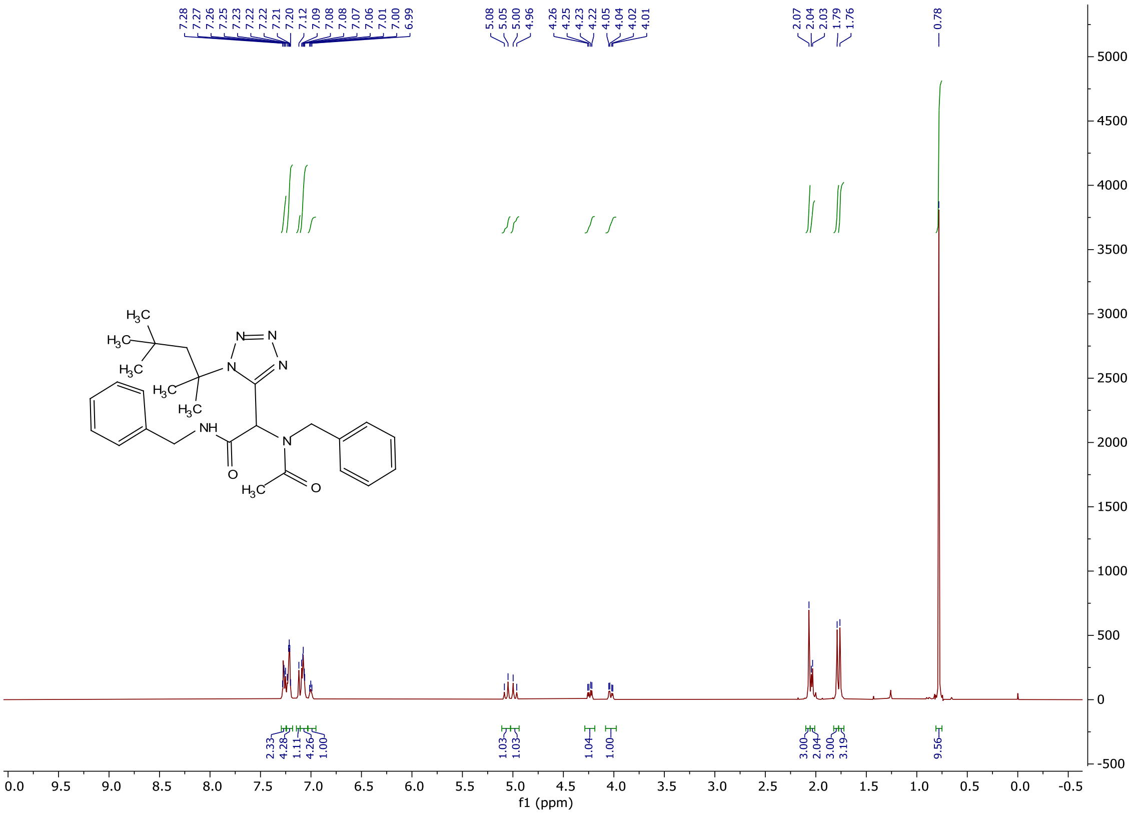

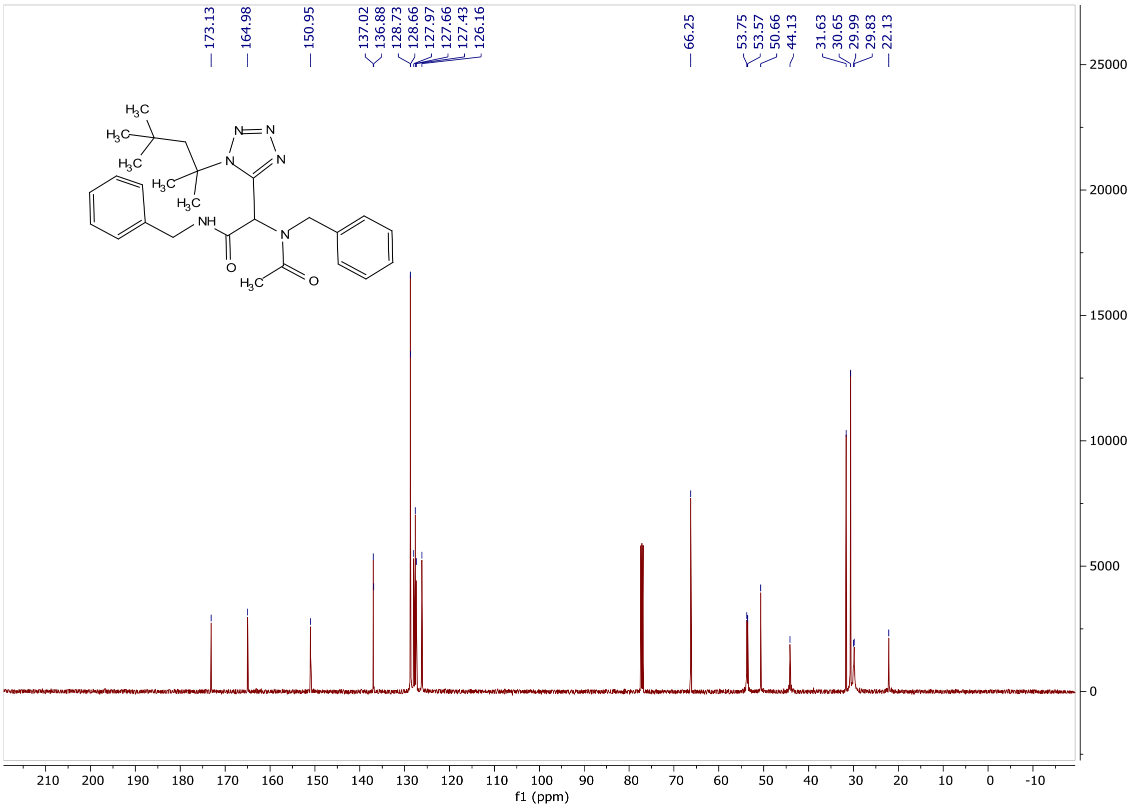

_N_-Benzyl-2-(1-(_tert_-butyl)-1_H_-tetrazol-5-yl)-2-(_N_-(3,4-dimethoxyphenethyl)acetamido)acetamide (4e)

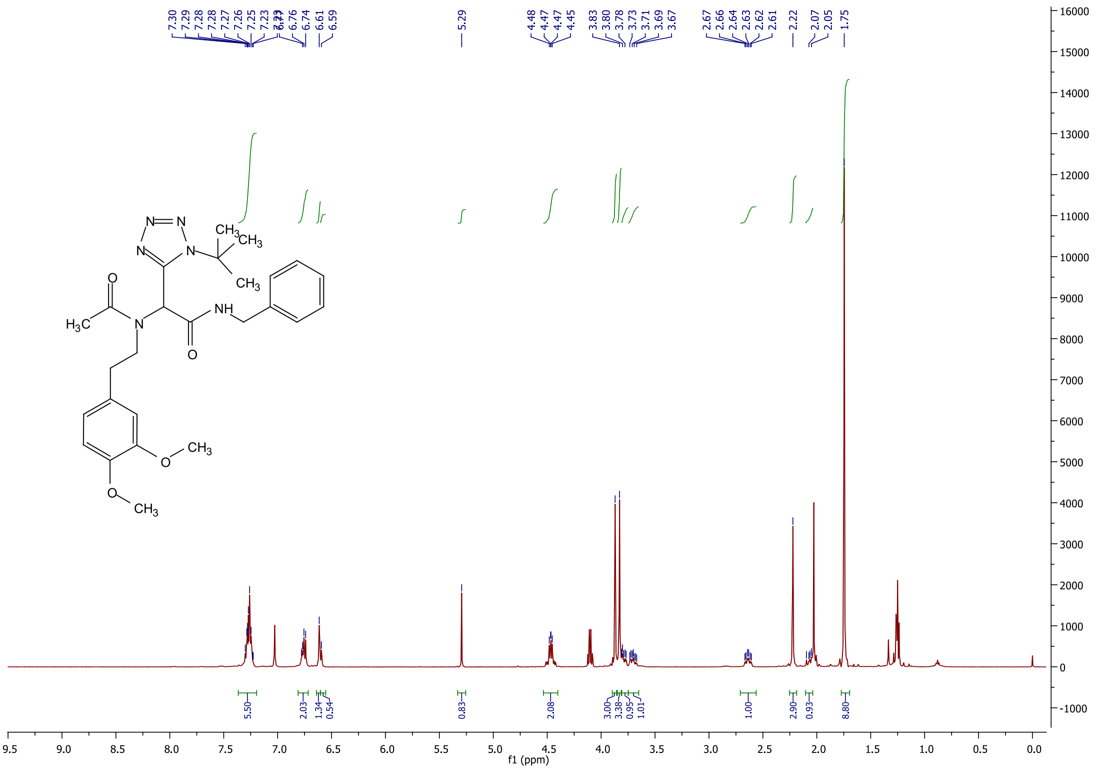

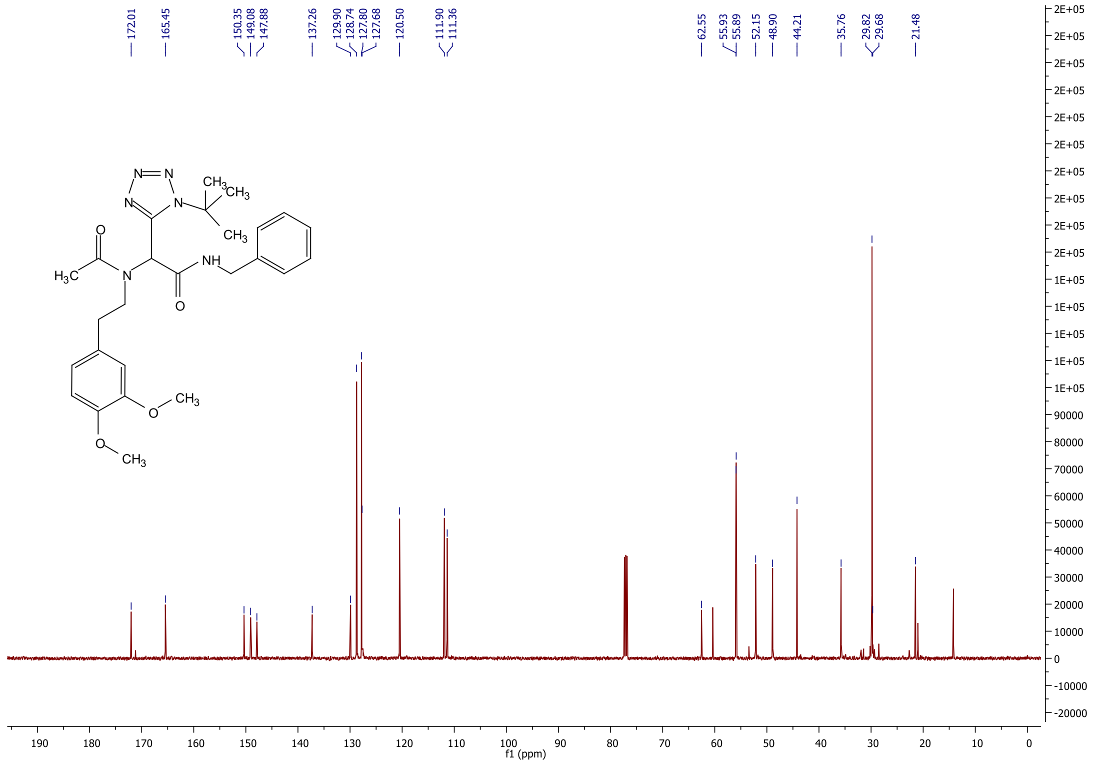

N-Benzyl-2-(1-(*tert*-butyl)-1*H*-tetrazol-5-yl)-2-(*N*-isopropylacetamido)acetamide (4f)

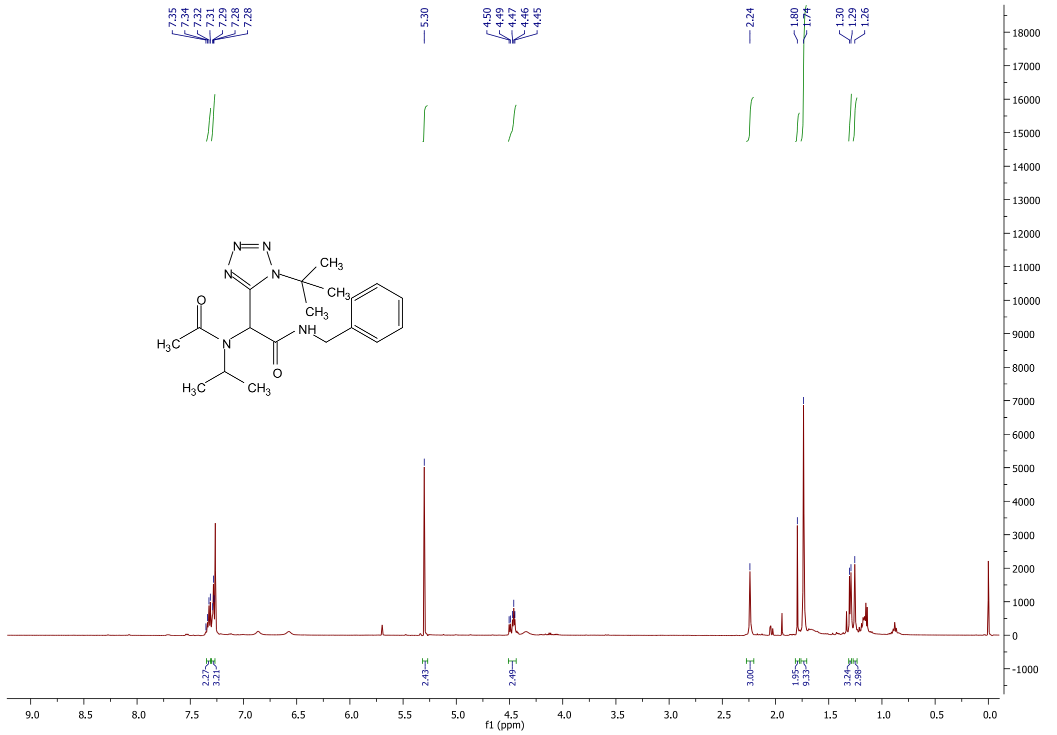

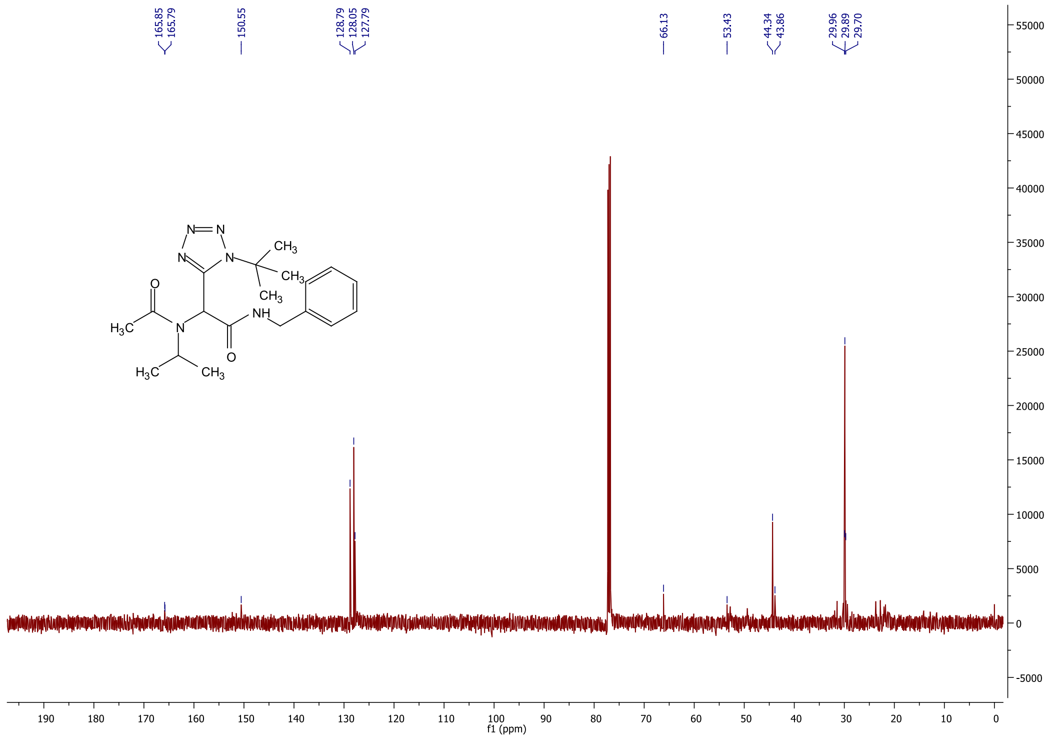

5,5'-(Pyrrolidin-1-ylmethylene)bis(1-(2,4,4-trimethylpentan-2-yl)-1*H*-tetrazole) (5a)

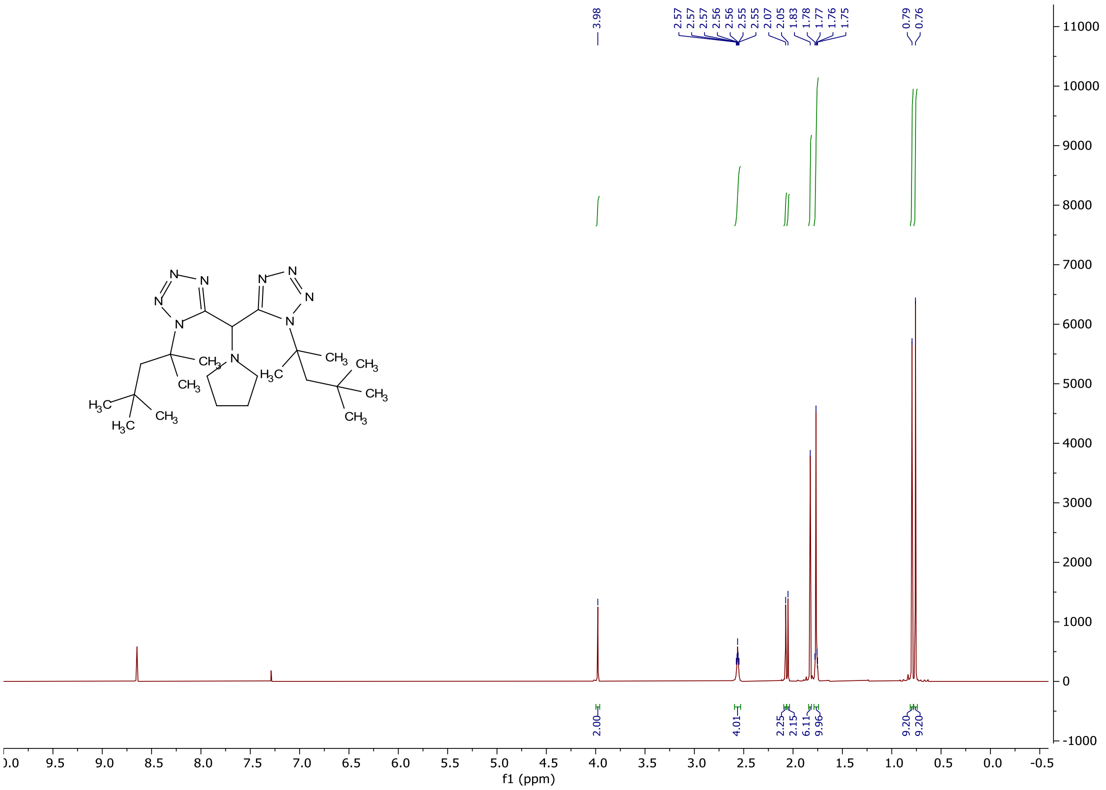

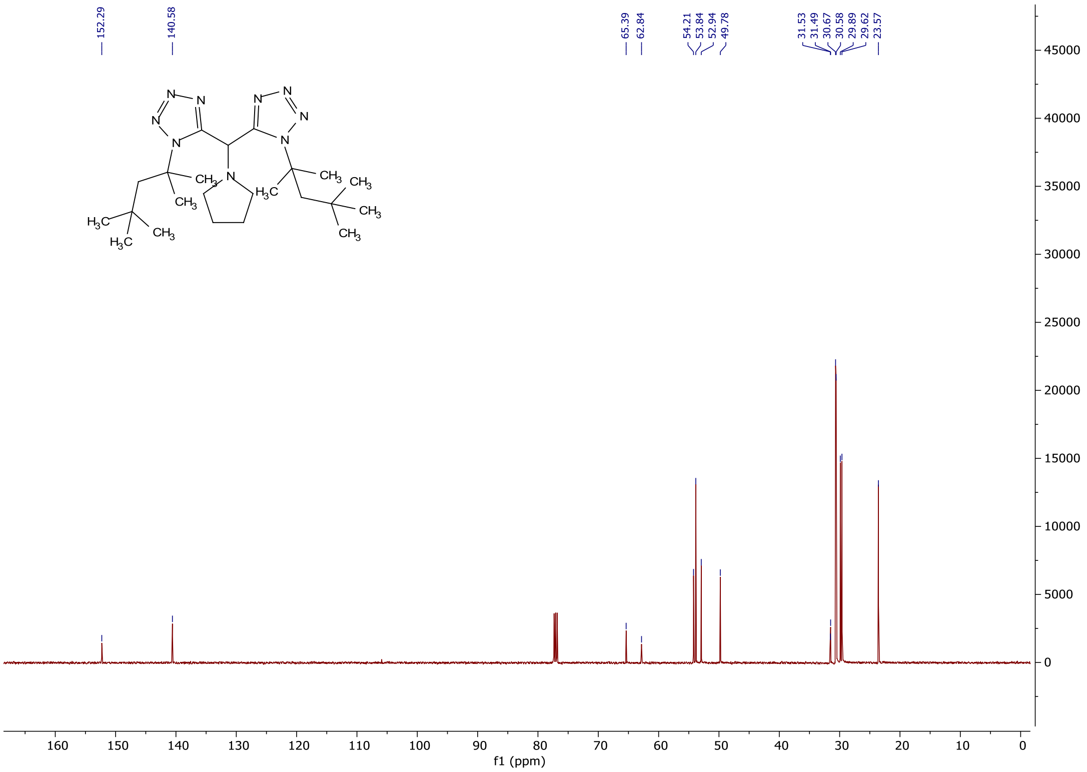

_N_-((1-Benzyl-1_H_-tetrazol-5-yl)(1-(_tert_-butyl)-1_H_-tetrazol-5-yl)methyl)-2-phenylethan-1-amine (5b)

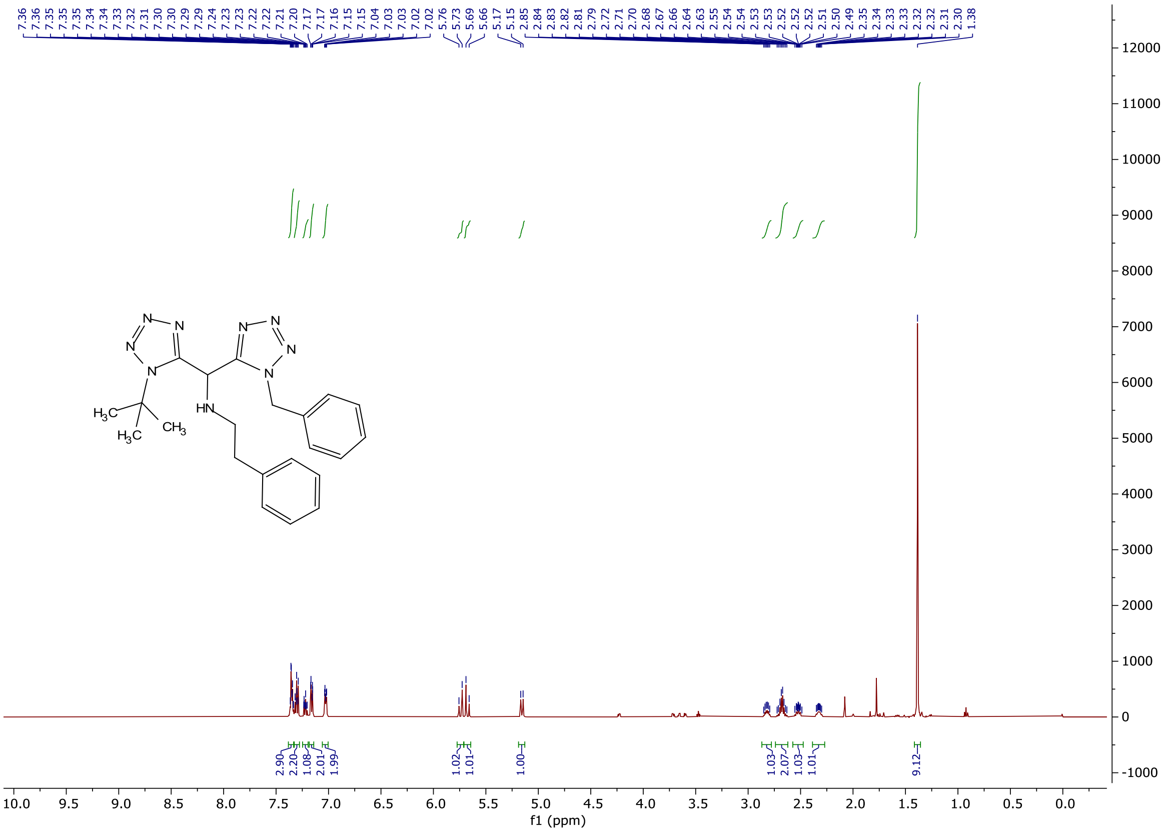

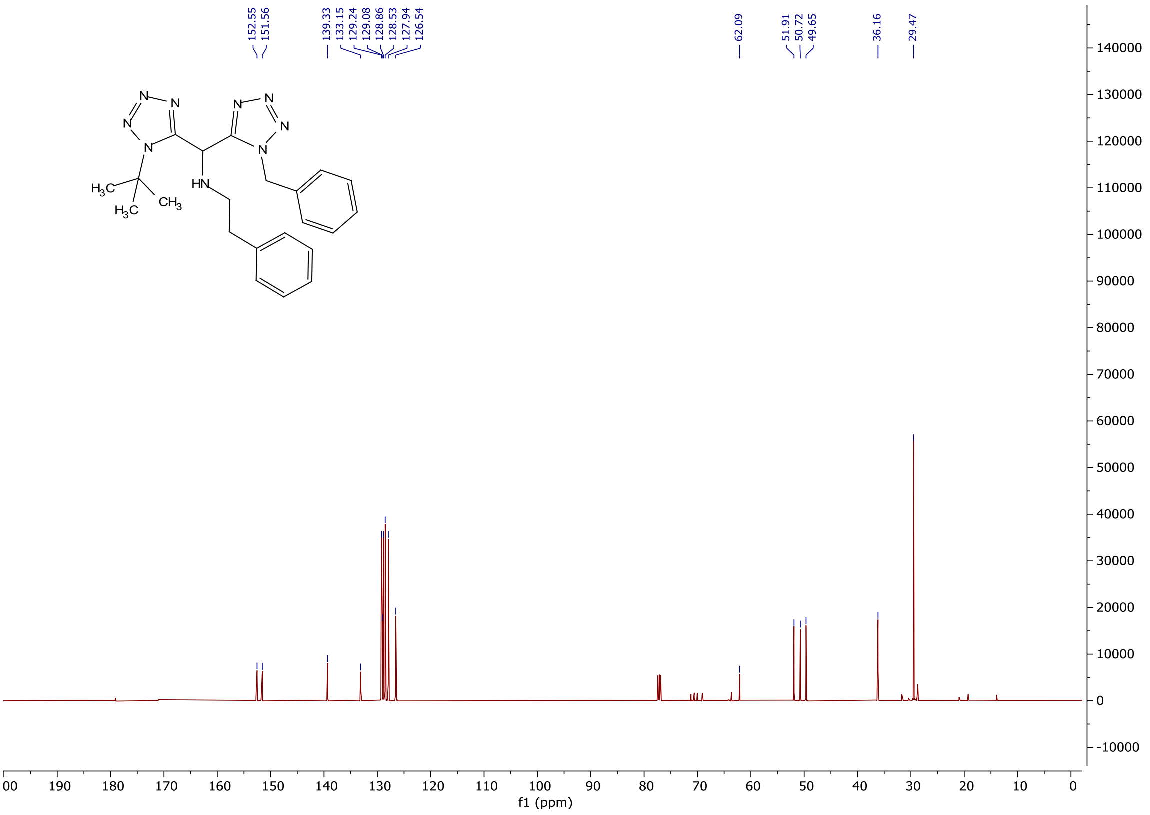

***N*-(Bis(1-(*tert*-butyl)-1*H*-tetrazol-5-yl)methyl)butan-1-amine (5c)**

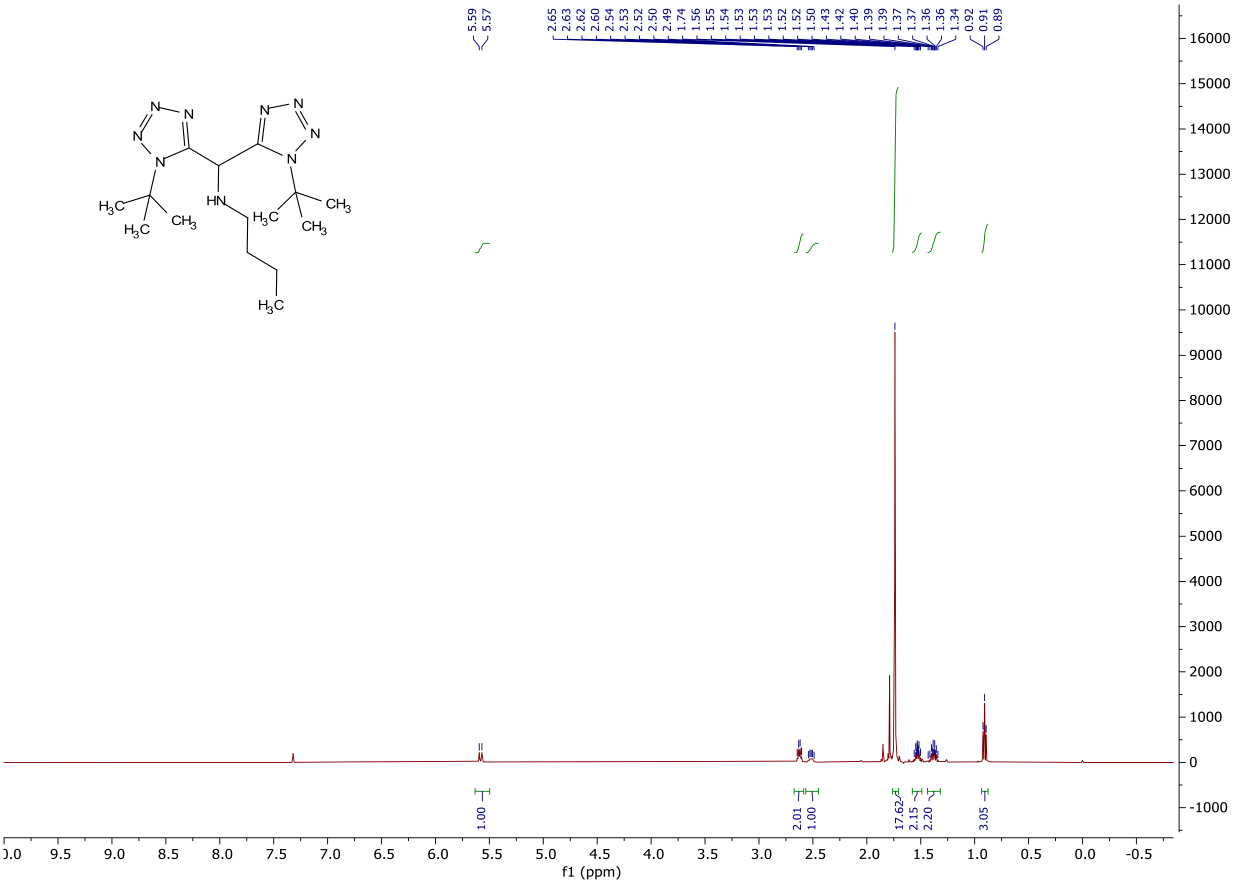

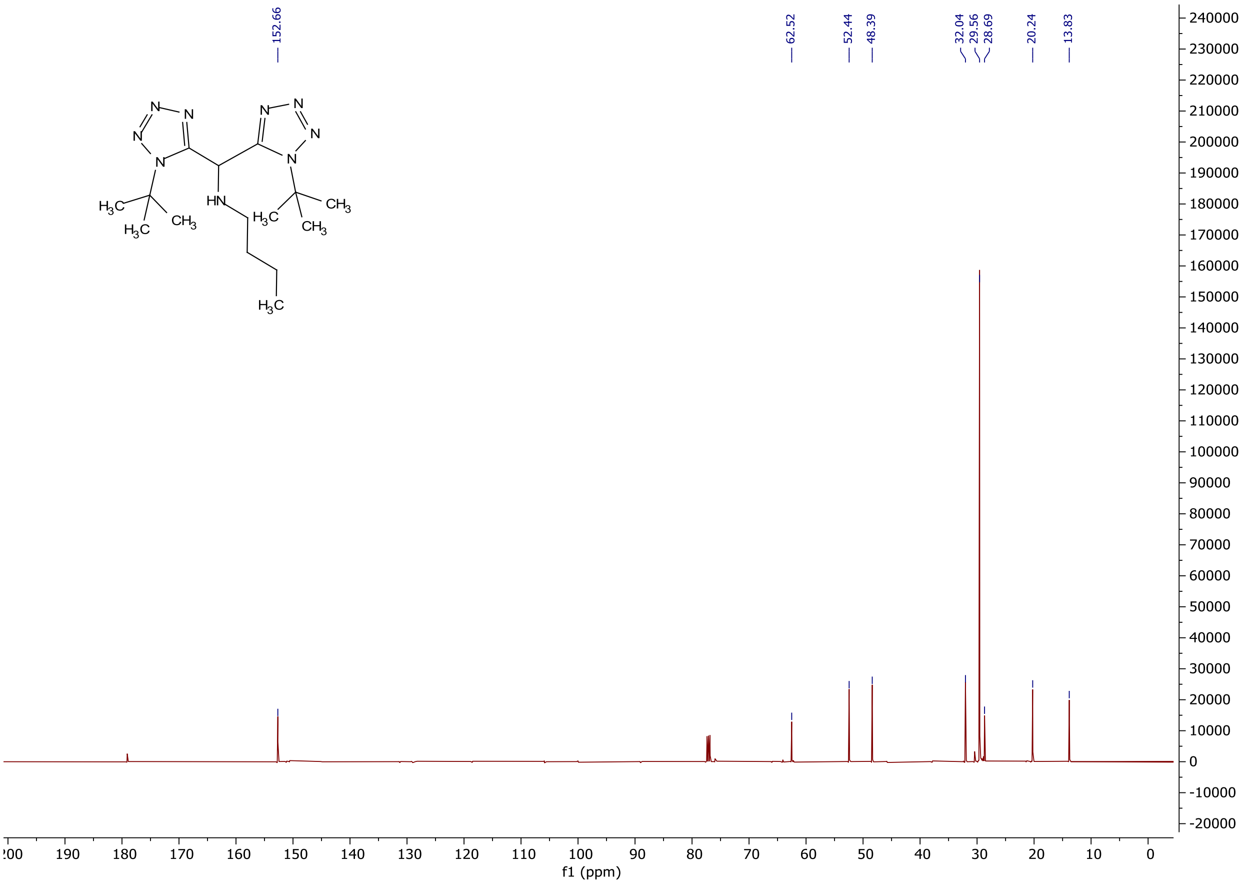

1*H*-Tetrazole-5-carbaldehyde (6)

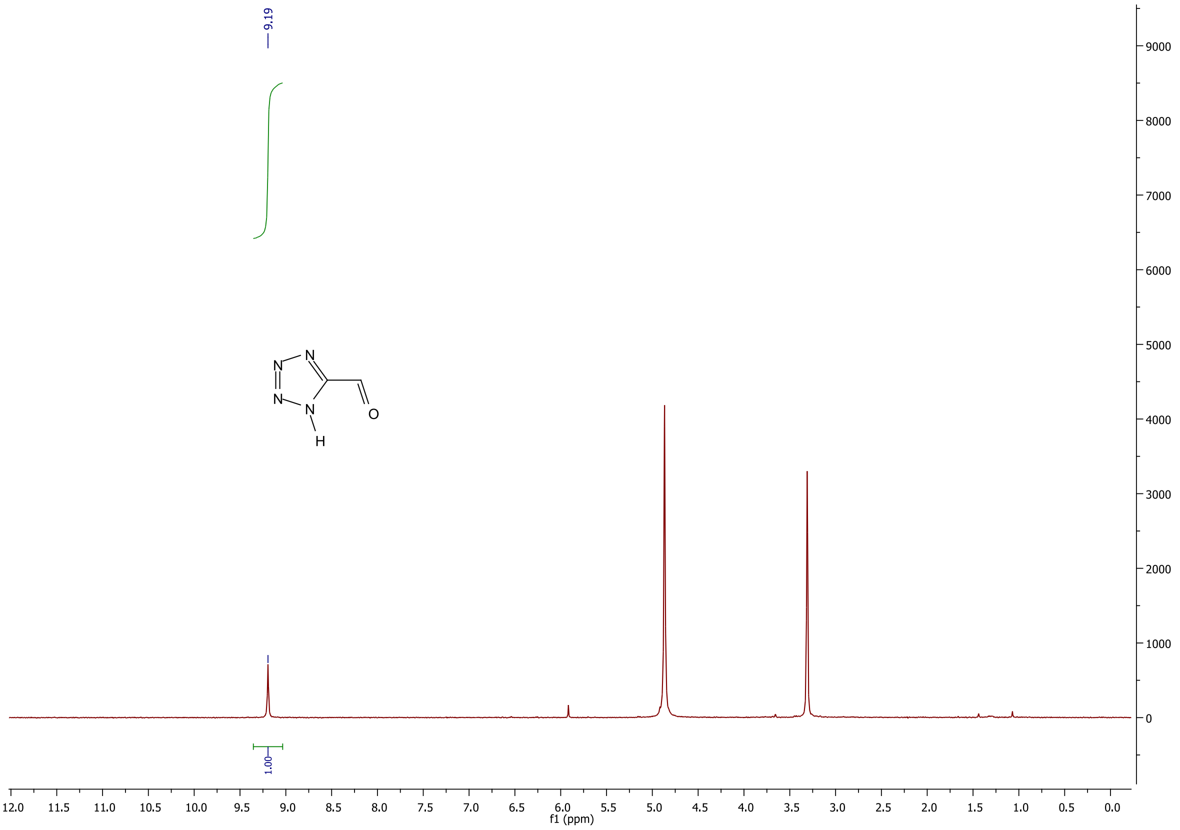

Supplement: File 1 — Experimental procedures, compound characterizations, and NMR spectra. [file Beilstein_J_Org_Chem-20-950-s001.pdf]
